# UAMP: Consistent video object segmentation with uncertainty-aware memory propagation

**DOI:** 10.1371/journal.pone.0353156

**Published:** 2026-07-07

**Authors:** Yichuang Luo, Fang Wang, Xiaohu Liu

**Affiliations:** 1 Department of Intelligent Science and Engineering, Xi’an Peihua University, Xi’an, China; 2 Trine Engineering Institute, Shaanxi University of Technology, Hanzhong, China; Hallym University, KOREA, REPUBLIC OF

## Abstract

The Segment Anything Model 2 (SAM 2) has emerged as a robust foundational model for video object segmentation. Nevertheless, it exhibits notable limitations in crowded scenarios, particularly those involving fast-moving objects or self-occlusion. Furthermore, its greedy-selection memory architecture is afflicted by “error accumulation,” which collectively impairs its performance in consistent object segmentation tasks. To address these critical drawbacks and enhance the consistency and robustness of SAM 2 in complex video scenarios, an enhanced variant of SAM 2, termed UAMP, is proposed, which integrates memory propagation based on explicit appearance and motion uncertainty modeling, coupled with a dual mechanism encompassing long-term memory updating and short-term memory selection. By fusing uncertainty-aware representations with these dual memory mechanisms, UAMP effectively accommodates dynamic variations in object appearance and motion, further refines the object memory bank, and thereby realizes consistent video object segmentation. Quantitative and qualitative evaluations conducted on diverse benchmark datasets demonstrate that UAMP achieves superior performance, particularly in scenarios involving occlusions and object reappearances. Specifically, UAMP yields consistent performance improvements over state-of-the-art (SOTA) methods across five video object segmentation (VOS) benchmarks, with a maximum enhancement of 5.6 points in the J&F metric. These findings underscore the robustness and effectiveness of the proposed UAMP method in addressing complex tracking scenarios, thus providing a valuable enhancement to SAM 2 for practical video object segmentation applications.

## 1 Introduction

Recently, Segment Anything Model 2 (SAM 2) [1] incorporates a streaming memory architecture, which empowers it to process video frames in a sequential manner while preserving object contextual information. While SAM 2 has demonstrated remarkable performance in Video Object Segmentation (VOS) [2] tasks, generating precise pixel-level masks for objects throughout a video sequence, it still may fail to maintain consistent segmentation across shot transitions or confused objects occurring in crowded scenes after long occlusions, and struggle with accurately tracking objects that are in close proximity to other entities with highly similar visual appearances.

Specifically, SAM 2 adopts a greedy strategy to select the mask with the highest predicted Intersection over Union (IoU) score [1]. A critical limitation of this approach is that if an incorrect mask is selected and stored in the model’s memory, it will introduce misleading information for the segmentation of subsequent frames, ultimately leading to cumulative error propagation [3]. Additionally, SAM 2 tends to prioritize appearance information over spatial and temporal cues, which may result in inconsistent segmentation in scenarios involving rapid object movement and object occlusion in crowded scenes [4]. Thirdly, SAM 2 employs a fixed-window memory mechanism with a first-in-first-out (FIFO) buffer [1]; this poses considerable challenges in scenarios where the target leaves and re-enters the field of view, or where the target is in close proximity to other objects with similar visual features [5].

Numerous studies have conducted various analyses to enhance the performance of SAM 2. SAM2Long [3] aimed to address the failure of SAM 2’s FIFO fixed-window memory mechanism in handling target reappearance by introducing a hierarchical memory tree structure for flexible memory retrieval and management, though it suffered from increased memory overhead, potential retrieval latency, and failed to resolve the confusion between similar-looking instances during memory retrieval. SAMURAI [4] integrated Kalman filtering into SAM 2’s memory update process to model target motion trajectories and suppress error accumulation through a prediction-update mechanism, yet its performance was heavily dependent on motion prediction accuracy in fast-changing scenarios, and it could not effectively handle severe target deformation that led to large prediction errors. DAM4SAM [5], proposes a novel distractor-aware memory (DAM) module for the SAM 2 model, which is composed of recent appearance memory (RAM) and designed to address the interference of distractor objects (irrelevant objects with similar appearance to the target) in VOS tasks. However, DAM4SAM over-relies on the recent appearance features stored in RAM, making it less adaptable to scenarios where the target undergoes significant appearance changes over time. DC-SAM [6] leverages multi-source features and generates positive and negative prompts by ensuring prompt consistency, integrating with SAM/SAM2 to achieve in-context segmentation for both images and videos. and improves the model's adaptability to complex scenes. But it focuses on prompt-tuning adaption, and lacks effective long-term temporal correlation modeling capabilities, making it difficult to maintain consistent segmentation performance when the target undergoes continuous appearance changes (such as scale variation, deformation) in long video sequences.

To address these limitations, we integrate the appearance and motion uncertainty into the SAM 2’s prediction process with long-term memory updating and short-term memory selection mechanism. By leveraging the historical trajectory of the tracking objects, the motion information can be effectively estimated to maintain the tracking accuracy in occlusion situation. Additionally, the appearance uncertainty of the current frame is predicted relative to the preceding frame which further guides the feature fusion of the tracking objects. Specifically, the significant changes regions heavily on the information of current frame, while the minimal variants remain the memory feature from previous frame. This adaptive memory fusion inherently stabilizes the memory propagation across the video sequence, owing to the more detailed object-centric features and temporal consistency [7]. Furthermore, the combined mechanism with long-term and short-term memory is utilized to address the distracting challenge with similar visual features. In which, the long-term memory is updated in a recurrent manner with linear attention to retain the long historical information, and the selection mechanism is applied in the short-term memory to further resist the disturbance of surrounding objects [4,5].

To this end, we propose UAMP, a SAM2-adapted consistent video object segmentation unifying motion information and appearance uncertainty with long-term memory and short-term memory selection mechanism. Our proposed method incorporates two key advancements: (1) feature refinement with appearance and motion uncertainty estimation, which enables more fine-grained feature representation of the tracking objects, based on object position prediction and appearance uncertainty estimation, and (2) consistent feature propagation with long-term memory updating and short-term memory selection mechanism, which leverages the hybrid scoring system, combining the original mask affinity, object, and motion scores to retain more tracking object-relevant information to enhance the distraction of surrounding similar objects or incorrect segmentation.

In summary, our main contributions are as follows: 1) We propose UAMP, a consistent video object segmentation model, which is specifically designed to address the inherent limitations of SAM 2 in long-term VOS tasks, with a core uncertainty-aware memory propagation framework, providing a reusable module for optimizing memory-based video segmentation models. 2) We introduce an uncertainty-aware memory propagation mechanism by the appearance and motion uncertainty estimation to refine the feature representation. At the meantime, a long-term memory updating mechanism and score-based short-term memory selection is designed to reduce the disturbance in crowded scenes by a mixture of motion and affinity scores. 3) Our UAMP achieves state-of-the-art performance on SA-V, MOSE, LVOSv1 and LVOSv2, and other VOT benchmarks, demonstrating effectiveness across diverse datasets.

## 2 Related works

**Video Object Segmentation (VOS)**. The VOS task takes an object mask as input in the first frame, the goal is to track and segment the corresponding object consistently throughout the entire video sequence [2]. The task is usually categorized as “semi-supervised VOS” since the input mask serves as a supervision signal of the target. This task has garnered substantial research attention, driven by its broad applicability in practical scenarios such as video editing and robotic vision systems.

Early deep learning-based approaches for VOS typically relied on online fine-tuning strategies to adapt models to the target object: some methods only performed fine-tuning on the first video frame [8,9], while others extended fine-tuning to all frames in the sequence [10]. Subsequent advancements shifted toward offline-trained models to achieve faster inference speeds. These offline models adopted different conditioning strategies: some were conditioned solely on the first frame [11], while others incorporated additional conditioning from the preceding frame [12]. To further enhance temporal consistency, this conditioning paradigm was expanded to leverage information from all past frames, with implementations based on Recurrent Neural Networks (RNNs) [13] and Transformers [14,15]. With the rise of vison foundation models, prompt-based visual segmentation method [1] have emerged as competitive alternatives. These methods achieve significant performance by accepting user-defined prompts (e.g., click points, bounding boxes, or masks) on any frame of the video sequence. In this paper, we explore the SAM 2 [1] on the application in semi-supervised VOS. Specially, the memory design of SAM 2 is enhanced, which significantly improves the consistency of object-centered information propagation.

**Memory-based VOS.** Objects in the long video sequence often undergo deformation [16], dynamic motion [17], reappearing [18], and suffer from occlusion [19], which make the VOS remain an unsolved task. To address these challenges, the memory-based paradigm [20] is proposed to enhance the temporal consistency across adjacent frames, in which the memory mechanism is adopted to store object information from past frames.

Recent advances in this field have focused on developing efficient memory reading mechanisms, which rely on either pixel-level attention [21] or object-level attention [22]. A representative method is XMem [20], which leverages a hierarchical memory structure for pixel-level memory reading. Building on XMem’s foundational framework, Cutie [22] further improves segmentation accuracy by processing pixel features at the object level, enabling more robust handling of complex scenarios. The latest SAM 2 [1] adopts a more streamlined approach by selecting the temporally nearest frames as memory, but it struggles with long-term reappearing objects and confusingly similar objects. To mitigate this limitation, SAM2Long [3] introduces a tree-based memory structure to enhance segmentation performance for long videos. Nevertheless, deeper tree-based memory architectures incur a prohibitive cost, where exponentially more computational resources and memory storage are needed. To balance efficiency and performance, we enhance the SAM 2’s memory bank to propagate consistent object representations with long-term memory updating and short-term selection mechanism.

**Consistency modeling.** In VOS, the core challenge lies in preserving consistent object identity and location to resist occlusion, appearance variation, and the presence of visually similar objects. To strengthen temporal consistency across consecutive frames, techniques such as recurrent frame fusion [23] and temporal attention propagation [24] are widely adopted. And these approaches can be classified into two main categories: motion-based and appearance-based methods.

Motion-based models with learnable components have garnered growing attention, due to the data-driven characteristics. For instance, MotionTrack [25] improves tracking performance by learning representational features of past trajectories to predict future object movements. SAMURAI [4] integrates temporal motion cues with a proposed motion-aware memory selection mechanism, further enhancing tracking robustness. In contrast, appearance-based methods focus on distinguishing the tracked object from visually similar counterparts. DAM2SAM [5], for example, introduces a novel distractor-aware memory model tailored for SAM2, alongside an introspection-driven update strategy—this dual-design jointly optimizes both segmentation accuracy and tracking stability. MatAnyone [7] propagates object features through adaptive memory and image feature fusion, which incorporates memory information from the preceding frame to maintain consistency. DC-SAM [6] designs a cycle-consistent cross-attention on fused features and initial visual prompts, to enhance the features of the SAM’s prompt encoder in segmentation by providing high-quality visual prompts.

Building on these paradigms, we model the consistency for video segmentation by integrating motion-based and appearance-based methods into the memory-based paradigm, leveraging the strengths of both approaches to address VOS challenges more comprehensively.

## 3 Methods

### 3.1 Preliminary

To better contextualize our method, we first briefly review the core architecture of SAM 2. As illustrated in Fig 1, SAM 2 [1] comprises four main components: (1) an image encoder, (2) a mask decoder integrated with a prompt encoder, (3) a memory attention layer, and (4) a memory encoder coupled with a memory bank.

**Fig 1:**
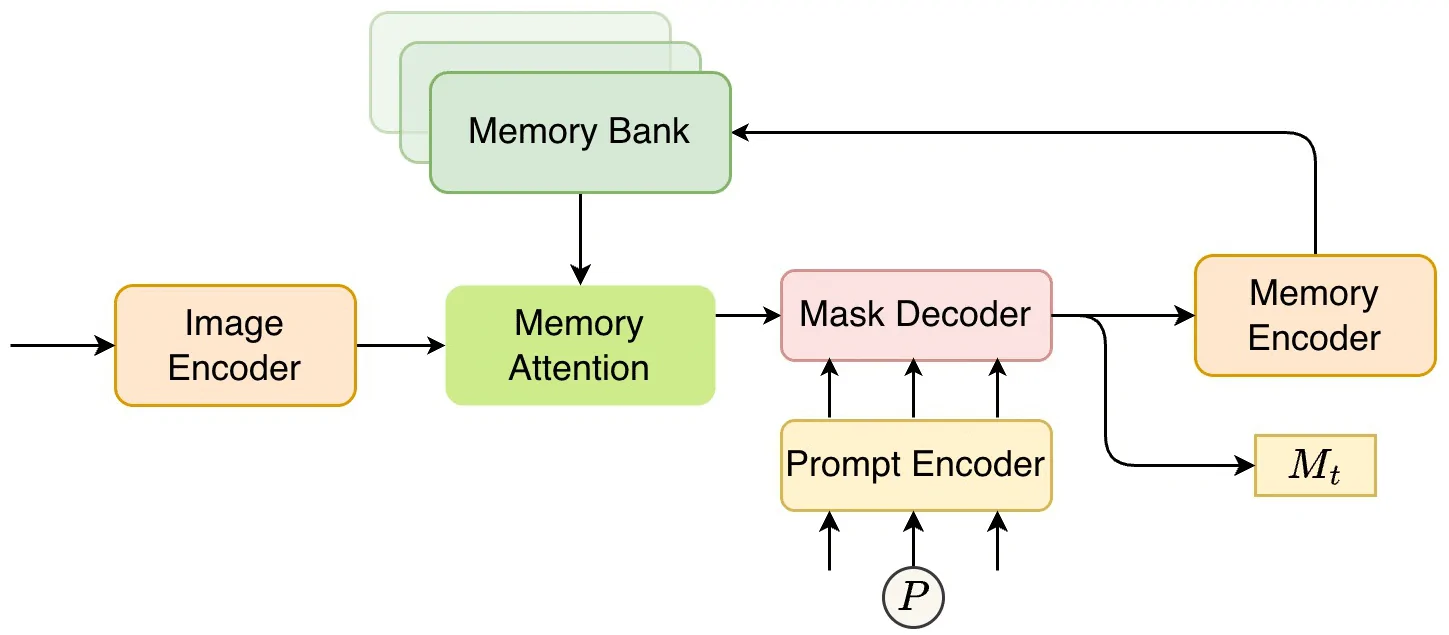
The illustration of SAM 2 architecture. It consists of the image encoder, memory attention, mask decoder and memory bank. And the prompt encoder is used to embed the prompts, such as boxes, points and text, as tokens.

Image encoder. The pre-trained Hiera [26] image encoder is employed, and the hierarchical architecture enables the utilization of multiscale features during the decoding phase, which is critical for capturing both fine-grained details and global context.

Prompt encoder and mask decoder. The prompt encoder embeds the prompts into 256-dimension tokens $E_{token}.\;$For video sequence, owing to occlusion or motion, ambiguities often persist across consecutive frames. To address this, the model predicts a set of candidate masks $M=\left\{\left(M_{\text{0}},\overset{t}{\underset{mask,\text{1}}{s}}\right),\left(M_{\text{1}},\overset{t}{\underset{mask,\text{2}}{s}}\right),\left(M_{T},\overset{t}{\underset{mask,\text{3}}{s}}\right)\right\}\;$for each frame. By default, the mask with highest predicted IoU score $\overset{\text{t}}{\underset{\text{mask}}{\text{s}}}$ is selected as the final mask. Additionally, an extra prediction head is introduced to output an object score ${\text{s}}_{\text{obj}}$, which indicates whether the target object of interest is present in the frame.

Memory attention. The current features are conditioned on the historical frame features by applying the transformer blocks. Each transformer block first performs self-attention on the current frame’s features to model internal dependencies, followed by cross-attention that aligns these features with the memory of previous frames to ensure the temporal consistency across the video sequence.

Memory encoder and Memory bank. The memory encoder generates frame-specific memory representations by down-sampling the features of each frame. Specifically, the memory bank maintains temporal continuity of target object information via the First-In-First-Out (FIFO) queue, which stores N recent features to preserve the historical information of the target object.

### 3.2 Extended methods based on SAM2 for VOS

Benefiting from the strong representation ability and flexible architecture of SAM 2, a series of extended methods have been proposed to improve video object segmentation (VOS) performance. According to their core improvement strategies, these works can be broadly categorized into three groups: path-searching methods, motion-estimation methods, and memory-adaptive methods.

#### 3.2.1 Path-searching method.

In SAM 2, the mask with the highest predicted IoU is selected. While this greedy strategy may increase the risk of errors when processing challenging frames to cause error accumulation.

To mitigate this problem, SAM2Long [3] enhances SAM 2 by integrating multiple memory pathways, as illustrated in Fig 2(A). This design enables the storage of diverse masks as memory at each time step, thereby improving the accuracy of predictions for subsequent frames. Within each pathway, an object-aware memory bank is constructed, this bank selectively incorporates the frames with confidently detected objects and high-quality segmentation masks. The specific mechanism of this process is formally described in Eq (1).

**Fig 2:**
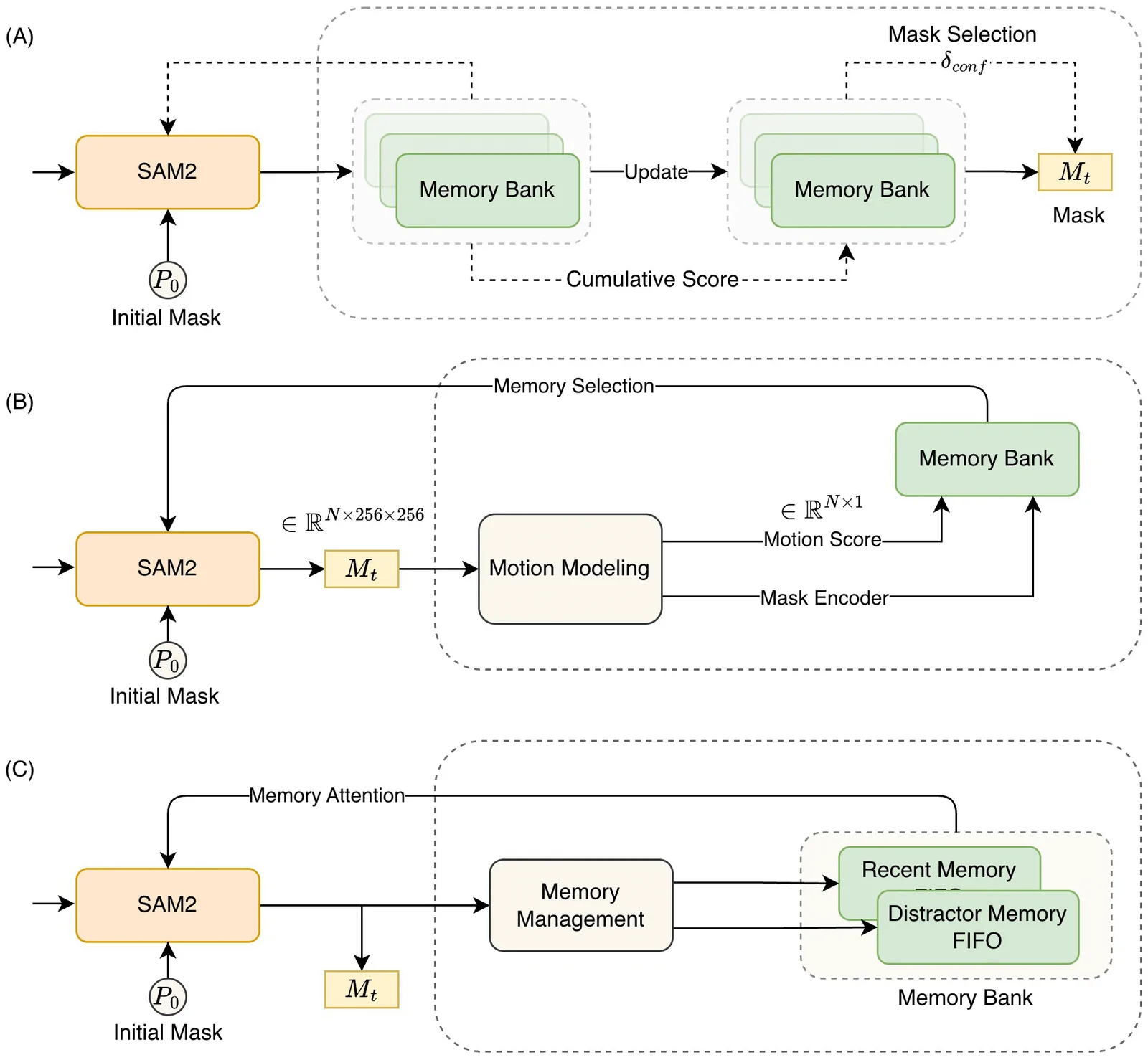
Existing methods based on SAM2 for video object segmentation. (A) Path-searching method. In which the multiple memory pathways are searched to include the confidently detected objects. (B) Motion-estimation method. Where the objects motion is modeled to enhance the score system. (C) Memory-adaptive method. In which the enhanced memory mechanisms are designed for object information consistency.


$$\begin{array}{c} \begin{array}{c} \overset{t}{\underset{mask,k}{s}}=s^{t-\text{1}}+\text{log}\left(\overset{t}{\underset{mask,k}{s}}+\epsilon \right),\;k\in \{\text{1},\text{2},\text{3}\}\\ \overset{i}{\underset{mem}{f}}=I\left(\overset{t}{\underset{mask,k}{s}}>\delta_{mask},\;\overset{t}{\underset{obj}{s}}>\text{0}\right)*f_{t},\;i\in \left\{\text{1},\text{2},\cdots ,N\right\}\;\\ f_{mem}=\omega_{i}\cdot \overset{i}{\underset{mem}{f}},\;i\in I\left(\overset{\text{1}}{\underset{obj}{s}}\leq \overset{\text{2}}{\underset{obj}{s}}\leq \cdots \leq \overset{N}{\underset{obj}{s}}\right) \end{array} \end{array}$$ (1)

where ε is a small constant to prevent the logarithm of zero, $f_{t}$ denotes the features of t-th frame, k is the mask index from SAM2 output three masks, $f_{men}$ represents the memory features encoded to the memory bank, and N = 7, and I is the indicator function, when the condition is met, it equals to 1, otherwise 0. In each memory pathway, an object-aware memory selection mechanism is designed to retrieve frames containing discriminative objects as described in the second formula in Eq (1). Concurrently, the calculation of memory attention is modulated to further enhance the model's focus on target objects, which is depicted in the third formula in Eq (1).

#### 3.2.2 Motion-estimation method.

The fixed-window memory mechanism in the SAM 2 model fails to account for the quality of memories. Specifically, objects with low confidence may be incorrectly encoded by the model, which may lead to error propagation in long video sequence. To tackle this problem, the SAMURAI [4] introduces two key enhancements, as illustrated in Fig 2(B). First, a Kalman Filter (KF)-based motion modeling module is applied to estimate the object motion, and second, an improved memory selection strategy is designed, which fuses both affinity and motion scores. These modifications are explicitly devised to boost the model’s capability to track objects accurately in complex video scenarios. The mathematical formulation of this process is provided in Eq (2).


$$\begin{array}{c} \begin{array}{c} z_{t}=\left[x,x^{\prime }\right],\;x=[x,y,a,h]\;\\ \begin{array}{c} \begin{array}{c} {\hat{z}}_{t+|t}=A{\hat{z}}_{t}\;\\ \;{\hat{z}}_{t+\text{1}}=\;{\hat{z}}_{t+\text{1}|t}+K\left(z_{t}-H{\hat{z}}_{t+\text{1}|t}\right) \end{array}\\ \begin{array}{c} \overset{t}{\underset{kf}{s}}=IoU\left({\hat{z}}_{t+\text{1}},\;M\right)\\ f_{mem}\;=I\left(\overset{t}{\underset{mask,k}{s}}>\delta_{mask},\;\overset{t}{\underset{obj}{s}}>\delta_{obj},\;\overset{t}{\underset{kf}{s}}>\delta_{kf}\right)*f_{t} \end{array} \end{array} \end{array} \end{array}$$ (2)

In which, z denotes the state of Kalman filter, which is consist of x, y, the top-left coordinate of the bounding box, a denotes aspect ratio, height h, and their corresponding velocity $x^{\prime }$, A is the linear state transition matrix, K represents the Kalman gain, and H is the observation matrix. Furthermore, to ensure the robustness of the motion modeling after the targeted object reappears or the poor mask qualities for a certain period of time, a stable motion state is maintained with confident threshold, as describe in the last formula in Eq (2).

#### 3.2.3 Memory-adaptive method.

When the tracking object is surrounded by similar objects in crowd scene, current VOS methods still face significant challenges. To address this issue, DAM4SAM [5] proposes a novel distractor-aware memory (DAM) module for the SAM2 model, which composes the recent appearance memory (RAM) $\overset{RAM}{\underset{mem}{f}}$ and distractor resolving memory (DRM) $\overset{DRM}{\underset{mem}{f}}$, as described as Eq (3).


$$\begin{array}{c} \begin{array}{c} \begin{array}{c} \overset{RAM}{\underset{mem}{f}}=I\left(\;\overset{t^{\prime }}{\underset{obj}{s}}>\text{0};t^{\prime }=t-\Delta \right)*f_{t}\\ {\overset{―}{A}}_{mask}=\frac{\text{1}}{N_{m}}A_{mask} \end{array}\\ \overset{DRM}{\underset{mem}{f}}=\;I\left(\overset{t}{\underset{mask,k}{s}}>\delta_{mask},\;\overset{t}{\underset{obj}{s}}>\text{0},\;A_{mask}<\delta_{area}\cdot {\overset{―}{A}}_{mask}\right)*f_{t} \end{array} \end{array}$$ (3)

As formulated in Eq (3), the memory is updated every ∆ = 5 frames and always incorporates the most recent frame containing object $\;\overset{t^{\prime }}{\underset{obj}{s}}>\text{0}$. For DRM, a bounding box is fitted to al the masks, and if the ratio between the area $A_{mask}$ of the two bounding boxes drops below 0.7, the current frame is considered as a potential candidate for updating DRM. In DAM4SAM, the DRM is updated during periods with sufficiently stable tracking, as depicted in the last formula in Eq (3). The criteria consist of the predicted mask with a threshold $\delta_{mask}=\text{0}.\text{8}$ and the mask area with $\delta_{area}$. And the ${\overset{―}{A}}_{mask}$ denotes the median mask area $A_{mask}$ in last $N_{m}=\text{1}\text{0}$ frames.

### 3.3 Adapting SAM2 with uncertainty-aware memory propagation

To address the issue of error accumulation, preserve consistency, and maintain segmentation accuracy for long video sequences, we propose the uncertainty-aware memory propagation module, which integrates the appearance and motion uncertainty estimation with a long-term and short-term memory selection mechanism. Specifically, appearance uncertainty is estimated to facilitate the fusion of object features, and a hybrid scoring mechanism, combining affinity and motion scores, is employed for memory selection. The architecture of the proposed model is illustrated in Fig 3.

**Fig 3:**
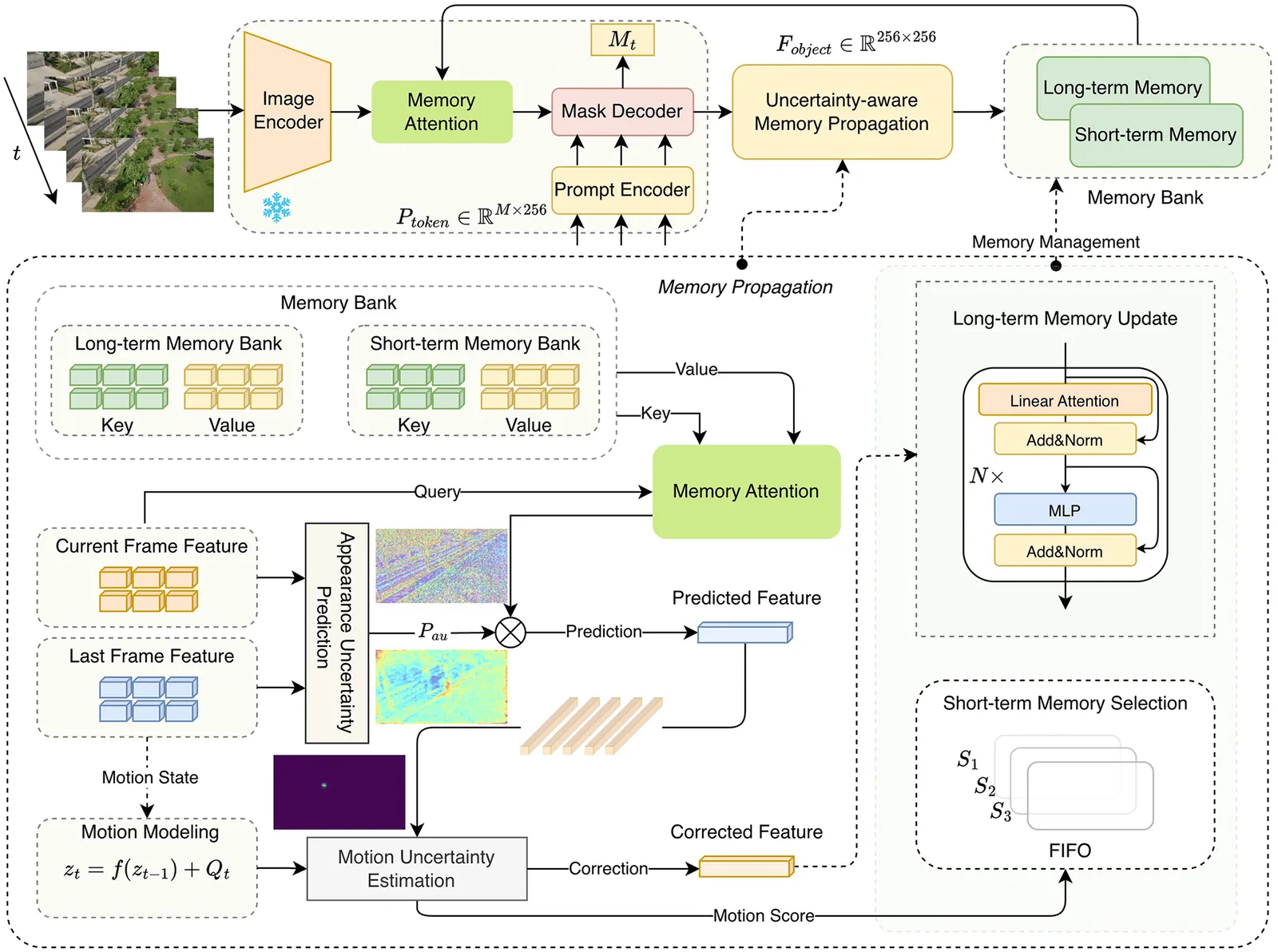
The architecture of proposed UAMP model. In which the uncertainty-aware memory propagation module is devised based on appearance and motion uncertainty estimation, with the long-term memory updating and short-term memory selection mechanism.

As shown in Fig 3, the uncertainty-aware memory propagation module consists of appearance uncertainty prediction, motion uncertainty estimation and memory managements blocks, which are detailed in the following sections. Specifically, the uncertainty module dynamically weights unreliable features caused by occlusion, motion blur, and clutter, suppressing noisy or ambiguous representations before they enter the memory system. And the dual long‑short term memory structure maintains both global temporal context and local frame‑to‑frame consistency, preventing error accumulation and drift in long sequences.

#### 3.3.1 Appearance uncertainty prediction.

In SAM 2, the memory values associated with the query frame are computed based on the similarity between the query and memory keys, with the underlying assumption that all query tokens carry equal importance. Usually, compared with frame t-1, only a small subset of tokens in current frame t exhibit significant changes. To address challenges posed by appearance variations, query tokens are weighted by the appearance uncertainty to enhance the model’s stability.

Specifically, the appearance uncertainty prediction module is introduced to estimate the uncertainty $U_{t}$ for each query token, enabling the adaptive memory and image feature fusion. The prediction module is designed to be lightweight, comprising merely three convolution layers. We formulate the prediction task as a segmentation problem, optimized using a segmentation loss $L_{seg}$. The supervision label is derived from the mask differences between frame t − 1 and frame t, and the sigmoid function is applied to transform the prediction as a probability. The final features are obtained via a soft merge of memory readout and last features, formally defined in Eq (4):


$$\begin{array}{c} f_{t}=\overset{mem}{\underset{t}{f}}\cdot U_{t}+f_{t-\text{1}}\cdot (\text{1}-U_{t}) \end{array}$$ (4)

Where ${\text{U}}_{\text{t}}\in [\text{0},\text{1}]$ represents the predicted appearance uncertainty, $\overset{mem}{\underset{t}{f}}$ is the current features queried from memory bank via memory attention, and $f_{\text{t}-\text{1}}$ denote the features from the last iteration. And the ${\text{U}}_{\text{t}}$ is calculated at each pixel as a function of the current frame image feature $f_{t}$ and retrieved feature $\overset{mem}{\underset{t}{f}}$ using Eq (5):


$$\begin{array}{c} U_{t}=\sigma \left(Conv\left(f_{t},\overset{mem}{\underset{t}{f}}\right)\right) \end{array}$$ (5)

In which, σ denotes the Sigmoid activation function to constrain the uncertainty between 0 and 1, and Conv is a 3×3 convolutional layer that learns to identify regions where features are inconsistent. Furthermore, the uncertainty is learned with loss function formulated as Eq (6):


$$\begin{array}{c} L_{seg}=\sum_{i}\left({\left|M_{i}-\overset{GT}{\underset{i}{M}}\right|}_{\text{1}}\cdot \text{exp}\left(-U_{i}\right)+\frac{\text{1}}{\text{2}}U_{i}\right) \end{array}$$ (6)

Where the $\overset{GT}{\underset{i}{M}}$ is the ground truth mask label for pixel i, $M_{i}$ is predicted mask from model, $\text{exp}\left(-U_{i}\right)$ acts as a learnable weight, if the model prediction is high uncertainty, the penalty decreases for a wrong prediction, and the $\frac{\text{1}}{\text{2}}U_{i}$ is the regularization term.

Specifically, to derive the current features $\overset{mem}{\underset{t}{f}}$ fused from memory bank, the module leverages a scaled L2 similarity metric to retrieve similar features by pixel-wise attention across the keys from memory bank, as formulated in Eq (7).


$$\begin{array}{c} s(k,q{)}_{ij}=-\lambda_{i}\sum_{c}e_{cj}{\left(k_{ci}-q_{cj}\right)}^{\text{2}} \end{array}$$ (7)

In which,$\;\lambda \in [\text{1},\infty {)}^{\text{N}}$ denotes the shrinkage term, which scales the similarity values, $e\in [\text{0},\text{1}{]}^{{\text{C}}^{\text{k}}\times \text{256}}$ represents the selection term, which regulates the relative importance to ensure that more attention is allocated to the more discriminative channels. This enables the algorithm to focus on target-specific region while retaining information of surrounding regions, as elaborated in [20]. Specifically, the function $(\cdot {)}^{\text{2}}+\text{1}$ is applied to the shrinkage factor λ to constrain its range to [1, ∞), and the sigmoid function is used to limit the selection factor e to be in [0, 1].

#### 3.3.2 Motion uncertainty estimation.

Following [4], the Kalman Filter-based motion modeling is employed to resolve association ambiguities. The process can be defined as Eq (8):


$$\begin{array}{c} \begin{array}{c} \begin{array}{c} z_{t}=[x,y,a,h,\;x^{\prime },y^{\prime },\;a^{\prime },\;h^{\prime }]\\ \begin{array}{c} \begin{array}{c} \mathrm{\backslash vspace}1mm{\hat{z}}_{t+\text{1}|t}=A{\hat{z}}_{t}\\ \;{\hat{z}}_{t+\text{1}}={\hat{z}}_{t+\text{1}|t}+K_{t+\text{1}}(z_{t+\text{1}}-H{\hat{z}}_{t+\text{1}|t}) \end{array}\\ \overset{t}{\underset{kf}{s}}=IoU({\hat{z}}_{t+\text{1}},\;M) \end{array} \end{array}\mathrm{\backslash vspace}1mm\\ M^{⋆}=argmax_{M_{i}}\left(\alpha \cdot s_{kf}\left(M_{i}\right)+(\text{1}-\alpha )\cdot s_{mask}\left(M_{i}\right)\right),\;i\in \{\text{1},\text{2},\text{3}\} \end{array} \end{array}$$ (8)

Where z is the state vector, x, y represents the top-left coordinate of the bounding box, a denotes aspect ratio, height h, and their respective velocities. Specifically, A is the state transition matrix to predict the next state based on the current state, and the state is updated via Kalman gain $K_{t+\text{1}}$, which depends on the motion uncertainty $P_{t+\text{1}|t}$, H is the observation matrix to map the state vector the measurement space, here extracting box position x,y from the full state z, and the ${\text{s}}_{\text{mask}}$ denotes the predicted mask score. In this module, assuming the object motion adheres to a linear constant velocity model, and further, and the motion uncertainty is represented by the updated covariance matrix $P_{t+\text{1}}$ with Eq (9):


$$\begin{array}{c} \begin{array}{c} \begin{array}{c} P_{t+\text{1}|t}=AP_{t}A^{T}+Q\\ \;K_{t+\text{1}}=P_{t+\text{1}|t}H^{T}{\left(HP_{t+\text{1}|t}H^{T}+R\right)}^{-\text{1}} \end{array}\mathrm{\backslash vspace}1mm\\ P_{t+\text{1}}=\left(I-K_{t+\text{1}}H\right)P_{t+\text{1}|t} \end{array} \end{array}$$ (9)

In which, $P_{t+\text{1}|t}$ represents the posterior uncertainty from the previous frame, and Q is the process noise, is defined as a diagonal matrix that represents the deviation from the linear const model, and R denotes the measurement noise, representing the covariance of the SAM2 detections. $K_{t+\text{1}}$ is estimated the Kalman Gain, and uncertainty $P_{t+\text{1}}$ is updated based on $K_{t+\text{1}}$. Under the linear model assumption, the position is set to 0.05, and the velocity is 0.00625 in Q, and ratio and its velocity are set to 0.01 and 1e-5, respectively. The measurement noise R from SAM2 model is also diagonal configured to 0.05 to prevent overfitting to a certain mask. Because the initial position, the first prompt, is given, the initial position of the uncertainty matrix P is set to 0.1 and the velocity is 0.0625. If the SAM2 is accurate, the Kalman Gain K increases, the module will pull the state closer to the new observation. While if the motion prediction is very certain, which means P is close to zero, K decrease, the module will ignore the noisy jump of the detections and stick to the predicted trajectory.

Furthermore, the KF-IoU score ${\text{s}}_{\text{kf}}$ is computed by calculating the Intersection over Union (IoU) between the predicted masks from SAM2 and the bounding box derived from the predicted states, as described in the third formula of Eq (8). The predicted mask is selected according the hybrid score of mask score and KF-IoU score ${\text{s}}_{\text{kf}}$, and the updated features can be obtained with Eq (10):


$$\begin{array}{c} f_{t}=\alpha f_{t}⊙\overset{t}{\underset{mask}{s}}+(\text{1}-\alpha )\overset{mem}{\underset{t}{f}}⊙\overset{t}{\underset{kf}{s}} \end{array}$$ (10)

Where α denotes the weight for fusing the enhanced features from appearance and motion estimation modules. Similar to [4], to ensure the robustness of the motion modeling, the motion module is activated if and only if the tracked object is being successfully update in the past $\tau_{kf}$ frames.

#### 3.3.3 Memory management.

To effectively maintaining both fine-grained local temporal dynamics and global semantic consistency, improving the robustness and generalization ability of the model, the long-term memory updating and short-term memory selection mechanism is utilized. Further, in the long-term memory design, the amount of old memory to be forgotten is dynamically determined by a linear attention gating mechanism that relies entirely on input data to automatically compute gating weights, achieving a balance between performance preservation and computational cost reduction.

**Short-term memory selection.** To perform the consistent feature memory propagation of the target, the short-term memory selection mechanism similar to [5] is applied. Specifically, we select ${\text{N}}_{\text{m}}$ memories from the feature candidates based on the set of scoring functions, which composes of $s_{mask},s_{obj},s_{kf}$, as defined in Eq (11):


$$\begin{array}{c} \begin{array}{c} s_{obj}\;=\;I\left(\;\overset{t^{\prime }}{\underset{obj}{s}}>\text{0};t^{\prime }=t-\Delta \right)\\ \;B_{t}=\{m_{i}|I\left(s_{mask}{\;>\delta }_{mask},s_{obj},s_{kf}>\delta_{kf}\right)=\text{1},\;t-N_{m}\leq i<t\} \end{array} \end{array}$$ (11)

where ${\text{N}}_{\text{m}}\;$is the maximum number of historical frames to be traced back. Subsequently, the short-term memory bank ${\text{B}}_{\text{t}}$ is fed into the memory attention layer for feature retrieval. Specifically, following [5], we set the ${\text{N}}_{\text{m}}=\text{3}$ for selected units, others for long-term memory, and the short-term memory is dynamically updating and the same strategy of only being updated when target is present is utilized with Δ = 5. In this manner, the most relevant memory units are retained, which can resist the distractions from crowd background or visual similar objects to avoid introducing invalid or noisy memory information.

**Long-term memory updating.** In SAM 2, the original features are stored in the memory bank, in long videos, the memory storage will grow rapidly and consume more computation. And for dynamic object, the longer features play less importance on current represent. Therefore, the long-term memory updating mechanism is designed to balance the computation and performance, as illustrated in Fig 4. Specifically, the linear attention is applied to compress the features of the historical representation. The standard attention-based memory mechanism accepts an input sequence $x_{t}\in R^{L\times d}$ and outputs the sequence $o_{t}\in R^{L\times d}$ by Eq (12):

**Fig 4:**
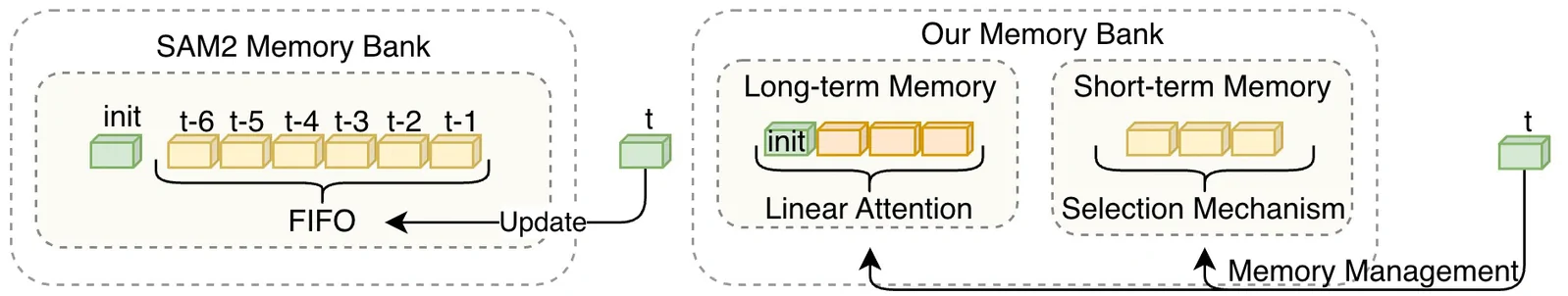
An illustration of the propped memory management module. It splits the memory bank of SAM2 into long-term memory and short-term memory, where the long-term memory is updated with linear attention, and the short-term memory is updated using the proposed mechanism.


$$\begin{array}{c} \begin{array}{c} q_{t},k_{t},v_{t}=x_{t}W_{Q},x_{t}W_{K},x_{t}W_{V}\\ o_{t}=\frac{\sum_{i=\text{1}}^{t}\text{exp}\left(q_{t}\overset{T}{\underset{i}{k}}\right)v_{i}}{\sum_{i=\text{1}}^{t}\text{exp}\left(q_{t}\overset{T}{\underset{i}{k}}\right)} \end{array} \end{array}$$ (12)

In which, L is input length and d is the model hidden dimension, $W_{Q}$, $W_{K}$ and $W_{V}$ represent the query, key and value matrix respectively. Based on the query $q_{t}$, key$\;k_{t}$, and value $v_{t}$ token, the attention is performed which grows with the input length and has quadratic computing complex. To reduce the computing complexity and memory consuming, the Linear attention mechanisms [27] is utilized, which expand the vector hidden state with matrix representation which is calculated with Eq (13):


$$\begin{array}{c} S_{t}=\sum_{i=\text{1}}^{t}\phi {\left(k_{i}\right)}^{T}v_{i} \end{array}$$ (13)

Where ϕ denotes the kernel operation, for linear attention, the linear kernel, setting ϕ to be the identity, without a normalizer is employed in practice [28]. And further the linear attention layer can be described as Eq (14):


$$\begin{array}{c} \begin{array}{c} \begin{array}{c} q_{t}=\frac{q_{t}}{\left||q_{t}|\right|},\;k_{t}=\frac{k_{t}}{\left||k_{t}|\right|}\mathrm{\backslash vspace}1mm\\ S_{t}=S_{t-\text{1}}+\overset{T}{\underset{t}{k}}v_{t} \end{array}\\ o_{t}=q_{t}S_{t} \end{array} \end{array}$$ (14)

In which, the query and key vectors are normalized before the attention calculation as shown in Eq (14). It can be observed that the linear attention layer is essentially a recurrent layer with matrix-valued hidden states $S_{t}\in R^{{\text{d}}_{\text{k}}\times {\text{d}}_{\text{v}}}$, that is updated via the outer-product $\overset{\text{T}}{\underset{\text{t}}{k}}{\text{v}}_{\text{t}}$. Moreover, to dynamically updated the states automatically, the data-dependent and learnable 2D forget gate ${\text{G}}_{\text{t}}\in (\text{0},\text{1}{)}^{{\text{d}}_{\text{k}}\times {\text{d}}_{\text{v}}}$ is further applied, as shown in Eq (15):


$$\begin{array}{c} {\text{S}}_{\text{t}}={\text{G}}_{\text{t}}⊙{\text{S}}_{\text{t}-\text{1}}+\overset{⊤}{\underset{\text{t}}{k}}v_{\text{t}} \end{array}$$ (15)

where ⊙ denotes the Hadamard product. Follow [28], to make the learnable more stable, the parameter-efficient parameterization is applied base on the outer-product-based low-rank approximation: $G_{t}=\overset{T}{\underset{t}{\alpha }}\;\beta_{t}$, which has $d\cdot (d_{v}+d_{k})$ parameters versus $d_{v}\cdot d_{k}$ parameters of original matrix form. And the $\alpha_{\text{t}}$ and $\beta_{t}$ are data-dependent to balance the short-term and long-term memory, which are calculated via a linear projection of the input $x_{t}$ with Eq (16):


$$\begin{array}{c} \begin{array}{c} \alpha_{\text{t}}=\sigma (x_{t}W_{\alpha })\\ \beta_{t}=\sigma (x_{t}W_{\beta }) \end{array} \end{array}$$ (16)

In which, $W_{\alpha }\in R^{d\times d_{k}}$ and $W_{\beta }\in R^{d\times d_{v}}$, and σ is the sigmoid function. This gating updating mechanism determines how much the new information from the short-term memory is written into the memory bank of the SAM2 model, and how much the old state of the long-term memory is preserved.

#### 3.3.4 Loss function.

For frame t, let $M_{t}$ be the predicted segmentation mask, and $\overset{GT}{\underset{t}{M}}$ be the corresponding ground-truth. To optimize the proposed model, we adopt the common loss functions widely used in video object segmentation (VOS) tasks [8,12,46], as formulated in Eq (17):


$$\begin{array}{c} \begin{array}{c} L_{ce}=\overset{GT}{\underset{t}{M}}\left(-\text{log}\left(M_{t}\right)\right)+\left(\text{1}-\overset{GT}{\underset{t}{M}}\right)\left(-\text{log}\left(\text{1}-M_{t}\right)\right)\\ L_{dice}=\text{1}-\frac{\text{2}M_{t}\overset{GT}{\underset{t}{M}}+\text{1}}{M_{t}\overset{GT}{\underset{t}{+M}}+\text{1}} \end{array} \end{array}$$ (17)

And the overall loss function is summarized as Eq (18):


$$\begin{array}{c} L=\lambda_{ce}\;L_{ce}+\lambda_{dice}\;L_{dice}+\lambda_{seg}\;L_{seg} \end{array}$$ (18)

where $\lambda_{\text{ce}}$ and $\lambda_{\text{dice}}$ are the coefficients for the cross-entropy and dice losses, respectively.

This whole procedure is illustrated in Fig 3. Starting from the initial prompt, UAMP propagates the consistent features across the entire video with the appearance and motion uncertainty estimation. The proposed pseudo-label enhancement algorithm is summarized in Algorithm 1.

Algorithm 1. Proposed UAMP Method.

Input: video frames $\left\{I_{\text{1}},I_{\text{2}},\cdots ,I_{N}\right\}$, initial prompt $T_{\text{0}}$

Output: masks $\left\{M_{\text{1}},M_{\text{2}},\cdots ,M_{N}\right\}$

1: Initialize memory bank $\overset{i}{\underset{mem}{f}},\;i\in \{\text{1},\text{2}\cdots ,\text{7}\}$ and long-term memory $\overset{i}{\underset{mem}{S}}$

2: Initialize motion state $z_{\text{0}}$ from $T_{\text{0}}$, covariance $P_{\text{0}}$ and the object token $E_{token}$

3: for t = 1 to N do

4: $f_{t},E_{token},\overset{t}{\underset{mask}{s}},\overset{t}{\underset{obj}{s}},M_{t}\leftarrow SAM\text{2}(I_{t},\;T_{t})$ ⊳ get image feature, prompt embedding, mask score, object score and masks.

5: if t > 1 then

6:  Query current features $\overset{m}{\underset{t}{f}}$ from memory bank $\overset{i}{\underset{mem}{f}}$ based on the input image feature: $\overset{m}{\underset{t}{f}}=\sum_{i}s\left(f_{t},\;\overset{i}{\underset{mem}{f}}\right)\cdot \overset{i}{\underset{mem}{f}}$ using Eq (7)

7:  # The appearance uncertainty prediction

8:  Predict the appearance uncertainty $U_{t}$ using Eq (5)

9:  Update feature $f_{t}$ using Eq (4) ⊳ appearance-uncertainty based updating

10:  # The motion uncertainty estimation

11:  calculate the state $z_{t}$ based on $z_{\text{0}}$, and the posterior uncertainty $P_{t|t-\text{1}}$ based on $P_{\text{0}}$ using Eq (8)

12:  Estimate the motion uncertainty $P_{t}$ using Eq (9) and get the motion score $s_{kf}$

13:  Get feature $f_{t}$ using Eq (10) ⊳ motion-uncertainty based updating

14:  # memory management

15:  Update short-term memory $\overset{i}{\underset{mem}{f}}$ using Eq (11) ⊳ short-term memory selectin based on the $s_{mask},s_{obj}$ and $s_{kf}$

16:  Calculate the gating $\alpha_{t},\beta_{t}$ using Eq (16)

17:  Update long-term memory $S_{t}$ using Eq (15) ⊳ long-term memory updating

18:  # parameter optimizations

19:  Calculate the appearance uncertainty loss $L_{seg}$ using Eq (6) and segmentation loss L using Eq (18)

20:  Optimize the model parameters using AdamW optimizer

21: else

22:  put $f_{t}$ to memory bank $\overset{i}{\underset{mem}{f}}$ and $\overset{i}{\underset{mem}{S}}$

23: end if

24: end for

## 4 Experiments and results

### 4.1 Experiments

#### 4.1.1 Dataset.

To comprehensively evaluate the effectiveness of our proposed method, we conduct extensive experiments on five VOS benchmarks. The specific evaluation setup is to systematically increase the difficulty of VOS tasks by targeting core challenging scenarios in video sequences, including occlusions, long videos, extreme transformations, object diversity, and scene diversity. For quantitative assessment, the following three metrics are employed: J for region similarity to measure the accuracy of region-level segmentation, F for contour accuracy to capture the fine-grained target boundaries, and the combined J&F for harmonic mean of J and F [1]. All evaluations are conducted in a semi-supervised setting, and the detailed information about datasets is presented as follows:

DAVIS 2017 [2] is a multi-object benchmark extended on DAVIS 2016, where the test split contains 30 more challenging videos with 89 objects. YouTube-VOS [29] is a large-scale diverse VOS benchmark, containing 3471 videos in the training split with 65 categories, additional 26 unseen categories are given.

MOSE [19] is a challenging VOS dataset focused on complex, real-world scenarios, and 2,149 video clips with 431,725 segmentation masks are provided. LVOS v1 [30] is collected as the long-term video object segmentation in realistic scenarios. It contains 720 video clips with 296,401 frames and 407,945 annotations, and the average video duration is over 60 seconds. LVOS v2 [18] expends LVOS v1 and convers 44 categories. Specifically, 12 of these categories are designated as unseen classes to evaluate the generalization capabilities of VOS models. SA-V [1] comprises 50.9K video clips, 642.6K masklets with 35.5M annotated masks, which is a large-scale video segmentation dataset for VOS. The key challenge lies in the presence of small, occluded, and reappearing objects in the video sequences.

#### 4.1.2 Model details.

For image encoder, the input image is first scaled to the resolution of 1024, and the image embeddings are obtained by fusing the stride 16 and 32 features from Stages 3 and 4 of the Hiera [26] image encoder with the dimension 448×64×64 and 896×32×32 respectively. These multi-scale features are then fused via a pyramid network following [1], and model dimension of Hiera is 256. The positional embeddings are calculated with the windowed absolute positional embeddings, and the positional information spanning across windows is obtained by interpolating the positional embeddings. For memory attention, the input feature map is the 256×64×64 embeddings generated by the memory encoder, the 2d spatial Rotary Positional Embedding (RoPE) [31] with the dimension of 256 is utilized in both self-attention and cross-attention layers, and this module composes 4 layers. For mask decoder, the stride 4 and 8 features from Stages 1 and 2 are added to the up-sampling layers to get high-resolution features. Following [7], the appearance uncertainty prediction module comprises one 1 × 1 convolution and two 3 × 3 convolutions. It takes the input of a concatenation of current frame feature, last frame feature, and last mask prediction, and outputs a one-channel uncertainty mask of the query token. For memory bank, the memory features 256×64×64 are projected to the dimension of 2048 for 8 memory banks via a linear layer, and the short-term memory selection is designed follows DAM [5] as discussed in Sect 3.3. The long-term memory updating composes 2 layers of the transformer-like structure, in which the self-attention layer is replaced with the linear-attention layer. And the matrix state is projected to the same dimension as short-term memory with a linear projection layer.

#### 4.1.2 Train details.

During training, the backbone of the SAM 2 model is frozen and one Nvidia A100 40G GPU is used. To boost the segmentation quality on long videos, the proposed method is fine-tuned where the 16-frame sequences are sampled on videos from LVOS, MOSE and YouTube-VOS training datasets, which is different from the 8-frame sequences of the SAM 2 training strategy. Specifically, The SA-V dataset is composed of the middle 20 videos from sav_000491 to sav_000510, the first 300 videos are extracted from the MOSE dataset according to the meta_train.json file, and the last 50 videos are chosen from the LVOS v1 dataset and last 150 videos from the LVOS v2 dataset according to the meta.json. Following [1], only the mask with the lowest segmentation loss is taken as a supervision, none of the mask outputs is supervised if there are no masks in current frame. For optimization, the AdamW method is employed. The learning rate is set to 1e-5 with a weight decay of 0.05, and the momentum is set to 0.9 and 0.998. the main training process runs for 60K iterations with batch size 16, and we drop the learning rate by a factor of 2 after first 20K iteration. And Table 1 further details the hyperparameter settings for training.

**Table 1:** The training hyperparameter settings of the proposed methods.

config	value
optimizer	AdamW
optimizer momentum	$\beta_{\text{1}},\beta_{\text{2}}=\text{0}.\text{9},\;\text{0}.\text{998}$
weight decay	0.05
learning rate (lr)	1.0e-5
lr schedule	cosine
warmup	linear, 20k iters
batch size	16
short-term memory queue	3×2048
long-term memory state $S_{t}$	32×32
memory bank	7×2048
precision	Bfloat16
linear attention layer	2
image augmentation	hflip, colorjitter(0.1), grayscale(0.05)
image normalization	[0.485,0.456,0.406] (mean) [0.229,0.224,0.225] (std)
mask losses (weight)	focal(20), dice(1)

For inference, due to the recurrent refinement characteristic of the memory-based paradigm, the first-frame warm-up strategy is adopted. Specifically, the first frame with prompts is repeated 3 times, with each repetition treated as the initial frame to initialize the memory bank, to enhance robustness against the given prompts.

### 4.2 Results

#### 4.2.1 Quantity results.

Semi-supervised video object segmentation. We evaluate the proposed UAMP with the semi-supervised video object segmentation (VOS) setting. In which, we compare our proposed method, UAMP, with various state-of-the-art VOS methods on both the SA-V [1] and MOSE [19] datasets, as shown in Table 2. Specifically, our method achieves a J &F score of 81.6 on the SA-V validation dataset, a 1.9-point improvement over SAM2Long, 81.7 on SA-V testing dataset, and 77.1 on MOSE validation dataset.

**Table 2:** Performance comparison with the state-of-the-arts methods on SA-V and MOSE datasets.

Method	SA-V val			SA-V test			MOSE val		
	J&F	J	F	J&F	J	F	J&F	J	F
STCN [32]	61.0	57.4	64.5	62.5	59.0	66.0	52.5	48.5	56.6
SwinB-AOT-L [30]	51.1	46.4	55.7	50.3	46.0	54.6	59.4	55.5	63.2
SwinB-DeAOT-L[33]	61.4	56.6	66.2	61.8	57.2	66.3	59.9	55.7	64.0
RDE [34]	51.8	48.4	55.2	53.9	50.5	57.3	46.8	42.4	51.3
XMem [20]	60.1	56.3	63.9	62.3	58.9	65.8	59.6	55.4	63.7
DEVA [35]	55.4	51.5	59.2	56.2	52.4	60.1	61.8	66.0	70.3
Cutie-base++ [22]	61.3	58.3	64.4	62.8	59.8	65.8	71.7	67.6	75.8
LiVOS [36]	–	–	–	–	–	–	64.8	60.8	68.7
SAM2 [1]	76.1	72.9	79.3	77.0	73.5	79.8	76.6	72.6	80.6
SAM2Long [3]	79.7	74.7	84.7	80.8	76.8	84.7	75.2	–	–
SAM2.1 [1]	78.6	75.1	82.0	79.6	76.1	83.2	74.5	–	–
SAM2.1Long [3]	81.1	77.5	84.7	81.2	77.6	84.9	75.2	–	–
Ours	81.6	77.9	85.3	81.7	78.0	85.4	77.1	73.1	81.1

To evaluate the performance of proposed UAMP in unseen categories, we also compare our method with various state-of-the-art VOS methods on both LVOS 1 [37] and LVOS 2 [18] datasets, which challenges models with difficult visual cues, as shown in Table 3. It can be seen that, UAMP attains J &F scores of 83.7 and 86.2 on the v1 and v2 subsets, respectively, outperforming SAM2Long by 2.4 and 2.0 points. Notably, UAMP particularly excels in unseen categories, achieving J and F scores of 83.2 and 87.5. The significant improvements of 4.1 and 3.3 points over SAM2Long highlight its robust generalization capabilities. These results showcase the effectiveness of the proposed consistent memory propagation in long-time video segmentation scenarios.

**Table 3:** Performance comparison with the state-of-the-arts methods on LVOS datasets. Subscript s and u denote scores in seen and unseen categories.

Method	LVOS1 val			LVOS2 val				
	J&F	J	F	J&F	Js	Fs	Ju	Fu
STCN [32]	48.9	43.9	54.0	60.6	57.2	64.0	57.5	56.2
CFBI [13]	51.5	46.2	56.7	55.0	52.9	59.2	51.7	56.2
SwinB-DeAOT-L [33]	–	–	–	63.9	61.5	69.0	58.4	66.6
RDE [34]	53.7	48.3	59.2	62.2	56.7	64.1	60.8	67.2
XMem [20]	52.9	48.1	57.7	64.5	62.6	69.1	60.6	65.6
DDMemory [37]	60.7	55.0	66.3	–	–	–	–	–
Cutie-base++ [22]	66.0	61.3	70.6	–	–	–	–	–
LiVOS [36]	51.2	47.3	55.1	–	–	–	–	–
SAM2 [1]	77.9	73.1	82.7	79.8	80.8	86.6	71.6	81.1
SAM2Long [3]	81.3	76.4	86.2	84.2	82.3	89.2	79.1	86.2
SAM2.1 [1]	80.2	75.4	84.9	84.1	80.7	87.4	80.6	87.7
SAM2.1Long [3]	83.4	78.4	88.5	85.9	81.7	88.6	83.0	90.5
Ours	83.7	78.8	88.6	86.2	81.9	90.5	83.2	89.5

To further evaluate the effectiveness of UAMP, we also apply it to three widely used object tracking benchmarks, namely LaSOT [38], LaSOText [39], and GOT-10k [40]. We first generate an initial mask from the bounding box provided for the first frame using the SAM2 Mask decoder. Subsequently, all subsequent masks are transformed into bounding box format by identifying the minimum and maximum coordinates of their non-zero indices. For comparison, we include recent state-of-the-art methods specifically designed for VOT, including SAMURAI [4] and DAM2SAM [5].

Based on the strong foundation of SAM2, we achieve competitive performance with state-of-the-art VOT methods, as shown in Table 4. Specifically, our method, UAMP, delivers results that are comparable to the performance of SAMURAI and DAM2SAM, two recent methods specifically optimized for VOT. Where, UAMP’s uncertainty-aware feature fusion and linear attention design achieves a more balanced adaptation to VOT’s core demands—robustness to drift, precision in localization, and efficiency in long-sequence processing, validating the effectiveness and rationality of our proposed UAMP framework.

**Table 4:** State-of-the-art comparison on three standard bounding box benchmarks. The results are reported in terms of AUC (Area Under the success rate Curve) for LaSoT and LaSoText, and AO (Average Overlap) for GOT-10k.

Method	LaSoT (AUC)	LaSoText (AUC)	GoT10k (AO)
KeepTrack [41]	67.1	48.2	–
DropTrack [42]	71.8	52.7	75.9
SeqTrack [43]	72.5	50.7	74.8
ROMTrack [44]	71.4	51.3	74.2
ODTrack [45]	74.0	53.9	78.2
LORAT [46]	75.1	56.6	78.2
SAM2 [1]	70.0	56.9	80.7
SAM2Long [3]	73.9	60.9	81.1
SAMURAI [4]	74.2	61.0	81.7
DAM2SAM [5]	75.1	60.9	81.1
Ours	75.3	60.9	81.5

#### 4.2.2 Ablation study.

**Effect of the individual modules**. We demonstrate the effect of the with or without appearance uncertainty prediction, motion uncertainty estimation and long-term memory updating on different settings in Table 5. Both of the proposed modules have a positive impact on the SAM 2 model, while combining both can achieve the best J&F on the SA-V, MOSE, LVOSv1 and LVOSv2 datasets with the score of 81.6%, 77.1%, 83.7% and 86.2%, respectively. For these datasets with its inclusion of small, occluded, and reappearing objects, the proposed modules showed the robust performance. Specifically, for the long sequences, such as LVOS datasets, the long-term memory updating design played an important role, which is more validated in subsequent qualitative results.

**Table 5:** Ablations on individual design. The performance is evaluated on SA-V, MOSE, LVOSv1 and LVOSv2 datasets with J&F metric.

Appearance	Motion	Long-term Memory	J&F			
			SA-V val	MOSE	LVOSv1 val	LVOSv2
			76.1	76.6	77.9	79.8
✓			76.3	76.8	78.6	80.7
✓	✓		78.2	77.0	80.3	82.3
✓	✓	✓	81.6	77.1	83.7	86.2

**Effect of the uncertainty weight**. We showcase the effect of the motion uncertainty estimation, where the weight is utilized to fuse the features from memory and appearance uncertainty enhanced features in Table 6. It can be seen that the trade-off between motion and appearance demonstrates a significant impact on tracking and segmentation performance. Our experiments reveal that setting the weight to 0.24 yields the best J&F score on the MOSE, LVOSv1, LVOSv2 dataset, where motion information is needed in the occlusion and reappearing scenes. For further analysis,we give the attention map visualization, predicted object score and motion score in Fig 5. It shows that the estimated motion score has larger variance than visual object score, which also needs the guidance of visual features.

**Table 6:** Effect of the uncertainty weight. The larger α is, the more motion information is contained.

α	J&F		
	MOSE	LVOSv1	LVOSv2
0.00	76.6	77.9	79.8
0.12	76.8	78.2	81.2
0.24	77.1	83.7	86.2
0.48	76.6	77.6	78.9

**Fig 5:**
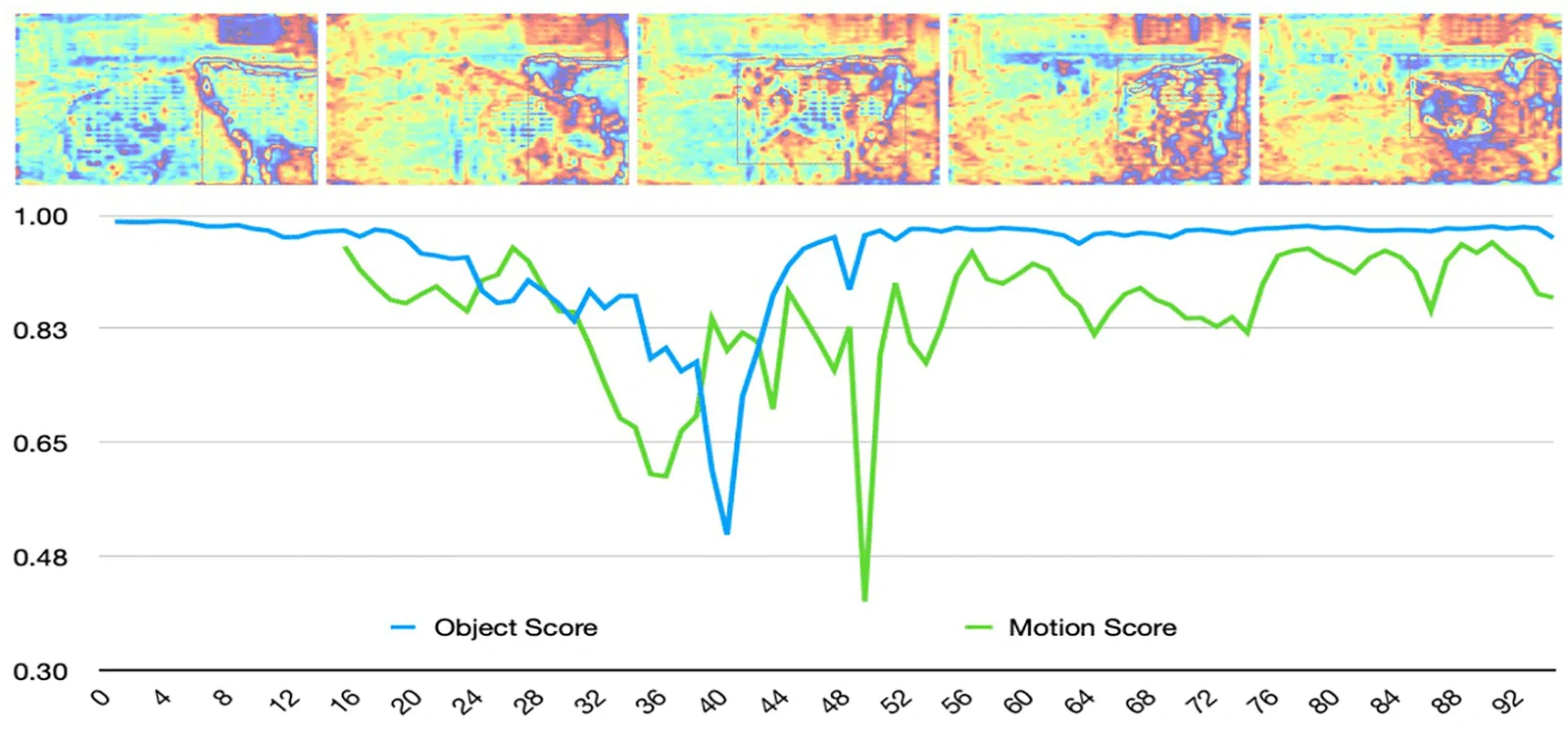
An illustration of the visual object score and estimated motion score from a sample of the MOSE dataset. The first row is the visual attention and the second row is the predicted object score and motion score, where the x axis is the frame index, y axis is the score value.

**Effect of the long-term memory updating**. We investigate the effectiveness of feeding the memory features to a linear attention before adding them to the memory bank. In this ablation, we evaluate on LVOSv1 and LVOSv2 as the benchmark for long-term object segmentation. While prior works have commonly employed GRU states as a means of incorporating memory into the tracking process, our findings in Table 7 suggest that the GRU does not provide a small improvement. Instead, we find the proposed module achieves a comparable performance.

**Table 7:** Effect of the long-term memory updating. Two different updating mechanism is compared with GRU and linear attention.

Type	J&F		
	MOSE	LVOSv1	LVOSv2
–	73.1	76.9	77.0
GRU [1]	73.3	74.5	78.9
Linear Att. [27]	77.1	83.7	86.2

**Effect of IoU threshold for short-term memory selection**. The choice of the IoU threshold $\delta_{kf}$ is crucial for selecting frames with reliable object cues. As shown in Table 8, setting $\delta_{kf}=\text{0}.\text{3}\text{6}$ yields the highest J&F, indicating an effective trade-off between filtering out poor-quality frames and retaining valuable segmentation information.

**Table 8:** Effect of IoU threshold for short-term memory selection. The higher the threshold is, the more reliability the object has.

$\delta_{kf}$	J&F		
	MOSE	LVOSv1	LVOSv2
0	76.3	82.2	84.9
0.36	77.1	83.7	86.2
0.72	76.4	83.3	85.3
0.88	74.1	80.4	82.7

**Runtime analysis**. The incorporation of the uncertainty modeling and an enhanced memory management into the SAM2 segmentation framework introduces minimal computational overhead. Specifically, the appearance uncertainty is composed of merely three convolution layers as shown in Eq (5) and Eq (6), and the motion uncertainty is estimated with 7-dimensional matrix using Kalman filter shown in Eq (8) and Eq (9). Further, the short-term selection maintains a queue with $N_{m}=\text{3}$, and is updated by specific strategy shown in Eq (3). Therefore, the memory and computation of these operations is almost negligible. The main complexity comes from the long-term memory updating module, which composes a 2-laryer transformer block with linear attention, and the training parameters are about 3.51M, 0.93G FLOPs, and with 7.37MB memory consumption. Compared to the SAM2 model with 80.8M parameters, 96.1GFLOPs computation, the memory footprint and inference latency of the proposed method remain almost consistent, as shown in Table 9.

**Table 9:** Runtime analysis on A100. w/indicates with the module, the uncertainty module consists of the appearance and motion uncertainty, and the memory management includes the long-term memory updating and short-term memory selection.

Module	Params (M)	FLOPs (G)	Latency (ms)
SAM2 (baseline)	80.8	96.1G	–
w/uncertainty module	< 0.1	< 0.1	< 0.1
w/memory management	3.51	0.93	< 0.1

#### 4.2.3 Qualitative result.

We present the qualitative results on DAVIS 2017, MOSE, YouTube-VOS, LVOS1, and LVOS2 datasets, as shown in Figs 6, 7, 8, 9, and 10. UAMP demonstrates a significant consistency in long-video object segmentation, maintaining more accurate and consistent tracking of objects across various frames. And it achieves an adaptive balance between short-term and long-term memory for long videos and crowded scenes via structurally distinct yet mutually complementary designs.

**Fig 6:**
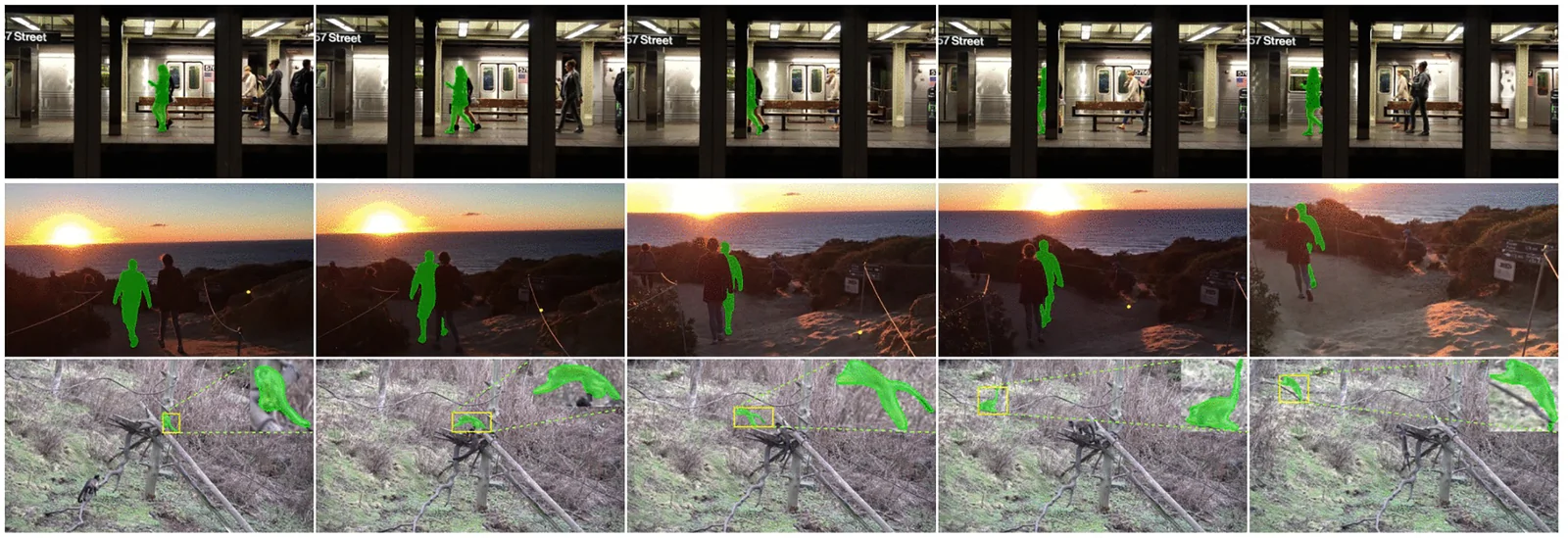
Qualitative results on DAVIS 2017 validation set. Where the tracked objects are colored with green.

**Fig 7:**
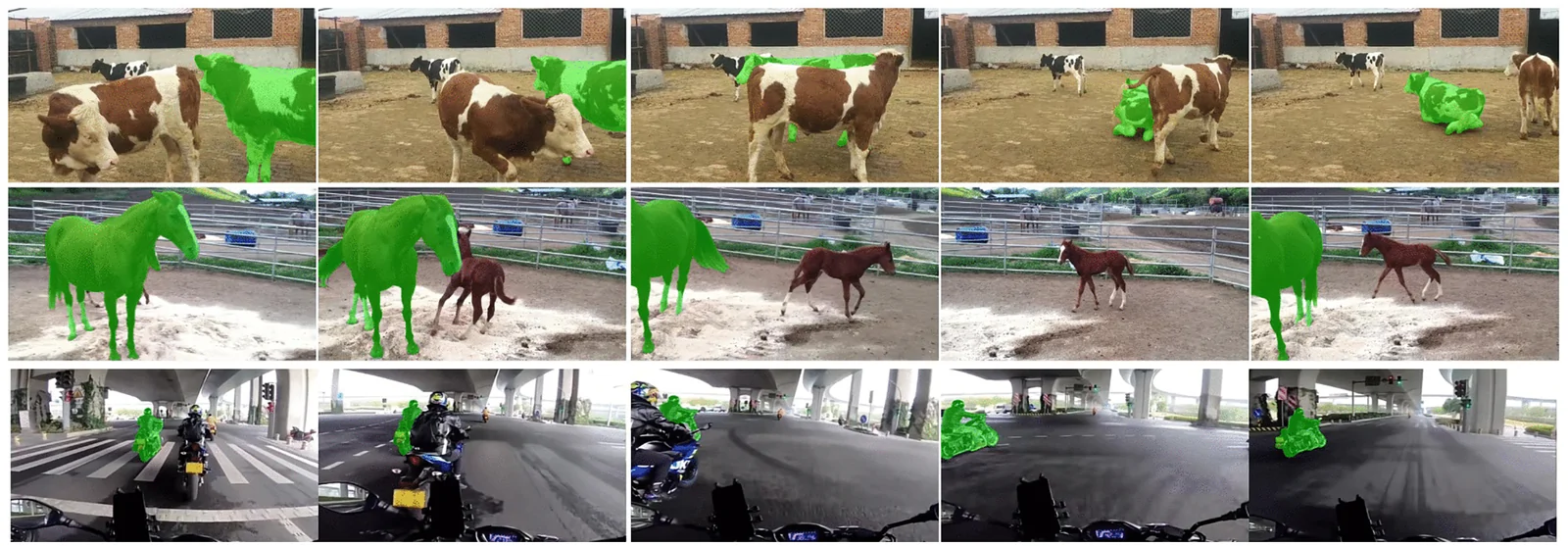
Qualitative results on MOSE validation set. Where multiple similar objects are in the vicinity of the tracked target.

**Fig 8:**
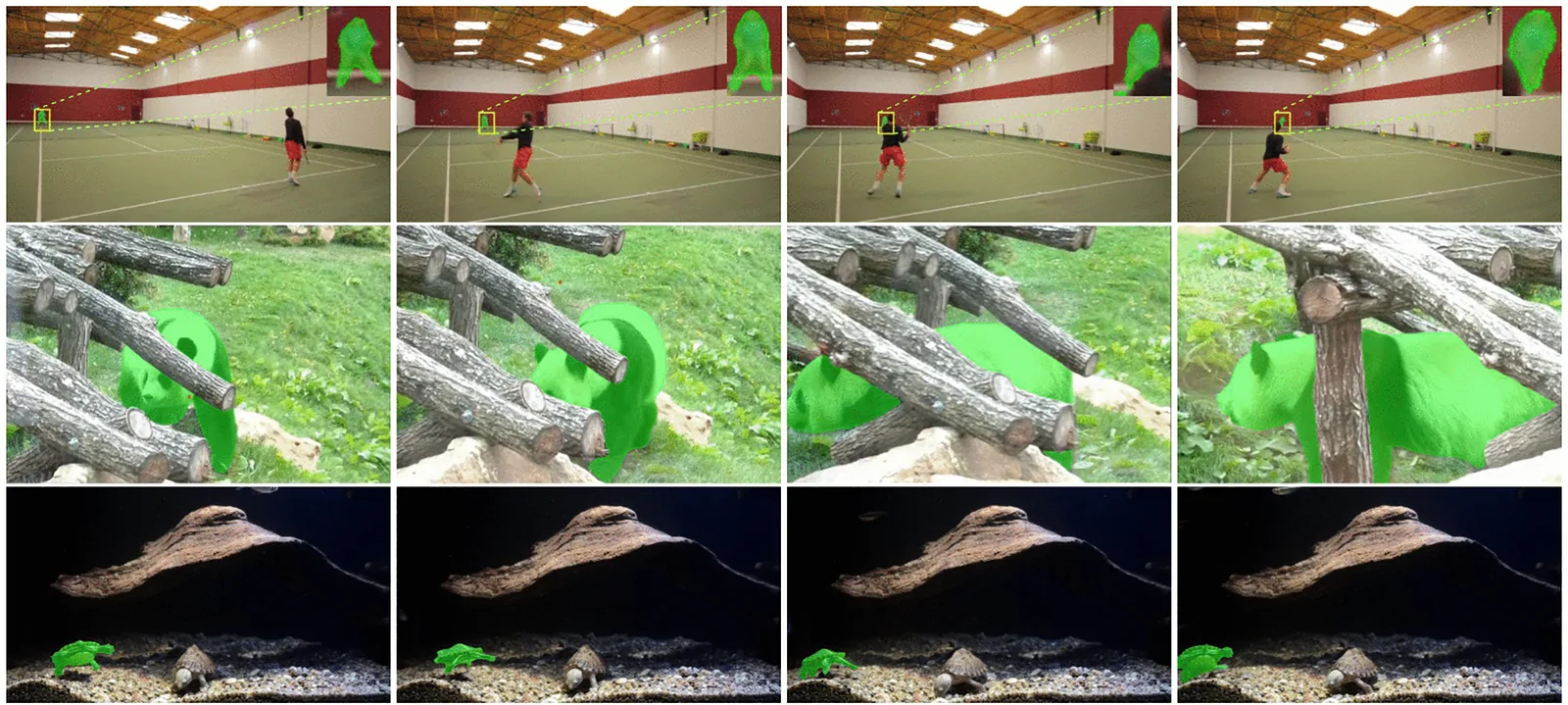
Qualitative results on YouTube-VOS validation set. In which the tracked targets have large shape transformations.

**Fig 9:**
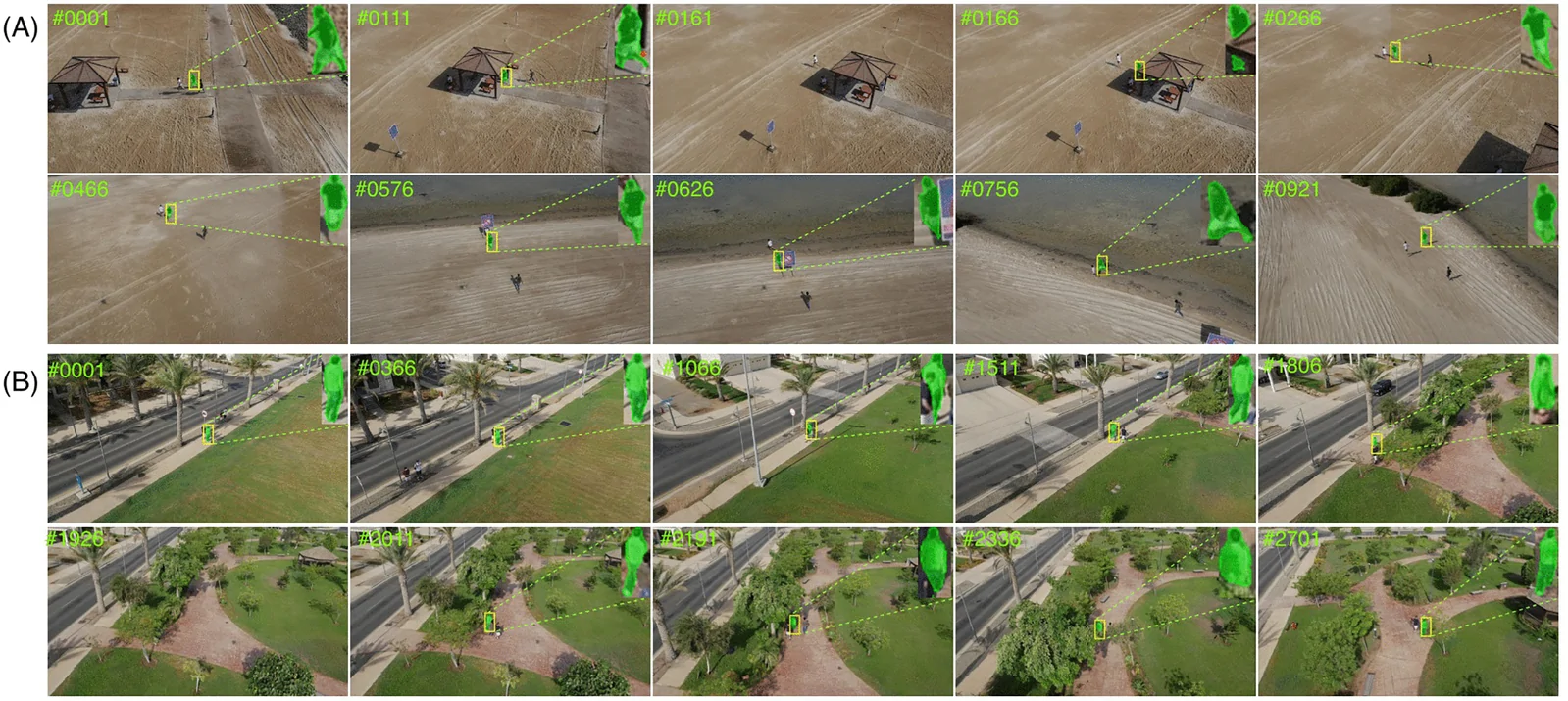
Qualitative results on LVOS v1 validation set. (A) The sample video sequence contains 2681 frames, and the tracked target is far away and completed occluded in the process. (B) A video sequence contains 5526 frames, and the tracked target is distracted by similar objects and completed occluded in the process.

**Fig 10:**
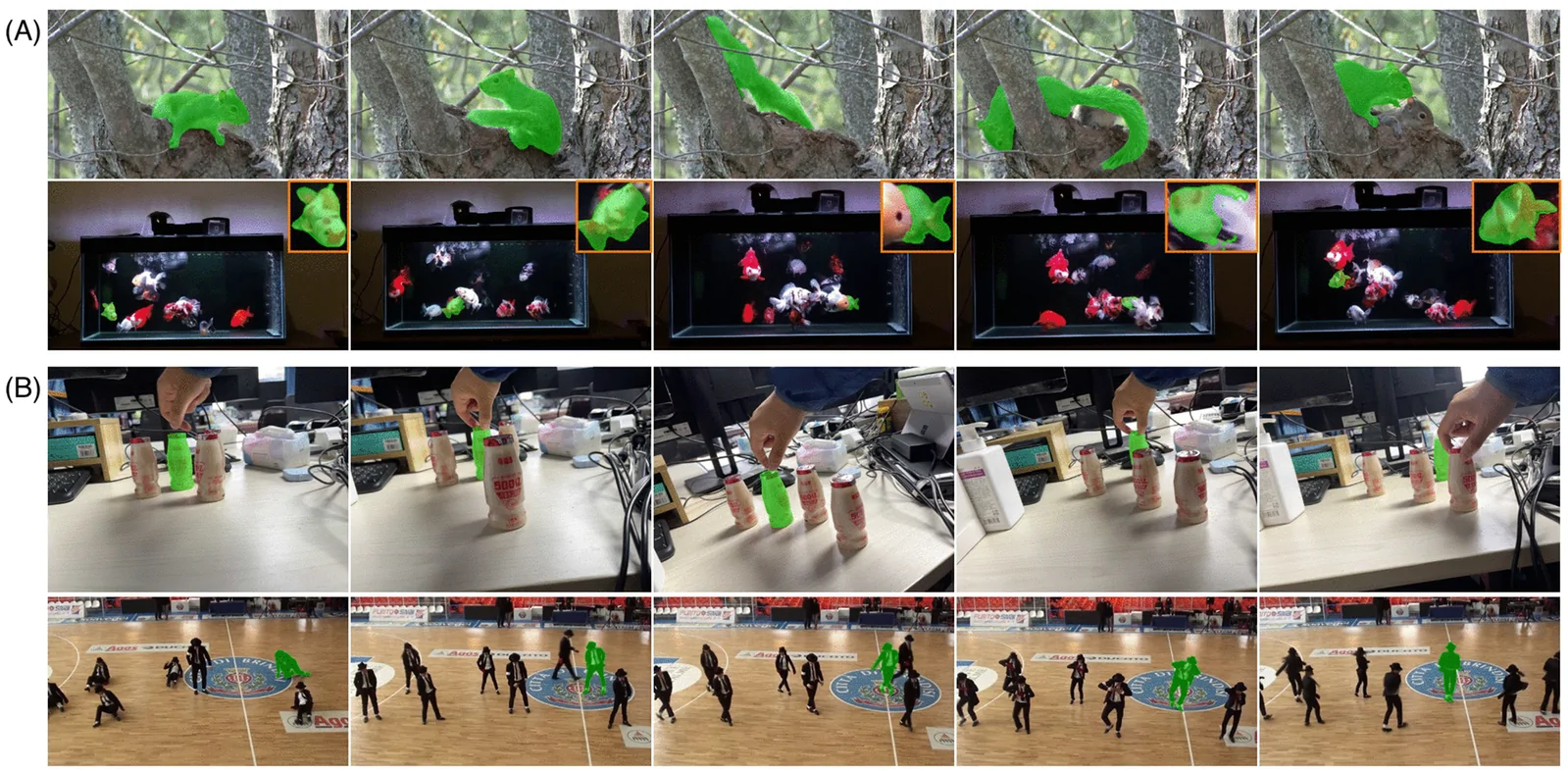
Qualitative results on LVOS v2 validation set. (A) The tracked target has large shape transformation and heavy occlusion. (B) The tracked target is surrounded and occluded by the objects with the similar appearance in the video sequence.

Fig 6 presents the segmentation results on DAVIS 2017 validation video sequences, demonstrating its enhanced ability to handle subtle and ambiguous segmentation tasks. In the first two rows, a person is tracked and segmented with another person around. When the person is partially occluded, by taking advantages of the motion uncertainty estimation and short-term memory, the proposed UAMP still could track part of the target during occlusion and stays on until the end of the sequence. In which, the persons are moving in different direction. The same as the third example, where the monkey far away can be consistently segmented.

More results on MOSE dataset and YouTube-VOS dataset are performed, as shown in Fig 7 and Fig 8 respectively, which involves complex real-world scenes. However, YouTube-VOS, with an average duration of only 4.5 seconds, offers limited opportunities to showcase long-term tracking capabilities. But these results illustrate that we have preserved the fundamental segmentation capabilities of SAM 2, demonstrating the robustness and versatility of UAMP across a range of VOS benchmarks. Particularly, the tracked targets are surrounded by similar objects and have shape transformations, which need the appearance information, motion clues and memory information to be tracked.

To demonstrate the long-term abilities of the proposed method, some results on LVOS 1 dataset are given in Fig 9. The UAMP enhances the SAM2 to handle subtle and ambiguous segmentation tasks. Specifically, In Fig 9(A) the video sequence has 2681 frames, the person is completed occluded and disappeared, and in the 576-th frame the person is occluded again. And further in Fig 9(B) the video length is 5526, and in 1926-th frame, the target is disappeared for about 105 frames, and the proposed method with consistent memory propagation enables it to successfully re-track the correct target, which further validates the long-term memory module.

Furthermore, more segmentation results are demonstrated in Fig 10, where the objects with identical or same appearance surrounds the tracked targets. Specifically, in these situations, the large ambiguity is introduced, challenging the tracking and segmenting. For example, in the second sequence of Fig 10(A), the tracked fish has large shape transformation and occluded by the other fishes with the similar shape. The same as the first sequence of Fig 10(B), the bottle is moved in arbitrary direction, where the consistent memory propagation is needed to handle the appearance changes, movement and dependence on history features.

## 5 Discussion

Despite the consistent performance improvements achieved by UAMP over state-of-the-art methods including SAM2, the proposed framework still faces challenges in modeling fine-grained object structures. As demonstrated in Fig 11, UAMP occasionally fails to preserve precise edge details when handling objects with thin structures, subtle boundaries, leading to degradations in boundary accuracy. This limitation arises partly because generic segmentation-oriented training objectives and memory-based propagation mechanisms tend to prioritize overall target localization over high-fidelity boundary preservation.

**Fig 11:**
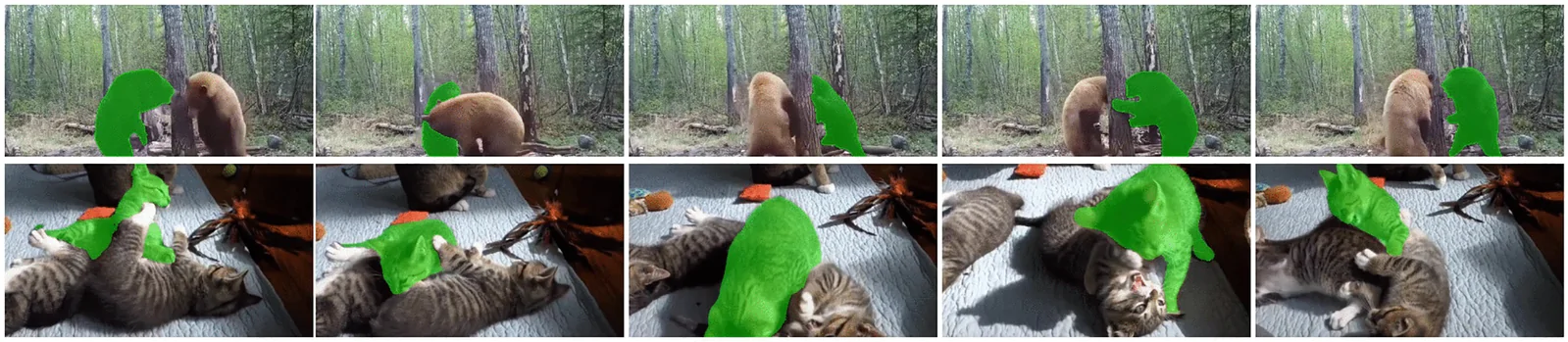
Some examples on MOSE dataset. The targets have very subtle boundaries, which need more detailed features or annotation as supervision.

To mitigate this issue and further enhance segmentation precision, several promising directions can be explored in future research. First, integrating high-resolution structural encoders or advanced feature refinement modules, such as the strategy adopted in HQ-SAM, could help the model capture richer spatial details and sharper boundary representations. Second, extending the training scheme with auxiliary supervision from matting datasets [7], which provide explicit and fine-grained boundary annotations, would enable the model to better distinguish subtle structural variations and improve edge consistency during temporal propagation.

## 6 Conclusions

In this paper, we propose UAMP, an enhanced variant of the SAM 2, specifically designed to address the inherent limitations of SAM 2—error accumulation, poor occlusion robustness, and inadequate adaptation to dynamic object changes—in long-term video object segmentation (VOS) tasks. The core of UAMP lies in its proposed uncertainty-aware memory propagation framework, which integrates consistent memory propagation with three mutually complementary components: appearance uncertainty prediction, motion uncertainty estimation, and a dual memory management mechanism (long-term memory updating & short-term memory selection). Unlike the original SAM 2, UAMP leverages uncertainty modeling to dynamically adjust memory propagation and selection, enabling it to effectively mitigate error accumulation while adaptively accommodating the dynamic changes of objects over extended video durations, thereby enhancing the consistency and reliability of segmentation results. Extensive quantitative and qualitative evaluations on five mainstream VOS benchmark datasets validate the effectiveness of UAMP, and the proposed uncertainty-aware memory propagation framework provides a novel technical approach for optimizing memory-based video segmentation models, offering a reusable paradigm for future research on dynamic object tracking and segmentation. Despite the promising results, our method still fails to resolve precise boundary information in some challenging scenarios. For the future work, high-quality feature extraction paradigms like HQ-SAM or fine-tuned on matting datasets [7] can be integrated.
