# Supplementary material for: UAMP: Consistent video object segmentation with uncertainty-aware memory propagation
Source: PLoS One. 2026 Jul 7;21(7):e0353156. doi: 10.1371/journal.pone.0353156 (PMC13718450; doi:10.1371/journal.pone.0353156)
Supplement: Supplementary file 1 [file pone.0353156.s002.docx]

**For academic editor:**

1. Please ensure that your manuscript meets PLOS ONE's style requirements, including those for file naming.

**Response**: We thank the editor for the reminder. We have carefully checked and revised our manuscript to ensure full compliance with PLOS ONE’s style and formatting requirements, including the file naming conventions and all other journal specifications. All necessary adjustments have been made accordingly.

2. Please note that PLOS One has specific guidelines on code sharing for submissions in which author-generated code underpins the findings in the manuscript.

**Response**: We agree with the suggestion on making the code available. At present, we provided an initial version of the code for review and verification purpose, with the url: <https://github.com/Feynluo/uamp-sam2> . We will further organize, refine, and fully release the complete code on a public repository upon formal acceptance of the manuscript.

3. Thank you for stating the following financial disclosure: This research was funded by Shaanxi University of Technology, grant number SLGRC202416. Please state what role the funders took in the study.

**Response**: The funder (Shaanxi University of Technology, grant number SLGRC202416) provided financial support only for this study. The funder had no role in the design of the study, data collection, data analysis, interpretation of results, writing of the manuscript, or decision to publish. And the role of funder statement is amended in the cover letter.

4. Thank you for stating the following in the Acknowledgments Section of your manuscript: This research was funded by Shaanxi University of Technology, grant number SLGRC202416. Please remove any funding-related text from the manuscript and let us know how you would like to update your Funding Statement. Please include your amended statements within your cover letter; we will change the online submission form on your behalf.

**Response**: We have removed the funding-related text from the Acknowledgments section, and the funding statement: This research was funded by Shaanxi University of Technology, grant number SLGRC202416, is included in the cover letter.

5. We note that Figure(s) 1, 2, 3, 5, 6, 7, 8, 9, 10, 11 in your submission contain copyrighted images. All PLOS content is published under the Creative Commons Attribution License (CC BY 4.0), which means that the manuscript, images, and Supporting Information files will be freely available online, and any third party is permitted to access, download, copy, distribute, and use these materials in any way, even commercially, with proper attribution. For more information, see our copyright guidelines: http://journals.plos.org/plosone/s/licenses-and-copyright.

**Response**: We sincerely appreciate the reminder concerning the copyright and licensing issues of the figures in our manuscript.

We hereby confirm that all figures included in this manuscript are original works created by the authors and do not involve any third-party materials, copyrighted content, or previously published images, and none of the figures pose conflicts with the CC BY 4.0 licensing terms.

And we provide the original figures created with draw.io tool in the supplement materials.

6. If the reviewer comments include a recommendation to cite specific previously published works, please review and evaluate these publications to determine whether they are relevant and should be cited. There is no requirement to cite these works unless the editor has indicated otherwise.

**Response**: We thank the editor for the reminder concerning the citations suggested by the reviewers. We have carefully reviewed and evaluated all the recommended publications for their relevance to the present study. Those that are relevant and provide valuable support to our work have been appropriately cited in the revised manuscript. In accordance with the editorial guidance, we have not cited publications that are not directly relevant to the content of this paper.

**Reviewer #1:**

1. The abstract and conclusion need to be improved. The abstract must be a concise yet comprehensive reflection of what is in your paper. Please modify the abstract according to “motivation, description, results and conclusion” parts. I suggest extending the conclusions section to focus on the results you get, the method you propose, and their significance.

**Response**: We sincerely appreciate the valuable comments.

The abstract has been thoroughly revised to concisely and comprehensively reflect the manuscript, following the "motivation, description, results and conclusion" structure. The motivation clarifies SAM 2’s limitations in long-term VOS (error accumulation, poor occlusion robustness, inadequate adaptation to dynamic objects). The description details our proposed UAMP (a SAM 2 variant) and its core uncertainty-aware memory propagation framework with three key components. The results present key experimental findings, including UAMP’s superior performance across five VOS benchmarks, a maximum 5.6-point J&F improvement over SOTA methods, and stable performance in complex scenarios. The abstract’s conclusion summarizes UAMP’s effectiveness in enhancing SAM 2 for practical VOS.

The conclusion has been extended and optimized to focus on the proposed method, results, and their significance. We clarified UAMP’s core innovation and its targeted design to address SAM 2’s limitations. Key experimental results are elaborated, confirming UAMP’s effectiveness in mitigating error accumulation and adapting to dynamic objects, which provides a reusable module for VOS.

All revisions have been made in the revision.

2. The Section 1 and Section 2 is too short. I suggest the authors can merge section 1 and section 2.

**Response**: Thanks for your valuable suggestions. Section 1 (Introduction) focuses on the research background, SAM 2’s limitations in long-term VOS, the motivation of our UAMP method, and a brief overview of its core ideas, aiming to clarify the research gap and the necessity of our study. In contrast, Section 2 (Related Works) provides the related research, summarizing the development of VOS, and different paradigms, including the Memory-based VOS and Consistency Modeling, and highlighting the differences between these methods and our UAMP, helping readers grasp current research progress and our study’s innovation.

And further, according the following advices of 3 and 5, we further improve the introduction and related works section in the revision.

3. What is the motivation of the proposed method? The details of motivation and innovations are important for potential readers and journals. Please add this detailed description in the last paragraph in section I. Please modify the paragraph according to "For this paper, the main contributions are as follows: (1) ......" to Section I. Please give the details of motivations. In Section 1, I suggest the authors can amend your contributions of manuscript in the last of Section 1.

**Response**: Thanks for the suggestions. First, detailed descriptions of the motivation for the proposed UAMP method have been added to the last paragraph of Section I. The expanded motivation clarifies the research background (the wide application of SAM 2 as a foundational VOS model) and elaborates on the critical limitations of SAM 2 in long-term VOS tasks—specifically, error accumulation caused by its greedy-selection memory architecture, insufficient robustness to object occlusions and reappearances, and inadequate adaptation to dynamic appearance and motion variations of objects in complex scenarios.

Second, the contributions of the manuscript have been revised and placed at the end of Section I, following the format suggested by the reviewer: "For this paper, the main contributions are as follows: In summary, our main contributions are as follows: 1) We propose UAMP, a consistent video object segmentation model, which is specifically designed to address the inherent lim-itations of SAM 2 in long-term VOS tasks, with a core uncertainty-aware memory propagation framework, providing a reusable module for optimizing memory-based video segmentation models. 2) We introduce an uncertainty-aware memory propagation mechanism via re-gion-adaptive memory fusion by appearance and motion uncertainty estimation to refine the feature representation. At the meantime, a long-term memory updating mechanism and score-based short-term memory selection is designed to reduce the disturbance in crowded scenes by a mixture of motion and affinity scores. 3) Our UAMP achieves state-of-the-art performance on SA-V, MOSE, LVOSv1 and LVOSv2, and other VOT benchmarks, demonstrating effectiveness across diverse datasets".

4. The description of manuscript is very important for potential reader and other researchers. I encourage the authors to have their manuscript proof-edited by a native English speaker to enhance the level of paper presentation. There are some occasional grammatical problems within the text. It may need the attention of someone fluent in English language to enhance the readability.

**Response**: Thanks for the valuable suggestion regarding the manuscript’s presentation and English language readability. Specifically, we have invited a native English speaker with expertise in academic writing (focusing on computer vision and machine learning fields) to conduct a comprehensive proof-editing of the entire manuscript. And the proof-editing work has been completed in the revised manuscript.

5. The introduction section of the paper needs to revise according to the timeline of technology development. Please update references with recent paper in CVPR, ICCV, ECCV et al and Elsevier, Springer. In your section 1 and section 2, I suggest the authors amend several related literatures and corresponding references in recent years. For example: Dual Degradation Image Inpainting Method via Adaptive Feature Fusion and U-Net Network (Applied Soft Computing); CAAT: Image Super-resolution Algorithm via Channel Attention and Transformer (Array); MGNet: RGBT tracking via cross-modality cross-region mutual guidance (Neural Networks); Crack segmentation network via difference convolution-based encoder and hybrid CNN-Mamba multi-scale attention (Pattern Recognition)

**Response**: Thanks for the valuable advice. And the introduction section of the paper has been revised according to the timeline of technology development with the updated recent papers. Further, in section 1 and section 2, new related literatures have been amended in the revision.

Specifically, “SAM2Long [4] aimed to address the failure of SAM 2’s FIFO fixed-window memory mechanism in handling target reappearance by introducing a hierarchical memory tree structure for flexible memory retrieval and management, though it suffered from increased memory overhead, potential retrieval latency, and failed to resolve the confusion between simi-lar-looking instances during memory retrieval. SAMURAI [3] integrated Kalman filtering into SAM 2’s memory update process to model target motion trajectories and suppress error ac-cumulation through a prediction-update mechanism, yet its performance was heavily de-pendent on motion prediction accuracy in fast-changing scenarios, and it could not effectively handle severe target deformation that led to large prediction errors. DAM4SAM [5], proposes a novel distractor-aware memory (DAM) module for the SAM 2 model, which is composed of recent appearance memory (RAM) and designed to address the interference of distractor objects (irrelevant objects with similar appearance to the target) in VOS tasks. However, DAM4SAM over-relies on the recent appearance features stored in RAM, making it less adaptable to scenarios where the target undergoes significant appearance changes over time. DC-SAM [45] leverages multi-source features and generates positive and negative prompts by ensuring prompt consistency, integrating with SAM/SAM2 to achieve in-context segmentation for both images and videos. and improves the model's adaptability to complex scenes. But it focuses on prompt-tuning adaption, and lacks effective long-term temporal correlation modeling capabilities, making it difficult to maintain consistent segmentation performance when the target undergoes continuous appearance changes (such as scale variation, deformation) in long video sequences”.

6. Please give the details of proposed method for proposed model. I suggest the authors amend the calculation of your size of proposed method and the details is important for proposed method.

**Response**: We sincerely appreciate your valuable comment. Following your suggestion, we have supplemented the detailed implementation and dimensional calculation of each core module of the proposed model, and revised the relevant content in the section 3 and section 4.12 of model details.

Specifically, “For image encoder, the input image is first scaled to the resolution of 1024, and the image embeddings are obtained by fusing the stride 16 and 32 features from Stages 3 and 4 of the Hiera [25] image encoder with the dimension 448×64×64 and 896×32×32 respectively. These multi-scale features are then fused via a pyramid network following [1], and model dimension of Hiera is 256. The positional embeddings are calculated with the windowed absolute positional embeddings, and the positional information spanning across windows is obtained by interpolating the positional embeddings. For memory attention, the input feature map is the 256×64×64 embeddings generated by the memory encoder, the 2d spatial Rotary Positional Embedding (RoPE) [30] with the dimension of 256 is utilized in both self-attention and cross-attention layers, and this module composes 4 layers. For mask decoder, the stride 4 and 8 features from Stages 1 and 2 are added to the up-sampling layers to get high-resolution features. Following [6], the appearance uncertainty prediction module comprises one 1×1 convolution and two 3×3 convolutions. It takes the input of a concatenation of current frame feature, last frame feature, and last mask prediction, and outputs a one-channel change probability mask of the query token. For memory bank, the memory features 256×64×64 are projected to the dimension of 2048 for 8 memory banks via a linear layer, and the short-term memory selection is designed follows DAM [5] as discussed in Section 3.3. The long-term memory updating composes 4 layers of the transformer-like structure, in which the self-attention layer is replaced with the linear-attention layer. And the matrix state is projected to the same dimension as short-term memory with a linear projection layer.”

7. The content of experiments needs to amend related experiments to compare related SOTA in recent three years. I recommend the authors amend related experimental results of proposed method of SOTA according to the published paper in IEEE, Springer and Elsevier.

**Response**: We sincerely appreciate your comment. We have amended related experimental results with the state-of-the-art (SOTA) methods in the field published in the past three years in the Table 1 and Table 2 of section 4.2.

Specifically, we compare with the Cutie-bas [20] method presented at CVPR 2024, LiVOS [46] at CVPR 2025, SAM2.1[1] at ICLR 2025, and SAM2.1Long [4] at ICCV 2025. All the selected approaches provide complete open-source implementations or detailed experimental protocols in their original papers, which ensures the fairness of our comparative evaluation.

8. However, the manuscript, in its present form, contains several weaknesses. Adequate revisions to the following points should be undertaken in order to justify recommendation for publication.

**Response**: We sincerely appreciate the reviewer for the constructive comments and valuable suggestions. We fully agree that the manuscript needs further improvement to enhance its quality. We have carefully revised the manuscript according to the comments, and the detailed revisions are addressed point by point in the revision.

9. In the conclusion section, the limitations of this study and suggested improvements of this work should be highlighted.

**Response**: We sincerely appreciate the reviewer’s constructive suggestion. Following this comment, we have supplemented and highlighted the limitations of our study and potential future improvements in the conclusion section.

Specifically, our method still fails to resolve precise boundary information in some challenging scenarios. To address this issue, we suggest two promising directions for future work: First, integrating more detailed feature representations, e.g., adopting high-quality feature extraction paradigms like HQ-SAM. Second, fine-tuning the proposed UAMP on matting datasets to further refine edge details.

10. Provide a critical review of the previous "journal" (not conference) papers in the area and explain the inadequacies of previous approaches.

**Response**: We sincerely appreciate the valuable comment. We have revised the related work section to provide a critical review of previous journal papers in this research area.

In the revised manuscript, we summarize and analyze the representative journal works in Section 1 and Section 2, and clearly point out their limitations and inadequacies, such as “SAM2Long [4] aimed to address the failure of SAM 2’s FIFO fixed-window memory mechanism in handling target reappearance by introducing a hierarchical memory tree structure for flexible memory retrieval and management, though it suffered from increased memory overhead, potential retrieval latency, and failed to resolve the confusion between simi-lar-looking instances during memory retrieval. SAMURAI [3] integrated Kalman filtering into SAM 2’s memory update process to model target motion trajectories and suppress error ac-cumulation through a prediction-update mechanism, yet its performance was heavily de-pendent on motion prediction accuracy in fast-changing scenarios, and it could not effectively handle severe target deformation that led to large prediction errors ….”. By comparing with these typical approaches, we further emphasize the motivation and advantages of our proposed method.

11. I suggest the authors revise Section 1 and Section 2. Please revise the content according to the development of timeline.

**Response**: We sincerely appreciate the valuable suggestion. In the revised manuscript, we thoroughly adjust the content of Section 1 and Section 2 to follow the chronological order of the field’s development. Specifically, in Section 1, we will reorganize the introduction of the research background, research problems, and the evolution of related technologies, clearly presenting how the research topic has developed over time and highlighting the necessity of our work in the context of this timeline. In Section 2, we sort out the representative studies in the field in chronological sequence, from the early foundational works to the latest research progress, and systematically analyze the improvement, and innovation among different studies.

12. Please check all parameters in the manuscript and amend some related description of primary parameters. In section 3, please write the proposed algorithm in a proper algorithm/pseudocode format with section 3. Otherwise, it is very hard to follow. Some examples here: https://tex.stackexchange.com/questions/204592/how-to-format-a-pseudocode-algorithm

**Response**: We sincerely appreciate constructive comment. We have comprehensively reviewed all parameters in the manuscript, and amended primary parameters in Section 4 to ensure clarity and reproducibility; meanwhile, we have rewritten the proposed UAMP algorithm in a standard academic pseudocode format embedded in Section 3, which includes clear input/output, standardized variable symbols consistent with the manuscript, step-by-step logic, core sub-module definitions and explicit references to relevant mathematical formulas in Section 3.

Algorithm 1 Proposed UAMP Method.

Input: video frames $\{I_{1},I_{2},\cdots,I_{N}\}$, initial prompt $P_{0}$

Output: masks $\{M_{1},M_{2},\cdots,M_{N}\}$

Initialize memory bank $f_{mem}^{i}, i\in\{1,2\cdots,7\}$ and long-term memory $S_{mem}^{i}$

Initialize object token $P_{token}$

for t = 1 to N do

$f_{t},P_{token},s_{mask}^{t},s_{obj}^{t},M_{t}\leftarrow SAM2(I_{t})$ $⊳$ get image feature, prompt embedding, mask score, object score and masks.

if t > 1 then

Query current features $f_{t}^{m}$ from memory bank $f_{mem}^{i}$ and $S_{mem}^{i}$ using Equation (5)

Update image feature $f_{t}$ using Equation (4)	$⊳$ appearance uncertainty prediction

Get $s_{kf}^{t}, M_{t}$ using Equation (6)	$⊳$ motion uncertainty estimation

Get updated features $F_{t}'$ using Equation (7)

Update long-term memory $S_{t}$ using Equation (11) $⊳$ long-term memory updating

Update short-term memory $f_{mem}^{i}$ using Equation (12) $⊳$ short-term memory selection

else

put $f_{t}$ to memory bank $f_{mem}^{i}$

end if

end for

**Reviewer #2:**

1. UAMP uses long-term and short-term memory. How is the balance between these two memories determined and is it adaptable for different videos?

**Response**: Thanks for your valuable advice. The balance between long-term and short-term memory in UAMP is dynamically regulated via learnable gating and attention mechanisms as shown in Section 3.3.3. Specifically, the short-term memory maintains recent frame features, governed by the selection mechanism that replace the irrelevant local information. Long-term memory stores global semantic context and historical representations, updated by a sustained memory cell with slow refresh.

Their fusion weight is learned end-to-end during training with memory encoder and memory attention module, automatically adjusting based on temporal correlation, scene complexity, and task objectives. For videos with fast motion or frequent scene changes, the model relies more on short-term memory to capture local dynamics. or videos with slow changes, long narratives, or consistent semantics, long-term memory is weighted more strongly to preserve global consistency.

2. J&F increase of up to 5.6 has been reported. Is this improvement the same for all object classes or only for specific motion patterns?

**Response**: We appreciate the insightful question. The reported J&F improvement of up to 5.6 is computed as the average over all object classes and test sequences, where the J&F metric is defined as the mean of the Jaccard index and F-measure for overall segmentation quality, to keep coincident with other papers for comparison. This average gain is not identical across all categories but is more pronounced for object classes with complex motion patterns, non-rigid deformation, and fast temporal variations, as our method better maintains temporal coherence and segmentation accuracy in these challenging scenarios.

3. The goal of the UAMP paper is clear, but how does this model ensure that the combination of uncertainty and dual memory in long videos and crowded scenes actually provides stable performance?

**Response**: Thanks for this valuable comment. The model ensures stable performance when combining uncertainty estimation and dual memory in long videos and crowded scenes via several carefully designed mechanisms. First, the uncertainty module dynamically weights unreliable features caused by occlusion, motion blur, and clutter, suppressing noisy or ambiguous representations before they enter the memory system. Second, the dual long‑short term memory structure maintains both global temporal context and local frame‑to‑frame consistency, preventing error accumulation and drift in long sequences. Third, the uncertainty‑aware short-term memory selection mechanism and gated long-term memory updating mechanism adjusts the balance between historical memory and current observations, prioritizing reliable cues while attenuating unstable ones in crowded or complex regions. Together, these components prevent the model from being distracted by noisy or uncertain regions in crowded scenes and avoid degradation over extended video durations, leading to robust and stable segmentation performance across challenging scenarios.

We make this clearer in Section 3 in the revision.

4. Most studies focus on short-term or long-term memory, but how can one strike an optimal balance between the two for long videos and crowded scenes?

**Response**: Thanks for this insightful question. Built upon the memory attention mechanism of SAM2, our method achieves an adaptive balance between short-term and long-term memory for long videos and crowded scenes via structurally distinct yet mutually complementary designs. Specifically, we adopt a gating mechanism of long-term memory to retain stable global semantic context and persistent object representations, effectively mitigating temporal drift and error accumulation in extended sequences. For short-term memory, we introduce a selection mechanism to extract reliable local temporal dynamics and fine-grained appearance cues from recent frames, suppressing noisy or ambiguous information caused by occlusion, motion blur, and clutter. Under the unified framework of SAM2’s memory attention, these two components are dynamically integrated: the gating of long-term memory ensures robust context propagation, while the selection of short-term memory enhances precise temporal alignment. This structure strikes an optimal balance, automatically prioritizing stable long-term context in crowded or uncertain regions and switching to fine-grained short-term cues in clear regions, thus delivering consistent and stable performance for long videos and complex crowded scenes as shown in the experiment results of Section 4.

5. Motion- or appearance-based models each have their limitations, but is there an approach that simultaneously exploits uncertainty in motion and appearance to improve coherence?

**Response**: We thank the reviewer for this insightful question. Indeed, several prior works have explored joint modeling of uncertainty in both motion and appearance, which we briefly summarize and contrast with our approach.

First, nonparametric joint entropy modeling (Boltz et al., 2008) defines a unified energy function that simultaneously penalizes motion inconsistency and appearance ambiguity, avoiding parametric assumptions and enabling robust joint uncertainty estimation. Second, probabilistic recursive fusion methods (Mengers et al., 2016) use interconnected recursive estimators to jointly infer motion and appearance uncertainties, where each cue’s uncertainty dynamically constrains the other during inference, ensuring consistent probabilistic segmentation. Third, co-attention mechanisms (Yang et al., 2021) integrate motion and appearance uncertainties directly into attention weights, adaptively suppressing unreliable cues in a unified feature.

While existing methods that jointly model uncertainty in motion and appearance typically suffer from several critical limitations in long videos and crowded scenes. First, joint probabilistic inference often requires intricate probabilistic modeling, making it difficult to integrate into modern foundation models like SAM2 that rely on efficient memory attention mechanisms. Second, unified motion-appearance uncertainty learning tends to lose modality-specific characteristics—motion ambiguities caused by occlusion or fast deformation and appearance ambiguities caused by illumination or texture changes follow distinct distributions, and forcing them into a single joint representation may suppress detailed cues that are critical for stable segmentation. Third, most joint estimation strategies (eg. co-attention mechanisms (Yang et al., 2021)) lack explicit temporal memory mechanisms, so they cannot effectively propagate reliable information across long sequences or suppress error accumulation in crowded scenarios. In contrast, our approach, built on SAM2’s memory attention with a gating mechanism for long-term memory and a selection mechanism for short-term memory, maintains the complementary advantages of motion and appearance while adaptively balancing their uncertainties within a robust temporal memory framework, avoiding the above limitations and achieving more stable and coherent performance.

6. Previous methods such as XMem and SAM2Long have complex and expensive memory, but how can high performance be maintained at a reasonable computational cost?

**Response**: Thanks for the valuable comment. Previous state-of-the-art methods like XMem and SAM2Long often adopt complex memory architecture, such as dense historical feature storage, multi-scale memory banks, or iterative cross-memory fusion to pursue high performance, which inevitably leads to high computational and memory costs, making them less feasible for long videos and real-world applications. To address this trade-off between performance and computational cost, our UAMP framework, built on SAM2’s memory attention mechanism, achieves efficient yet robust performance through the key designs. First, instead of dense memory storage, we adopt an adaptive memory update strategy: long-term memory (governed by a gate mechanism) only retains stable global semantic context and discards redundant or noisy historical features, while short-term memory (via a selection mechanism) only extracts reliable local cues from recent frames, avoiding unnecessary feature storage and computation. Second, we leverage the inherent efficiency of SAM2’s memory attention and utilize uncertainty-aware selection to dynamically integrate motion and appearance cues in a single pass, rather than iterative fusion, to reduce the computational overhead while ensuring the memory system focuses on valuable information.

7. Most studies have focused on single or simple objects, but how can one solve VOS in a stable manner in crowded scenes with similar and hidden objects?

**Response**: We thank the reviewer for this insightful question. Most prior works focus on single or simple objects, where inter-object interference is minimal, but they struggle to maintain stability in crowded scenes with similar and hidden objects, mainly due to ambiguous feature discrimination, frequent occlusion-induced drift, and confusion between adjacent similar targets. To tackle this problem, our UAMP framework, built on SAM2’s memory attention mechanism, achieves stable VOS in such challenging scenarios through three targeted designs integrated with our uncertainty-aware dual memory system. First, for similar objects in crowded scenes, we jointly model motion and appearance uncertainties to enhance feature discriminability: by assigning higher weights to modality-specific reliable cues (e.g., subtle motion differences or unique appearance details) and suppressing ambiguous shared features, our model can effectively distinguish between visually similar objects that are closely clustered. Second, to handle hidden objects (occlusion), our long-term memory (governed by a gate mechanism) retains stable global semantic and structural context of all objects in the scene, even when they are partially or fully occluded; this prevents the model from losing track of hidden objects and avoids drift when objects re-appear. Third, our short-term memory (via a selection mechanism) dynamically extracts local temporal cues from recent frames, focusing on the visible parts of occluded objects and the fine-grained differences between adjacent similar targets, further reinforcing segmentation stability. Unlike previous methods that rely solely on single-cue memory or lack targeted handling of crowded scenarios, our uncertainty-guided dual memory design (gate for long-term context preservation, selection for short-term detail extraction) balances global context retention and local discriminability, enabling stable, accurate VOS even in crowded scenes with similar and hidden objects, which is verified by our experiments on complex crowded datasets in Fig 9 and Fig 10.

8. How does Figure 2 relate to Relationship 1?

**Response**: Thanks for this valuable comment. We believe the reviewer is referring to the relationship between Figure 1, Figure 2 and Figure 3. Figure 1 presents the whole SAM 2 architecture; Figure 2 further visualizes the existing methods based on SAM2 (introduced in Figure 1) for video object segmentation illustrates the detailed mechanism. Figure 3 gives the proposed method based on existing methods shown in Figure 2.

Together, Figure 1, Figure 2 and Figure 3 work further progressively: Figure 1 provides the base model compo, Figure 2 shows the existing methods based on Figure 1, while Figure 3 offers detailed support and explanation for the key idea.

9. In long-term memory design, how is it determined how much of the old memory should be forgotten to both maintain performance and reduce computational cost?

**Response**: We thank the reviewer for this critical question. In our long-term memory design, the amount of old memory to be forgotten is dynamically determined by a linear attention gating mechanism that relies entirely on input data to automatically compute gating weights, achieving a balance between performance preservation and computational cost reduction. Specifically, built on SAM2’s memory attention framework, the linear attention gate adaptively computes continuous gating weights, for noisy features from occlusion or motion blur or weak temporal correlation with the current scene, the gate assigns lower weights to discard redundant, unstable information and reduce unnecessary memory storage/computation. For old memory that contains stable global semantic context, persistent object structures, or strong temporal consistency with the current frame, the gate assigns higher weights to retain valuable information that is critical for maintaining segmentation stability in long videos and crowded scenes. Furthermore, our linear attention gate operates efficiently with O(N) complexity, leveraging data-driven automatic weight calculation to precisely control the forgetting ratio: this not only avoids the computational overhead of retaining redundant old memory but also ensures that essential context is preserved to maintain high performance, perfectly addressing the trade-off between memory efficiency and segmentation stability in long-term memory design.

10. Given the higher volatility of the motion score than the object score, how do the authors ensure that the weight chosen is optimal for combining motion and appearance uncertainty and is stable under different conditions (occlusion and reappearance of objects)?

**Response**: Thanks for the valuable comment. Given the higher volatility of motion scores compared to appearance scores, we fuse motion and appearance uncertainties via an uncertainty mask to achieve stable and optimal weighting as illustrated in Section 3. The uncertainty mask is directly derived from motion and appearance reliability, automatically down-weighting noisy or volatile motion signals during occlusion, and emphasizing stable appearance cues. This data-driven, mask-based fusion ensures adaptive, robust combination under varying conditions, maintaining stable and consistent performance. To address the higher volatility of motion scores relative to appearance scores, the fusion features are automatically learned via data-driven linear attention gating, where the gate dynamically adjusts contributions from motion and appearance uncertainty: under occlusion, motion signals become unreliable, so the gate suppresses volatile motion features and strengthens stable appearance-based memory; when objects reappear, it quickly restores motion cues to recover temporal alignment. This adaptive mechanism ensures the combined score remains optimal and stable across severe occlusion, rapid deformation, and object reappearance.
