# Supplementary material for: UAMP: Consistent video object segmentation with uncertainty-aware memory propagation
Source: PLoS One. 2026 Jul 7;21(7):e0353156. doi: 10.1371/journal.pone.0353156 (PMC13718450; doi:10.1371/journal.pone.0353156)
Supplement: Supplementary file 2 [file pone.0353156.s003.docx]

**For Editor:**

Required revisions:

*1) Clarification and formal definition of uncertainty modules*

*The manuscript should clearly define the appearance and motion uncertainty components and describe how these quantities are supervised or learned during training. The current description remains partly conceptual and does not fully specify the mathematical formulation.*

**Response**: Thank you for your careful and constructive comment. We have further strengthened the mathematical definition and learning mechanism of appearance uncertainty and motion uncertainty to avoid conceptual ambiguity.

Specifically, we have clearly defined the formulation of appearance uncertainty in Eq (5) and its corresponding supervised loss in Eq (6), which explicitly guides the learning of appearance reliability during training. For motion uncertainty, we have provided a precise mathematical formulation in Eq (8-9). In the revised manuscript, we have also added detailed explanations for each component, clarifying how these uncertainty values are modeled, optimized, and integrated into the overall training pipeline. All components are now fully specified with explicit mathematical expressions, ensuring the description is concrete and reproducible.

*2) Reproducibility of the proposed framework*

*Several key elements of the method require clearer specification to ensure reproducibility, including:*

*a) the gating mechanism used for balancing long-term and short-term memory*

*b) the exact formulation of the uncertainty estimation modules*

*c) the loss functions and weighting schemes used during training*

*d) the full specification of the Kalman filter used for motion scoring*

**Response**: Thank you for your valuable comment on ensuring the reproducibility of our method. We have carefully addressed each of the key elements highlighted, with detailed specifications and explicit formulations supplemented throughout the revised manuscript:

For a) We have mathematically formalized this mechanism in Eq (14), Eq (15), and Eq. (16). Specifically, Eq. (14) clearly defines the specific normalization strategies for the query and key components, and we have provided thorough, step-by-step explanations in the accompanying paragraphs to clarify how the gating weights in Eq (16) are computed.

For b) The formulations for appearance uncertainty and motion uncertainty estimation are explicitly presented in Eq (5) and Eq (9), respectively. We have added detailed definitions of all variables and symbols in these equations, ensuring the mathematical formulation of each uncertainty module is fully specified—no conceptual ambiguity remains, and the implementation logic is clearly elaborated.

For c) The training loss functions corresponding to the uncertainty estimation modules are detailed in Eq (6) and Eq (18). Additionally, the weighting schemes that integrate these uncertainty values into the overall framework are explicitly defined in Eq (4) and Eq (10), with clear descriptions of how these weights are applied during training to optimize the model.

For d) We have supplemented comprehensive Kalman filter formulations for motion uncertainty estimation in Eq (8) and Eq (9), along with clear explanations of all symbols and variables in Section 3.3.2. Furthermore, we have provided the exact definitions of the state transition matrix, measurement matrix, process noise covariance, observation noise covariance, and initial error covariance matrix, along with their specific experimental values to fully support the reproducibility of the motion uncertainty estimation module.

In addition, the pseudocode in Section 3 has been updated accordingly to incorporate all the above specifications, ensuring every key step of the proposed method is fully detailed and can be implemented.

*3) Algorithmic clarity and symbol definitions*

*All variables and symbols appearing in the equations should be explicitly defined. The pseudocode and equations should be sufficiently detailed to allow independent implementation.*

**Response**: Thank you for your valuable comment. We have revised the entire manuscript to explicitly define every variable, symbol, and subscript in all equations, with clear descriptions immediately following each formula in Section 3.

In addition, we have further expanded the pseudocode and mathematical formulations with step-by-step implementation details, including parameter settings, matrix dimensions, iterative procedures, and computation flows in Algorithm 1 and Section 4 model and train details. All critical components are described with sufficient specificity to enable independent re-implementation of the proposed method in the manuscript.

*4) Experimental clarity and evaluation reporting*

*The evaluation section would benefit from clearer reporting of experimental settings, including hyperparameters, memory bank configuration, and training schedules. Where possible, provide additional breakdown of performance (e.g., per-category or scenario-level results) to support the claims regarding improved robustness in challenging scenarios such as occlusion or crowded scenes.*

**Response**: Thank you for your valuable comment. We have detailed a dedicated table that explicitly summarizes all key hyperparameters, including the number of memory banks, learning rate schedule, batch size, optimizer settings, and other related configurations in **Table 1** and explained the train details part in Section 4.1.2.

About providing the additional performance, we sincerely appreciate the constructive suggestion on providing more detailed breakdowns to further verify the advantages of UAMP in crowded and occluded scenarios. However, we would like to clarify that the semi-supervised VOS is inherently a class-agnostic task, where given an initial mask, bounding box, or interactive clicks in the first frame, the model only needs to separate the specified target from the background, without relying on semantic category labels. Secondly, none of the compared SOTA methods (Cutie-bas, LiVOS, SAM2.1, SAM2.1Long) reports per-class or per-category J&F metrics in their original papers or official implementations. The official evaluation protocols of the benchmark datasets (e.g., LVOS) also do not provide pre-defined class/category splits for computing such fine-grained per-class statistics. Manually defining arbitrary categories and computing breakdowns alone would lead to incomparable and non-reproducible results against existing SOTAs.

To substantiate our claim that UAMP performs favorably in crowded and occluded scenarios, we evaluated the proposed method on six different datasets, including MOSE, SA-V, LVOSv1, LVOSv2, DAVIS and YouTube-VOS, in which the LVOS is designed for long-term video object segmentation, MOSE, the complex real-life dataset, contains the crowded and occluded scenarios, and SA-V has small, occluded, and reappearing objects in the video sequences. Following the standard evaluation protocol widely accepted in the VOS field, we report overall J&F scores in line with representative recent works. To better substantiate our claim that UAMP performs favorably in crowded and occluded scenarios, we have done the qualitative and quantitative analysis on challenging sequences, highlighting the advantages of our method under complex conditions in Section 4.2.

*5) Data availability statement*

*One reviewer indicated concerns regarding the availability of underlying data. Please ensure that all data required to reproduce the reported findings are publicly available or clearly described in accordance with PLOS ONE’s data availability policy.*

**Response**: Thank you for your reminder regarding the data availability policy. We fully comply with the requirements of PLOS ONE and confirm that all datasets used in this study are publicly accessible standard benchmarks, including MOSE, SA-V, LVOS, DAVIS, and YouTube-VOS, which are widely adopted in the video object segmentation community and freely available to the research community, and download sources are provided in the data availability statement.

We mixed 1% SA-V dataset, 2% MOSE dataset and 1% LVOS dataset for the training dataset, specifically:

1) The SA-V dataset is composed of the videos from sav_000001 to sav_000099 of the sav_000.tar,

2）The MOSE dataset is composed of the videos 10552818 to

Recommended improvements:

*1) Improve the structure and clarity of section introductions so that each section and subsection begins with a brief explanation of its purpose.*

**Response**: Thanks for this valuable comment. In the revised version, we have added concise introductory paragraphs at the beginning of the main section to briefly outline the purpose, content, or the organization of the following subsections. These short introductions help guide readers and improve the logical flow of the manuscript in Section 3. We have applied this revision across all relevant sections and subsections to ensure a formal and professional writing style.

2) Ensure consistent terminology throughout the manuscript (e.g., “uncertainty”, “change probability”, “memory bank”, etc.).

**Response**: Thanks for this valuable comment. We have standardized the terminology throughout the manuscript to maintain consistency. We have unified the use of “uncertainty” instead of inconsistent mixing with “change probability” and we have maintained a clear and consistent distinction between memory bank (referring to the entire memory module and storage structure) and memory (referring to the stored features and information within the bank) to remain consistent with SAM2.

3) Provide a brief discussion of computational overhead and inference latency compared with the baseline SAM2 framework.

**Response**: Thanks for this valuable comment. We quantitatively compare the proposed UAMP framework with the baseline SAM2 model in terms of memory footprint and inference latency, and analyze the computational complexity and scalability of our method. The corresponding runtime analysis have been added in Section 4.2.2 to clearly demonstrate the efficiency and practicality of UAMP while maintaining performance improvement, which is detailed in Table 9.

4) Review the suggested references provided by the reviewers and cite them where appropriate.

**Response**: We appreciate the suggested references for potential citation. We have carefully reviewed the research focus of each recommended work:

Paper a) centers on multi-modal feature fusion within a Transformer architecture for integrating visual and audio information to advance dense video captioning. Paper b) explores the design and technological roadmap for developing zero‑emission electric vessels. Paper c) aims at investigating the effect of cigarette smoke exposure on the systemic circulation and local airway 5-HT levels as well as MAO-mediated oxidative pathway using a cigarette smoke-exposed rat model. Paper d) presents device-level simulation and analysis of In₀.₅₃Ga₀.₄₇As implant‑free quantum‑well structures in microelectronics.

Our work targets video object segmentation (VOS), a core task in video understanding that aims to achieve pixel‑level foreground object separation and consistent tracking across consecutive video frames. Given that the above references belong to different research domains, including video captioning, marine engineering, biomedicine, and microelectronic device design, they are not thematically or methodologically aligned with our focus on video object segmentation. Accordingly, we respectfully consider these references less relevant to the present study. We will continue to enrich our literature review by incorporating more state‑of‑the‑art and representative studies dedicated to video object segmentation to better contextualize our contributions for future work.

**Reviewer #4**

*1). The pseudocode in Section 3 is a good addition, but some steps remain underspecified. For example:*

*a. The gating mechanism for balancing long-term and short-term memory is described qualitatively, but the exact mathematical formulation (activation function, normalization strategy) is missing.*

*b. The uncertainty estimation modules (appearance vs. motion) are introduced, but the training loss functions and weighting schemes are not fully detailed.*

**Response**: Thanks for your constructive and valuable comments. We have further refined the relevant descriptions and mathematical formulations to clarify the underspecified details in the pseudocode.

Specifically, we have updated the uncertainty estimation modules in Sections 3.3.1 and 3.3.2. The formulations for appearance uncertainty and motion uncertainty estimation are clearly presented in Eq (5) and Eq (9), the corresponding training loss functions are detailed in Eq (6) and Eq (18), where motion uncertainty is modeled using a Kalman filter, which has an analytical solution, and the weighting schemes are defined in Eq (4) and Eq (10). In addition, the gating mechanism used to balance long‑term and short‑term memory has been mathematically formalized in Eq (14), Eq (15) and Eq (16), including the specific activation functions and normalization strategies about query and key, with thorough explanations provided in the paragraphs. The pseudocode in Section 3 has also been updated accordingly to ensure all key steps are fully specified and reproducible.

*2).* *The author(s) introduces additional modules (uncertainty prediction, dual memory mechanisms), but there is no discussion of computational overhead. How does UAMP scale in terms of memory footprint and inference latency compared to SAM2?*

**Response**: We appreciate the reviewer for this comment. In the revised manuscript, we have supplemented a detailed discussion on the computational overhead introduced by the proposed uncertainty prediction and memory management modules. We quantitatively compare the proposed UAMP framework with the baseline SAM2 model in terms of memory footprint and inference latency, and analyze the computational complexity and scalability of our method. The corresponding runtime analysis have been added in Section 4.2.2 to clearly demonstrate the efficiency and practicality of UAMP while maintaining performance improvement, which is detailed in Table 9.

*3). Although recent SOTA methods (Cutie-bas, LiVOS, SAM2.1, SAM2.1Long) are included, the evaluation could be expanded: Report per-class or per-category breakdowns of J&F improvements to substantiate claims that UAMP excels in crowded or occluded scenarios.*

**Response**: We sincerely appreciate the constructive suggestion on providing more detailed breakdowns to further verify the advantages of UAMP in crowded and occluded scenarios.

However, we would like to clarify that the semi-supervised VOS is inherently a class-agnostic task, where given an initial mask, bounding box, or interactive clicks in the first frame, the model only needs to separate the specified target from the background, without relying on semantic category labels. Secondly, none of the compared SOTA methods (Cutie-bas, LiVOS, SAM2.1, SAM2.1Long) reports per-class or per-category J&F metrics in their original papers or official implementations. The official evaluation protocols of the benchmark datasets (e.g., LVOS) also do not provide pre-defined class/category splits for computing such fine-grained per-class statistics. Manually defining arbitrary categories and computing breakdowns alone would lead to incomparable and non-reproducible results against existing SOTAs.

To substantiate our claim that UAMP performs favorably in crowded and occluded scenarios, we evaluated the proposed method on six different datasets, including MOSE, SA-V, LVOSv1, LVOSv2, DAVIS and YouTube-VOS, in which the LVOS is designed for long-term video object segmentation, MOSE, the complex real-life dataset, contains the crowded and occluded scenarios, and SA-V has small, occluded, and reappearing objects in the video sequences. Following the standard evaluation protocol widely accepted in the VOS field, we report overall J&F scores in line with representative recent works. To better substantiate our claim that UAMP performs favorably in crowded and occluded scenarios, we have done the qualitative and quantitative analysis on challenging sequences, highlighting the advantages of our method under complex conditions in Section 4.2.

*4). The manuscript alternates between “uncertainty-aware memory propagation” and “region-adaptive fusion.” It would help to standardize terminology to avoid confusion.*

**Response**: Thank you for this constructive comment. We apologize for any confusion caused by inconsistent terminology usage in the manuscript. We have carefully revised the entire text to standardize and unify the naming conventions for all key components with “uncertainty-aware memory propagation”.

*5). Section 4 mentions dimensional calculations, but hyperparameter choices (e.g., number of memory banks, learning rate schedules) should be explicitly tabulated for clarity.*

**Response**: Thank you for this valuable and constructive suggestion. We agree that clearly presenting hyperparameter settings is essential for reproducibility and readability.

In the revised manuscript, we have added a dedicated table that explicitly summarizes all key hyperparameters, including the number of memory banks, learning rate schedule, batch size, optimizer settings, and other related configurations in **Table 1**. Meanwhile, we have standardized the description of dimensional calculations in Section 4 to ensure full transparency of the implementation details. All these revisions enhance the reproducibility of our work.

*6). Some related works are recommended for citation:*

*a. https://doi.org/10.3390/s23125565*

*b. https://doi.org/10.1109/PESA.2015.7398965*

*c. https://doi.org/10.3109/10715762.2012.721928*

*d. https://doi.org/10.1016/j.mee.2010.11.019*

**Response**: We appreciate the suggested references for potential citation. We have carefully reviewed the research focus and contributions of each recommended work:

Paper a) centers on multi-modal feature fusion within a Transformer architecture for integrating visual and audio information to advance dense video captioning. Paper b) explores the design and technological roadmap for developing zero‑emission electric vessels. Paper c) aims at investigating the effect of cigarette smoke exposure on the systemic circulation and local airway 5-HT levels as well as MAO-mediated oxidative pathway using a cigarette smoke-exposed rat model. Paper d) presents device-level simulation and analysis of In₀.₅₃Ga₀.₄₇As implant‑free quantum‑well structures in microelectronics.

Our work targets video object segmentation (VOS), a core task in video understanding that aims to achieve pixel‑level foreground object separation and consistent tracking across consecutive video frames. Given that the above references belong to different research domains, including video captioning, marine engineering, biomedicine, and microelectronic device design, they are not thematically or methodologically aligned with our focus on video object segmentation. Accordingly, we respectfully consider these references less relevant to the present study. We will continue to enrich our literature review by incorporating more state‑of‑the‑art and representative studies dedicated to video object segmentation to better contextualize our contributions for future work.

**Reviewer #5:**

*All the comments form the reviewers are addressed. However I noticed that subsections are directly started without any information about that section example section 3, 3.1 is directly written there is no information or description. Same thing is repited in next section and its subsection. This is not a professional way of technical writing.*

**Response**: We thank the reviewer for this valuable comment. In the revised version, we have added concise introductory paragraphs at the beginning of the main section to briefly outline the purpose, content, or the organization of the following subsections. These short introductions help guide readers and improve the logical flow of the manuscript in Section 3. We have applied this revision across all relevant sections and subsections to ensure a formal and professional writing style.

**Reviewer #6:**

*1) Define “uncertainty” and its supervsion precisely (appearance and motion)*

**Response**: Thanks for your valuable comments. We have precisely defined appearance uncertainty and its corresponding supervision in Eq (5) and Eq (6), respectively. For motion uncertainty, we have provided a clearer and more rigorous formulation in Eq (8) and Eq (9). Specifically, motion uncertainty is modeled using a Kalman filter, which allows for an analytical solution, and we have supplemented detailed explanations to ensure full clarity of both uncertainty definitions and their supervisory objectives in Section 3.3.1 and 3.3.2.

*2) Assure all symbols in the eqs are defined and self-consistent*

**Response**: Thanks for your valuable comments. We have carefully checked all equations throughout the manuscript and ensured the symbol and notation appearing in the equations is clearly defined when first mentioned. In addition, we have verified that all symbols are consistent with uniform definitions across the entire text to avoid ambiguity and maintain consistency. The corresponding revisions have been made in the revised manuscript.

*3) Provide full Kalman filter details (reproducibility)*

**Response**: Thank you for your valuable comment. We have supplemented detailed Kalman filter formulations for motion uncertainty estimation, including full derivations in Eq (8) and Eq (9), along with clear explanations of all symbols and variables in Section 3.3.2.

In addition, we have provided the exact definitions of the state transition matrix, measurement matrix, process noise covariance, observation noise covariance, and initial error covariance matrix with their specific experimental values to support the reproducibility of the motion uncertainty estimation module.

*4) Short-term memory selection rule seems underspecified (Eq. 12)*

**Response**: Thanks for your valuable comment. We have supplemented a more detailed explanation for the short-term memory selection rule corresponding to Eq (11), including the design logic, selection criteria, and implementation details. The formulation and related descriptions have been refined to clearly specify how informative and reliable memory entries are selected, ensuring the mechanism is fully specified and reproducible.

*5) Long-term memory updating needs clearer integration with SAM 2 memory bank*

**Response**: Thanks for your valuable comment. To better illustrate how the long-term memory updating mechanism is integrated with the SAM 2 memory bank, we have provided the overall architecture diagram in Fig.3 that visualizes the entire pipeline. Furthermore, we have added Fig.4 with detailed structural designs to explicitly highlight the differences and connections between the original SAM2 memory bank and the improved UAMP memory bank. These supplementary illustrations and detailed descriptions clarify the working logic of long-term memory updating and its integration with SAM2’s memory mechanism, making the framework more intuitive and understandable.

*6) The Discussion section currently starts abruptly; it needs improvement to following the scientific writing standards*

**Response**: Thank you for your constructive suggestion. In the revised manuscript, we have rephrased the beginning of the Discussion section to make the whole structure transition more smoothly. We have also refined the subsequent content to ensure a logical and coherent flow, making the entire section conform to formal academic writing standards.

Minor improvements

1. Language issues:

Fix grammar issues that reduce clarity (e.g., “The processed can be defined”, “Without whistles and bells”). Ensure consistent use of terms: uncertainty vs change probability, memory bank vs memory, etc.

**Response**: Thank you for the suggestions. We have thoroughly revised the entire manuscript to fix all grammatical errors and expressions that may affect readability.

In addition, we have standardized the terminology throughout the manuscript to maintain consistency. We have unified the use of “uncertainty” instead of inconsistent mixing with “change probability” and we have maintained a clear and consistent distinction between memory bank (referring to the entire memory module and storage structure) and memory (referring to the stored features and information within the bank) to remain consistent with SAM2.

2. Define all symbols and variables used in the eqs. Use explicit, not implicit sums over pixels.

**Response**: Thank you for this valuable suggestion to improve formula clarity and reproducibility. We have revised all equations in the manuscript to define every symbol and variable explicitly, with clear descriptions provided immediately after each equation or in a dedicated notation list.

In addition, for loss functions and other pixel-level computations, we have replaced implicit summations with explicit spatial summations over the image pixels, making the computation process more transparent and mathematically rigorous. These changes ensure that all formulations are unambiguous and easy to follow.
