# Supplementary material for: UAMP: Consistent video object segmentation with uncertainty-aware memory propagation
Source: PLoS One. 2026 Jul 7;21(7):e0353156. doi: 10.1371/journal.pone.0353156 (PMC13718450; doi:10.1371/journal.pone.0353156)
Supplement: Peer review history

# Peer review: Decision Letter 0

**DOI:** 10.1371/journal.pone.0353156.r001

**Authors:** Taikyeong Ted Jeong

6 Feb 2026

PONE-D-25-54851

UAMP: Consistent Video Object Segmentation with Uncertainty-Aware Memory Propagation

PLOS One

Dear Dr. Liu,

Thank you for submitting your manuscript to PLOS ONE. After careful consideration, we feel that it has merit but does not fully meet PLOS ONE’s publication criteria as it currently stands. Therefore, we invite you to submit a revised version of the manuscript that addresses the points raised during the review process.

==============================

Now, please revise your manuscript based on the comments of the three reviewers. Not only are all the answers clear and concise, but your sincere responses will help develop your manuscript into a valuable academic paper for our journal.

==============================

Please submit your revised manuscript by Mar 23 2026 11:59PM. If you will need more time than this to complete your revisions, please reply to this message or contact the journal office at plosone@plos.org. When you're ready to submit your revision, log on to https://www.editorialmanager.com/pone/ and select the 'Submissions Needing Revision' folder to locate your manuscript file.

Please include the following items when submitting your revised manuscript:

- A letter that responds to each point raised by the academic editor and reviewer(s). You should upload this letter as a separate file labeled 'Response to Reviewers'.
- A marked-up copy of your manuscript that highlights changes made to the original version. You should upload this as a separate file labeled 'Revised Manuscript with Track Changes'.
- An unmarked version of your revised paper without tracked changes. You should upload this as a separate file labeled 'Manuscript'.

If you would like to make changes to your financial disclosure, please include your updated statement in your cover letter. Guidelines for resubmitting your figure files are available below the reviewer comments at the end of this letter.

If applicable, we recommend that you deposit your laboratory protocols in protocols.io to enhance the reproducibility of your results. Protocols.io assigns your protocol its own identifier (DOI) so that it can be cited independently in the future. For instructions see: https://journals.plos.org/plosone/s/submission-guidelines#loc-laboratory-protocols. Additionally, PLOS ONE offers an option for publishing peer-reviewed Lab Protocol articles, which describe protocols hosted on protocols.io. Read more information on sharing protocols at https://plos.org/protocols?utm_medium=editorial-email&utm_source=authorletters&utm_campaign=protocols.

We look forward to receiving your revised manuscript.

Kind regards,

Taikyeong Ted Jeong, Ph.D.

Academic Editor

PLOS One

Journal Requirements:

When submitting your revision, we need you to address these additional requirements.

1. Please ensure that your manuscript meets PLOS ONE's style requirements, including those for file naming. The PLOS ONE style templates can be found at

https://journals.plos.org/plosone/s/file?id=wjVg/PLOSOne_formatting_sample_main_body.pdf and https://journals.plos.org/plosone/s/file?id=ba62/PLOSOne_formatting_sample_title_authors_affiliations.pdf

2. Please note that PLOS One has specific guidelines on code sharing for submissions in which author-generated code underpins the findings in the manuscript. In these cases, we expect all author-generated code to be made available without restrictions upon publication of the work. Please review our guidelines at https://journals.plos.org/plosone/s/materials-and-software-sharing#loc-sharing-code and ensure that your code is shared in a way that follows best practice and facilitates reproducibility and reuse.

3. Thank you for stating the following financial disclosure:

This research was funded by Shaanxi University of Technology, grant number SLGRC202416.

Please state what role the funders took in the study. If the funders had no role, please state: "The funders had no role in study design, data collection and analysis, decision to publish, or preparation of the manuscript."

If this statement is not correct you must amend it as needed.

Please include this amended Role of Funder statement in your cover letter; we will change the online submission form on your behalf.

4. Thank you for stating the following in the Acknowledgments Section of your manuscript:

This research was funded by Shaanxi University of Technology, grant number SLGRC202416.

We note that you have provided funding information that is not currently declared in your Funding Statement. However, funding information should not appear in the Acknowledgments section or other areas of your manuscript. We will only publish funding information present in the Funding Statement section of the online submission form.

Please remove any funding-related text from the manuscript and let us know how you would like to update your Funding Statement. Currently, your Funding Statement reads as follows:

This research was funded by Shaanxi University of Technology, grant number SLGRC202416.

Please include your amended statements within your cover letter; we will change the online submission form on your behalf.

5. We note that Figure(s) 1, 2, 3, 5, 6, 7, 8, 9, 10, 11 in your submission contain copyrighted images. All PLOS content is published under the Creative Commons Attribution License (CC BY 4.0), which means that the manuscript, images, and Supporting Information files will be freely available online, and any third party is permitted to access, download, copy, distribute, and use these materials in any way, even commercially, with proper attribution. For more information, see our copyright guidelines: http://journals.plos.org/plosone/s/licenses-and-copyright.

We require you to either (1) present written permission from the copyright holder to publish these figures specifically under the CC BY 4.0 license, or (2) remove the figures from your submission:

a. You may seek permission from the original copyright holder of Figure(s) 1, 2, 3, 5, 6, 7, 8, 9, 10, 11 to publish the content specifically under the CC BY 4.0 license.

We recommend that you contact the original copyright holder with the Content Permission Form (http://journals.plos.org/plosone/s/file?id=7c09/content-permission-form.pdf) and the following text:

“I request permission for the open-access journal PLOS ONE to publish XXX under the Creative Commons Attribution License (CCAL) CC BY 4.0 (http://creativecommons.org/licenses/by/4.0/). Please be aware that this license allows unrestricted use and distribution, even commercially, by third parties. Please reply and provide explicit written permission to publish XXX under a CC BY license and complete the attached form.”

Please upload the completed Content Permission Form or other proof of granted permissions as an "Other" file with your submission.

In the figure caption of the copyrighted figure, please include the following text: “Reprinted from [ref] under a CC BY license, with permission from [name of publisher], original copyright [original copyright year].”

b. If you are unable to obtain permission from the original copyright holder to publish these figures under the CC BY 4.0 license or if the copyright holder’s requirements are incompatible with the CC BY 4.0 license, please either i) remove the figure or ii) supply a replacement figure that complies with the CC BY 4.0 license. Please check copyright information on all replacement figures and update the figure caption with source information. If applicable, please specify in the figure caption text when a figure is similar but not identical to the original image and is therefore for illustrative purposes only.

6. If the reviewer comments include a recommendation to cite specific previously published works, please review and evaluate these publications to determine whether they are relevant and should be cited. There is no requirement to cite these works unless the editor has indicated otherwise.

Additional Editor Comments :

Please now revise your manuscript based on the comments of the three reviewers. Not only are all the answers clear and concise, but your sincere responses will help develop your manuscript into a valuable academic paper for our journal.

[Note: HTML markup is below. Please do not edit.]

Reviewers' comments:

Reviewer's Responses to Questions

**Comments to the Author**

1. Is the manuscript technically sound, and do the data support the conclusions?

The manuscript must describe a technically sound piece of scientific research with data that supports the conclusions. Experiments must have been conducted rigorously, with appropriate controls, replication, and sample sizes. The conclusions must be drawn appropriately based on the data presented.

Reviewer #1: Yes

Reviewer #2: Partly

Reviewer #3: Yes

**********

2. Has the statistical analysis been performed appropriately and rigorously?

Reviewer #1: Yes

Reviewer #2: I Don't Know

Reviewer #3: Yes

**********

3. Have the authors made all data underlying the findings in their manuscript fully available?

The PLOS Data policy requires authors to make all data underlying the findings described in their manuscript fully available without restriction, with rare exception (please refer to the Data Availability Statement in the manuscript PDF file). The data should be provided as part of the manuscript or its supporting information, or deposited to a public repository. For example, in addition to summary statistics, the data points behind means, medians and variance measures should be available. If there are restrictions on publicly sharing data—e.g. participant privacy or use of data from a third party—those must be specified.

Reviewer #1: Yes

Reviewer #2: Yes

Reviewer #3: Yes

**********

4. Is the manuscript presented in an intelligible fashion and written in standard English?

PLOS ONE does not copyedit accepted manuscripts, so the language in submitted articles must be clear, correct, and unambiguous. Any typographical or grammatical errors should be corrected at revision, so please note any specific errors here.

Reviewer #1: Yes

Reviewer #2: Yes

Reviewer #3: Yes

**********

5. Review Comments to the Author

Please use the space provided to explain your answers to the questions above. You may also include additional comments for the author, including concerns about dual publication, research ethics, or publication ethics. (Please upload your review as an attachment if it exceeds 20,000 characters)

Reviewer #1: 1. The abstract and conclusion need to be improved. The abstract must be a concise yet comprehensive reflection of what is in your paper. Please modify the abstract according to “motivation, description, results and conclusion” parts. I suggest extending the conclusions section to focus on the results you get, the method you propose, and their significance.

2. The Section 1 and Section 2 is too short. I suggest the authors can merge section 1 and section 2.

3. What is the motivation of the proposed method? The details of motivation and innovations are important for potential readers and journals. Please add this detailed description in the last paragraph in section I. Please modify the paragraph according to "For this paper, the main contributions are as follows: (1) ......" to Section I. Please give the details of motivations. In Section 1, I suggest the authors can amend your contributions of manuscript in the last of Section 1.

4. The description of manuscript is very important for potential reader and other researchers. I encourage the authors to have their manuscript proof-edited by a native English speaker to enhance the level of paper presentation. There are some occasional grammatical problems within the text. It may need the attention of someone fluent in English language to enhance the readability.

5. The introduction section of the paper needs to revise according to the timeline of technology development. Please update references with recent paper in CVPR, ICCV, ECCV et al and Elsevier, Springer. In your section 1 and section 2, I suggest the authors amend several related literatures and corresponding references in recent years. For example: Dual Degradation Image Inpainting Method via Adaptive Feature Fusion and U-Net Network (Applied Soft Computing); CAAT: Image Super-resolution Algorithm via Channel Attention and Transformer (Array); MGNet: RGBT tracking via cross-modality cross-region mutual guidance (Neural Networks); Crack segmentation network via difference convolution-based encoder and hybrid CNN-Mamba multi-scale attention (Pattern Recognition)

6. Please give the details of proposed method for proposed model. I suggest the authors amend the calculation of your size of proposed method and the details is important for proposed method.

7. The content of experiments needs to amend related experiments to compare related SOTA in recent three years. I recommend the authors amend related experimental results of proposed method of SOTA according to the published paper in IEEE, Springer and Elsevier.

8. However, the manuscript, in its present form, contains several weaknesses. Adequate revisions to the following points should be undertaken in order to justify recommendation for publication.

9. In the conclusion section, the limitations of this study and suggested improvements of this work should be highlighted.

10. Provide a critical review of the previous "journal" (not conference) papers in the area and explain the inadequacies of previous approaches.

11. I suggest the authors revise Section 1 and Section 2. Please revise the content according to the development of timeline.

12. Please check all parameters in the manuscript and amend some related description of primary parameters. In section 3, please write the proposed algorithm in a proper algorithm/pseudocode format with section 3. Otherwise, it is very hard to follow. Some examples here: https://tex.stackexchange.com/questions/204592/how-to-format-a-pseudocode-algorithm

Reviewer #2: 1. UAMP uses long-term and short-term memory. How is the balance between these two memories determined and is it adaptable for different videos?

2- J&F increase of up to 5.6 has been reported. Is this improvement the same for all object classes or only for specific motion patterns?

3-The goal of the UAMP paper is clear, but how does this model ensure that the combination of uncertainty and dual memory in long videos and crowded scenes actually provides stable performance?

4-Most studies focus on short-term or long-term memory, but how can one strike an optimal balance between the two for long videos and crowded scenes?

5-Motion- or appearance-based models each have their limitations, but is there an approach that simultaneously exploits uncertainty in motion and appearance to improve coherence?

6- Previous methods such as XMem and SAM2Long have complex and expensive memory, but how can high performance be maintained at a reasonable computational cost?

7-Most studies have focused on single or simple objects, but how can one solve VOS in a stable manner in crowded scenes with similar and hidden objects?

8- How does Figure 2 relate to Relationship 1?

9-In long-term memory design, how is it determined how much of the old memory should be forgotten to both maintain performance and reduce computational cost?

10- Given the higher volatility of the motion score than the object score, how do the authors ensure that the weight chosen is optimal for combining motion and appearance uncertainty and is stable under different conditions (occlusion and reappearance of objects)?

Reviewer #3: The whole article is properly written understandably. Moreover, this article sounds well with various aspects in this research area and the involvement of this work is appreciable.

The author seeks out the problems that need creative thinking in this demand area and delivers a superior end result with a detailed description.

All comments and recommendations have been addressed, and the paper has been significantly improved.

**********

6. PLOS authors have the option to publish the peer review history of their article (what does this mean?). If published, this will include your full peer review and any attached files.

If you choose “no”, your identity will remain anonymous but your review may still be made public.

**Do you want your identity to be public for this peer review?**  For information about this choice, including consent withdrawal, please see our Privacy Policy.

Reviewer #1: No

Reviewer #2: No

Reviewer #3: **Yes:** Dr Santhosh Kumar Balan

**********

[NOTE: If reviewer comments were submitted as an attachment file, they will be attached to this email and accessible via the submission site. Please log into your account, locate the manuscript record, and check for the action link "View Attachments". If this link does not appear, there are no attachment files.]

To ensure your figures meet our technical requirements, please review our figure guidelines: https://journals.plos.org/plosone/s/figures

You may also use PLOS’s free figure tool, NAAS, to help you prepare publication quality figures: https://journals.plos.org/plosone/s/figures#loc-tools-for-figure-preparation.

NAAS will assess whether your figures meet our technical requirements by comparing each figure against our figure specifications.

# Author comment: Author response to Decision Letter 1

**DOI:** 10.1371/journal.pone.0353156.r002

25 Feb 2026

For academic editor:

1. Please ensure that your manuscript meets PLOS ONE's style requirements, including those for file naming.

Response: We thank the editor for the reminder. We have carefully checked and revised our manuscript to ensure full compliance with PLOS ONE’s style and formatting requirements, including the file naming conventions and all other journal specifications. All necessary adjustments have been made accordingly.

2. Please note that PLOS One has specific guidelines on code sharing for submissions in which author-generated code underpins the findings in the manuscript.

Response: We agree with the suggestion on making the code available. At present, we provided an initial version of the code for review and verification purpose, with the url: https://github.com/Feynluo/uamp-sam2 . We will further organize, refine, and fully release the complete code on a public repository upon formal acceptance of the manuscript.

3. Thank you for stating the following financial disclosure: This research was funded by Shaanxi University of Technology, grant number SLGRC202416. Please state what role the funders took in the study.

Response: The funder (Shaanxi University of Technology, grant number SLGRC202416) provided financial support only for this study. The funder had no role in the design of the study, data collection, data analysis, interpretation of results, writing of the manuscript, or decision to publish. And the role of funder statement is amended in the cover letter.

4. Thank you for stating the following in the Acknowledgments Section of your manuscript: This research was funded by Shaanxi University of Technology, grant number SLGRC202416. Please remove any funding-related text from the manuscript and let us know how you would like to update your Funding Statement. Please include your amended statements within your cover letter; we will change the online submission form on your behalf.

Response: We have removed the funding-related text from the Acknowledgments section, and the funding statement: This research was funded by Shaanxi University of Technology, grant number SLGRC202416, is included in the cover letter.

5. We note that Figure(s) 1, 2, 3, 5, 6, 7, 8, 9, 10, 11 in your submission contain copyrighted images. All PLOS content is published under the Creative Commons Attribution License (CC BY 4.0), which means that the manuscript, images, and Supporting Information files will be freely available online, and any third party is permitted to access, download, copy, distribute, and use these materials in any way, even commercially, with proper attribution. For more information, see our copyright guidelines: http://journals.plos.org/plosone/s/licenses-and-copyright.

Response: We sincerely appreciate the reminder concerning the copyright and licensing issues of the figures in our manuscript.

We hereby confirm that all figures included in this manuscript are original works created by the authors and do not involve any third-party materials, copyrighted content, or previously published images, and none of the figures pose conflicts with the CC BY 4.0 licensing terms.

And we provide the original figures created with draw.io tool in the supplement materials.

6. If the reviewer comments include a recommendation to cite specific previously published works, please review and evaluate these publications to determine whether they are relevant and should be cited. There is no requirement to cite these works unless the editor has indicated otherwise.

Response: We thank the editor for the reminder concerning the citations suggested by the reviewers. We have carefully reviewed and evaluated all the recommended publications for their relevance to the present study. Those that are relevant and provide valuable support to our work have been appropriately cited in the revised manuscript. In accordance with the editorial guidance, we have not cited publications that are not directly relevant to the content of this paper.

Reviewer #1:

1. The abstract and conclusion need to be improved. The abstract must be a concise yet comprehensive reflection of what is in your paper. Please modify the abstract according to “motivation, description, results and conclusion” parts. I suggest extending the conclusions section to focus on the results you get, the method you propose, and their significance.

Response: We sincerely appreciate the valuable comments.

The abstract has been thoroughly revised to concisely and comprehensively reflect the manuscript, following the "motivation, description, results and conclusion" structure. The motivation clarifies SAM 2’s limitations in long-term VOS (error accumulation, poor occlusion robustness, inadequate adaptation to dynamic objects). The description details our proposed UAMP (a SAM 2 variant) and its core uncertainty-aware memory propagation framework with three key components. The results present key experimental findings, including UAMP’s superior performance across five VOS benchmarks, a maximum 5.6-point J&F improvement over SOTA methods, and stable performance in complex scenarios. The abstract’s conclusion summarizes UAMP’s effectiveness in enhancing SAM 2 for practical VOS.

The conclusion has been extended and optimized to focus on the proposed method, results, and their significance. We clarified UAMP’s core innovation and its targeted design to address SAM 2’s limitations. Key experimental results are elaborated, confirming UAMP’s effectiveness in mitigating error accumulation and adapting to dynamic objects, which provides a reusable module for VOS.

All revisions have been made in the revision.

2. The Section 1 and Section 2 is too short. I suggest the authors can merge section 1 and section 2.

Response: Thanks for your valuable suggestions. Section 1 (Introduction) focuses on the research background, SAM 2’s limitations in long-term VOS, the motivation of our UAMP method, and a brief overview of its core ideas, aiming to clarify the research gap and the necessity of our study. In contrast, Section 2 (Related Works) provides the related research, summarizing the development of VOS, and different paradigms, including the Memory-based VOS and Consistency Modeling, and highlighting the differences between these methods and our UAMP, helping readers grasp current research progress and our study’s innovation.

And further, according the following advices of 3 and 5, we further improve the introduction and related works section in the revision.

3. What is the motivation of the proposed method? The details of motivation and innovations are important for potential readers and journals. Please add this detailed description in the last paragraph in section I. Please modify the paragraph according to "For this paper, the main contributions are as follows: (1) ......" to Section I. Please give the details of motivations. In Section 1, I suggest the authors can amend your contributions of manuscript in the last of Section 1.

Response: Thanks for the suggestions. First, detailed descriptions of the motivation for the proposed UAMP method have been added to the last paragraph of Section I. The expanded motivation clarifies the research background (the wide application of SAM 2 as a foundational VOS model) and elaborates on the critical limitations of SAM 2 in long-term VOS tasks—specifically, error accumulation caused by its greedy-selection memory architecture, insufficient robustness to object occlusions and reappearances, and inadequate adaptation to dynamic appearance and motion variations of objects in complex scenarios.

Second, the contributions of the manuscript have been revised and placed at the end of Section I, following the format suggested by the reviewer: "For this paper, the main contributions are as follows: In summary, our main contributions are as follows: 1) We propose UAMP, a consistent video object segmentation model, which is specifically designed to address the inherent lim-itations of SAM 2 in long-term VOS tasks, with a core uncertainty-aware memory propagation framework, providing a reusable module for optimizing memory-based video segmentation models. 2) We introduce an uncertainty-aware memory propagation mechanism via re-gion-adaptive memory fusion by appearance and motion uncertainty estimation to refine the feature representation. At the meantime, a long-term memory updating mechanism and score-based short-term memory selection is designed to reduce the disturbance in crowded scenes by a mixture of motion and affinity scores. 3) Our UAMP achieves state-of-the-art performance on SA-V, MOSE, LVOSv1 and LVOSv2, and other VOT benchmarks, demonstrating effectiveness across diverse datasets".

4. The description of manuscript is very important for potential reader and other researchers. I encourage the authors to have their manuscript proof-edited by a native English speaker to enhance the level of paper presentation. There are some occasional grammatical problems within the text. It may need the attention of someone fluent in English language to enhance the readability.

Response: Thanks for the valuable suggestion regarding the manuscript’s presentation and English language readability. Specifically, we have invited a native English speaker with expertise in academic writing (focusing on computer vision and machine learning fields) to conduct a comprehensive proof-editing of the entire manuscript. And the proof-editing work has been completed in the revised manuscript.

5. The introduction section of the paper needs to revise according to the timeline of technology development. Please update references with recent paper in CVPR, ICCV, ECCV et al and Elsevier, Springer. In your section 1 and section 2, I suggest the authors amend several related literatures and corresponding references in recent years. For example: Dual Degradation Image Inpainting Method via Adaptive Feature Fusion and U-Net Network (Applied Soft Computing); CAAT: Image Super-resolution Algorithm via Channel Attention and Transformer (Array); MGNet: RGBT tracking via cross-modality cross-region mutual guidance (Neural Networks); Crack segmentation network via difference convolution-based encoder and hybrid CNN-Mamba multi-scale attention (Pattern Recognition)

Response: Thanks for the valuable advice. And the introduction section of the paper has been revised according to the timeline of technology development with the updated recent papers. Further, in section 1 and section 2, new related literatures have been amended in the revision.

Specifically, “SAM2Long [4] aimed to address the failure of SAM 2’s FIFO fixed-window memory mechanism in handling target reappearance by introducing a hierarchical memory tree structure for flexible memory retrieval and management, though it suffered from increased memory overhead, potential retrieval latency, and failed to resolve the confusion between simi-lar-looking instances during memory retrieval. SAMURAI [3] integrated Kalman filtering into SAM 2’s memory update process to model target motion trajectories and suppress error ac-cumulation through a prediction-update mechanism, yet its performance was heavily de-pendent on motion prediction accuracy in fast-changing scenarios, and it could not effectively handle severe target deformation that led to large prediction errors. DAM4SAM [5], proposes a novel distractor-aware memory (DAM) module for the SAM 2 model, which is composed of recent appearance memory (RAM) and designed to address the interference of distractor objects (irrelevant objects with similar appearance to the target) in VOS tasks. However, DAM4SAM over-relies on the recent appearance features stored in RAM, making it less adaptable to scenarios where the target undergoes significant appearance changes over time. DC-SAM [45] leverages multi-source features and generates positive and negative prompts by ensuring prompt consistency, integrating with SAM/SAM2 to achieve in-context segmentation for both images and videos. and improves the model's adaptability to complex scenes. But it focuses on prompt-tuning adaption, and lacks effective long-term temporal correlation modeling capabilities, making it difficult to maintain consistent segmentation performance when the target undergoes continuous appearance changes (such as scale variation, deformation) in long video sequences”.

6. Please give the details of proposed method for proposed model. I suggest the authors amend the calculation of your size of proposed method and the details is important for proposed method.

Response: We sincerely appreciate your valuable comment. Following your suggestion, we have supplemented the detailed implementation and dimensional calculation of each core module of the proposed model, and revised the relevant content in the section 3 and section 4.12 of model details.

Specifically, “For image encoder, the input image is first scaled to the resolution of 1024, and the image embeddings are obtained by fusing the stride 16 and 32 features from Stages 3 and 4 of the Hiera [25] image encoder with the dimension 448×64×64 and 896×32×32 respectively. These multi-scale features are then fused via a pyramid network following [1], and model dimension of Hiera is 256. The positional embeddings are calculated with the windowed absolute positional embeddings, and the positional information spanning across windows is obtained by interpolating the positional embeddings. For memory attention, the input feature map is the 256×64×64 embeddings generated by the memory encoder, the 2d spatial Rotary Positional Embedding (RoPE) [30] with the dimension of 256 is utilized in both self-attention and cross-attention layers, and this module composes 4 layers. For mask decoder, the stride 4 and 8 features from Stages 1 and 2 are added to the up-sampling layers to get high-resolution features. Following [6], the appearance uncertainty prediction module comprises one 1×1 convolution and two 3×3 convolutions. It takes the input of a concatenation of current frame feature, last frame feature, and last mask prediction, and outputs a one-channel change probability mask of the query token. For memory bank, the memory features 256×64×64 are projected to the dimension of 2048 for 8 memory banks via a linear layer, and the short-term memory selection is designed follows DAM [5] as discussed in Section 3.3. The long-term memory updating composes 4 layers of the transformer-like structure, in which the self-attention layer is replaced with the linear-attention layer. And the matrix state is projected to the same dimension as short-term memory with a linear projection layer.”

7. The content of experiments needs to amend related experiments to compare related SOTA in recent three years. I recommend the authors amend related experimental results of proposed method of SOTA according to the published paper in IEEE, Springer and Elsevier.

Response: We sincerely appreciate your comment. We have amended related experimental results with the state-of-the-art (SOTA) methods in the field published in the past three years in the Table 1 and Table 2 of section 4.2.

Specifically, we compare with the Cutie-bas [20] method presented at CVPR 2024, LiVOS [46] at CVPR 2025, SAM2.1[1] at ICLR 2025, and SAM2.1Long [4] at ICCV 2025. All the selected approaches provide complete open-source implementations or detailed experimental protocols in their original papers, which ensures the fairness of our comparative evaluation.

8. However, the manuscript, in its present form, contains several weaknesses. Adequate revisions to the following points should be undertaken in order to justify recommendation for publication.

Response: We sincerely appreciate the reviewer for the constructive comments and valuable suggestions. We fully agree that the manuscript needs further improvement to enhance its quality. We have carefully revised the manuscript according to the comments, and the detailed revisions are addressed point by point in the revision.

9. In the conclusion section, the limitations of this study and suggested improvements of this work should be highlighted.

Response: We sincerely appreciate the reviewer’s constructive suggestion. Following this comment, we have supplemente

Attachment Submitted filename: Response to Reviewers.docx

# Peer review: Decision Letter 1

**DOI:** 10.1371/journal.pone.0353156.r003

**Authors:** Taikyeong Ted Jeong

15 Mar 2026

PONE-D-25-54851R1

UAMP: Consistent Video Object Segmentation with Uncertainty-Aware Memory Propagation

PLOS One

Dear Dr. Liu,

Thank you for submitting your manuscript to PLOS ONE. After careful consideration, we feel that it has merit but does not fully meet PLOS ONE’s publication criteria as it currently stands. Therefore, we invite you to submit a revised version of the manuscript that addresses the points raised during the review process.

==============================

After reviewing the revised manuscript and the reviewers’ comments, I agree that the study presents a technically sound approach for improving semi-supervised video object segmentation using an uncertainty-aware memory propagation strategy. The proposed framework integrates appearance-based uncertainty estimation, motion-based scoring, and dual memory mechanisms to address limitations in existing memory update strategies. The reviewers generally agree that the manuscript has improved compared with the previous version and that the overall approach is technically meaningful.

However, several issues remain that should be addressed before the manuscript can be considered for acceptance.

**Required revisions**

1. **Clarification and formal definition of uncertainty modules**
  The manuscript should clearly define the appearance and motion uncertainty components and describe how these quantities are supervised or learned during training. The current description remains partly conceptual and does not fully specify the mathematical formulation.
2. **Reproducibility of the proposed framework**
  Several key elements of the method require clearer specification to ensure reproducibility, including:

- the gating mechanism used for balancing long-term and short-term memory
- the exact formulation of the uncertainty estimation modules
- the loss functions and weighting schemes used during training
- the full specification of the Kalman filter used for motion scoring
- **Algorithmic clarity and symbol definitions**
  All variables and symbols appearing in the equations should be explicitly defined. The pseudocode and equations should be sufficiently detailed to allow independent implementation.
- **Experimental clarity and evaluation reporting**
  The evaluation section would benefit from clearer reporting of experimental settings, including hyperparameters, memory bank configuration, and training schedules. Where possible, provide additional breakdown of performance (e.g., per-category or scenario-level results) to support the claims regarding improved robustness in challenging scenarios such as occlusion or crowded scenes.
- **Data availability statement**
  One reviewer indicated concerns regarding the availability of underlying data. Please ensure that all data required to reproduce the reported findings are publicly available or clearly described in accordance with PLOS ONE’s data availability policy.

**Recommended improvements**

1. Improve the structure and clarity of section introductions so that each section and subsection begins with a brief explanation of its purpose.
2. Ensure consistent terminology throughout the manuscript (e.g., “uncertainty”, “change probability”, “memory bank”, etc.).
3. Provide a brief discussion of computational overhead and inference latency compared with the baseline SAM2 framework.
4. Review the suggested references provided by the reviewers and cite them where appropriate.

Overall, the reviewers consider the manuscript technically sound, but the issues listed above should be addressed to ensure clarity and reproducibility consistent with PLOS ONE’s publication criteria.

==============================

Please submit your revised manuscript by Apr 29 2026 11:59PM. If you will need more time than this to complete your revisions, please reply to this message or contact the journal office at plosone@plos.org. When you're ready to submit your revision, log on to https://www.editorialmanager.com/pone/ and select the 'Submissions Needing Revision' folder to locate your manuscript file.

Please include the following items when submitting your revised manuscript:

- A letter that responds to each point raised by the academic editor and reviewer(s). You should upload this letter as a separate file labeled 'Response to Reviewers'.
- A marked-up copy of your manuscript that highlights changes made to the original version. You should upload this as a separate file labeled 'Revised Manuscript with Track Changes'.
- An unmarked version of your revised paper without tracked changes. You should upload this as a separate file labeled 'Manuscript'.

If you would like to make changes to your financial disclosure, please include your updated statement in your cover letter. Guidelines for resubmitting your figure files are available below the reviewer comments at the end of this letter.

If applicable, we recommend that you deposit your laboratory protocols in protocols.io to enhance the reproducibility of your results. Protocols.io assigns your protocol its own identifier (DOI) so that it can be cited independently in the future. For instructions see: https://journals.plos.org/plosone/s/submission-guidelines#loc-laboratory-protocols. Additionally, PLOS ONE offers an option for publishing peer-reviewed Lab Protocol articles, which describe protocols hosted on protocols.io. Read more information on sharing protocols at https://plos.org/protocols?utm_medium=editorial-email&utm_source=authorletters&utm_campaign=protocols.

We look forward to receiving your revised manuscript.

Kind regards,

Taikyeong Ted Jeong, Ph.D.

Academic Editor

PLOS One

Journal Requirements:

If the reviewer comments include a recommendation to cite specific previously published works, please review and evaluate these publications to determine whether they are relevant and should be cited. There is no requirement to cite these works unless the editor has indicated otherwise.

[Note: HTML markup is below. Please do not edit.]

Reviewers' comments:

Reviewer's Responses to Questions

**Comments to the Author**

1. If the authors have adequately addressed your comments raised in a previous round of review and you feel that this manuscript is now acceptable for publication, you may indicate that here to bypass the “Comments to the Author” section, enter your conflict of interest statement in the “Confidential to Editor” section, and submit your "Accept" recommendation.

Reviewer #4: (No Response)

Reviewer #5: All comments have been addressed

Reviewer #6: All comments have been addressed

**********

2. Is the manuscript technically sound, and do the data support the conclusions?

The manuscript must describe a technically sound piece of scientific research with data that supports the conclusions. Experiments must have been conducted rigorously, with appropriate controls, replication, and sample sizes. The conclusions must be drawn appropriately based on the data presented.

Reviewer #4: Yes

Reviewer #5: Yes

Reviewer #6: Partly

**********

3. Has the statistical analysis been performed appropriately and rigorously?

Reviewer #4: Yes

Reviewer #5: Yes

Reviewer #6: I Don't Know

**********

4. Have the authors made all data underlying the findings in their manuscript fully available?

The PLOS Data policy requires authors to make all data underlying the findings described in their manuscript fully available without restriction, with rare exception (please refer to the Data Availability Statement in the manuscript PDF file). The data should be provided as part of the manuscript or its supporting information, or deposited to a public repository. For example, in addition to summary statistics, the data points behind means, medians and variance measures should be available. If there are restrictions on publicly sharing data—e.g. participant privacy or use of data from a third party—those must be specified.

Reviewer #4: Yes

Reviewer #5: Yes

Reviewer #6: No

**********

5. Is the manuscript presented in an intelligible fashion and written in standard English?

PLOS ONE does not copyedit accepted manuscripts, so the language in submitted articles must be clear, correct, and unambiguous. Any typographical or grammatical errors should be corrected at revision, so please note any specific errors here.

Reviewer #4: Yes

Reviewer #5: Yes

Reviewer #6: Yes

**********

6. Review Comments to the Author

Please use the space provided to explain your answers to the questions above. You may also include additional comments for the author, including concerns about dual publication, research ethics, or publication ethics. (Please upload your review as an attachment if it exceeds 20,000 characters)

Reviewer #4: 1. The pseudocode in Section 3 is a good addition, but some steps remain underspecified. For example:

a. The gating mechanism for balancing long-term and short-term memory is described qualitatively, but the exact mathematical formulation (activation function, normalization strategy) is missing.

b. The uncertainty estimation modules (appearance vs. motion) are introduced, but the training loss functions and weighting schemes are not fully detailed.

2. The author(s) introduces additional modules (uncertainty prediction, dual memory mechanisms), but there is no discussion of computational overhead. How does UAMP scale in terms of memory footprint and inference latency compared to SAM2?

3. Although recent SOTA methods (Cutie-bas, LiVOS, SAM2.1, SAM2.1Long) are included, the evaluation could be expanded: Report per-class or per-category breakdowns of J&F improvements to substantiate claims that UAMP excels in crowded or occluded scenarios.

4. The manuscript alternates between “uncertainty-aware memory propagation” and “region-adaptive fusion.” It would help to standardize terminology to avoid confusion.

5. Section 4 mentions dimensional calculations, but hyperparameter choices (e.g., number of memory banks, learning rate schedules) should be explicitly tabulated for clarity.

6. Some related works are recommended for citation:

a. https://doi.org/10.3390/s23125565

b. https://doi.org/10.1109/PESA.2015.7398965

c. https://doi.org/10.3109/10715762.2012.721928

d. https://doi.org/10.1016/j.mee.2010.11.019

Reviewer #5: All the coments form the reviewers are addressed. How ever I noticed that subsections are directly strated without any information about that section example section 3, 3.1 is directly written there is no infrmation or description. Same thing is repited in next section and its subsection. This is not a professional way of teachical writing.

Reviewer #6: The revised manuscript proposes UAMP, an enhanced SAM 2 variant for semi-supervised video object segmentation (VOS) that targets failure modes in crowded scenes, fast motion, self-occlusion, and long occlusions. The core claim is that SAM 2’s greedy memory update can lead to error accumulation, and that improved consistency requires (i) appearance “uncertainty” (change probability)–guided feature fusion, (ii) Kalman-filter–based motion scoring for mask selection, and (iii) dual memory management: long-term memory updating via linear attention with a learnable forget gate, and short-term memory selection based on a hybrid score system.

The novelty is mainly in the combination and engineering of several known components into a single pipeline. The manuscript would read as more original if it (a) formalized the “uncertainty” concepts more rigorously and (b) clarified exactly what is new relative to MatAnyone-style fusion + SAMURAI motion scoring + DAM-style selection.

Several key parts remain underspecified or internally inconsistent, which limits reproducibility and makes it hard to judge whether the gains come from the proposed mechanisms or from evaluation/training choices.

Major Improvements

1) Define “uncertainty” and its supervsion precisely (appearance and motion)

2) Assure all symbols in the eqs are defined and self-consistent

3) Provide full Kalman filter details (reproducibility)

4) Short-term memory selection rule seems underspecified (Eq. 12)

5) Long-term memory updating needs clearer integration with SAM 2 memory bank

6) TheDiscussion section currently starts abruptly; it needs improvement to following the scientific writing standards

Minor improvements

1. Language issues:

Fix grammar issues that reduce clarity (e.g., “The processed can be defined”, “Without whistles and bells”). Ensure consistent use of terms: uncertainty vs change probability, memory bank vs memory, etc.

2. Define all symbols and variables used in the eqs. Use explicit, not implicit sums over pixels.

**********

7. PLOS authors have the option to publish the peer review history of their article (what does this mean?). If published, this will include your full peer review and any attached files.

If you choose “no”, your identity will remain anonymous but your review may still be made public.

**Do you want your identity to be public for this peer review?**  For information about this choice, including consent withdrawal, please see our Privacy Policy.

Reviewer #4: No

Reviewer #5: No

Reviewer #6: No

**********

[NOTE: If reviewer comments were submitted as an attachment file, they will be attached to this email and accessible via the submission site. Please log into your account, locate the manuscript record, and check for the action link "View Attachments". If this link does not appear, there are no attachment files.]

To ensure your figures meet our technical requirements, please review our figure guidelines: https://journals.plos.org/plosone/s/figures

You may also use PLOS’s free figure tool, NAAS, to help you prepare publication quality figures: https://journals.plos.org/plosone/s/figures#loc-tools-for-figure-preparation.

NAAS will assess whether your figures meet our technical requirements by comparing each figure against our figure specifications.

# Author comment: Author response to Decision Letter 2

**DOI:** 10.1371/journal.pone.0353156.r004

15 May 2026

For Editor:

Required revisions:

1) Clarification and formal definition of uncertainty modules

The manuscript should clearly define the appearance and motion uncertainty components and describe how these quantities are supervised or learned during training. The current description remains partly conceptual and does not fully specify the mathematical formulation.

Response: Thank you for your careful and constructive comment. We have further strengthened the mathematical definition and learning mechanism of appearance uncertainty and motion uncertainty to avoid conceptual ambiguity.

Specifically, we have clearly defined the formulation of appearance uncertainty in Eq (5) and its corresponding supervised loss in Eq (6), which explicitly guides the learning of appearance reliability during training. For motion uncertainty, we have provided a precise mathematical formulation in Eq (8-9). In the revised manuscript, we have also added detailed explanations for each component, clarifying how these uncertainty values are modeled, optimized, and integrated into the overall training pipeline. All components are now fully specified with explicit mathematical expressions, ensuring the description is concrete and reproducible.

2) Reproducibility of the proposed framework

Several key elements of the method require clearer specification to ensure reproducibility, including:

a) the gating mechanism used for balancing long-term and short-term memory

b) the exact formulation of the uncertainty estimation modules

c) the loss functions and weighting schemes used during training

d) the full specification of the Kalman filter used for motion scoring

Response: Thank you for your valuable comment on ensuring the reproducibility of our method. We have carefully addressed each of the key elements highlighted, with detailed specifications and explicit formulations supplemented throughout the revised manuscript:

For a) We have mathematically formalized this mechanism in Eq (14), Eq (15), and Eq. (16). Specifically, Eq. (14) clearly defines the specific normalization strategies for the query and key components, and we have provided thorough, step-by-step explanations in the accompanying paragraphs to clarify how the gating weights in Eq (16) are computed.

For b) The formulations for appearance uncertainty and motion uncertainty estimation are explicitly presented in Eq (5) and Eq (9), respectively. We have added detailed definitions of all variables and symbols in these equations, ensuring the mathematical formulation of each uncertainty module is fully specified—no conceptual ambiguity remains, and the implementation logic is clearly elaborated.

For c) The training loss functions corresponding to the uncertainty estimation modules are detailed in Eq (6) and Eq (18). Additionally, the weighting schemes that integrate these uncertainty values into the overall framework are explicitly defined in Eq (4) and Eq (10), with clear descriptions of how these weights are applied during training to optimize the model.

For d) We have supplemented comprehensive Kalman filter formulations for motion uncertainty estimation in Eq (8) and Eq (9), along with clear explanations of all symbols and variables in Section 3.3.2. Furthermore, we have provided the exact definitions of the state transition matrix, measurement matrix, process noise covariance, observation noise covariance, and initial error covariance matrix, along with their specific experimental values to fully support the reproducibility of the motion uncertainty estimation module.

In addition, the pseudocode in Section 3 has been updated accordingly to incorporate all the above specifications, ensuring every key step of the proposed method is fully detailed and can be implemented.

3) Algorithmic clarity and symbol definitions

All variables and symbols appearing in the equations should be explicitly defined. The pseudocode and equations should be sufficiently detailed to allow independent implementation.

Response: Thank you for your valuable comment. We have revised the entire manuscript to explicitly define every variable, symbol, and subscript in all equations, with clear descriptions immediately following each formula in Section 3.

In addition, we have further expanded the pseudocode and mathematical formulations with step-by-step implementation details, including parameter settings, matrix dimensions, iterative procedures, and computation flows in Algorithm 1 and Section 4 model and train details. All critical components are described with sufficient specificity to enable independent re-implementation of the proposed method in the manuscript.

4) Experimental clarity and evaluation reporting

The evaluation section would benefit from clearer reporting of experimental settings, including hyperparameters, memory bank configuration, and training schedules. Where possible, provide additional breakdown of performance (e.g., per-category or scenario-level results) to support the claims regarding improved robustness in challenging scenarios such as occlusion or crowded scenes.

Response: Thank you for your valuable comment. We have detailed a dedicated table that explicitly summarizes all key hyperparameters, including the number of memory banks, learning rate schedule, batch size, optimizer settings, and other related configurations in Table 1 and explained the train details part in Section 4.1.2.

About providing the additional performance, we sincerely appreciate the constructive suggestion on providing more detailed breakdowns to further verify the advantages of UAMP in crowded and occluded scenarios. However, we would like to clarify that the semi-supervised VOS is inherently a class-agnostic task, where given an initial mask, bounding box, or interactive clicks in the first frame, the model only needs to separate the specified target from the background, without relying on semantic category labels. Secondly, none of the compared SOTA methods (Cutie-bas, LiVOS, SAM2.1, SAM2.1Long) reports per-class or per-category J&F metrics in their original papers or official implementations. The official evaluation protocols of the benchmark datasets (e.g., LVOS) also do not provide pre-defined class/category splits for computing such fine-grained per-class statistics. Manually defining arbitrary categories and computing breakdowns alone would lead to incomparable and non-reproducible results against existing SOTAs.

To substantiate our claim that UAMP performs favorably in crowded and occluded scenarios, we evaluated the proposed method on six different datasets, including MOSE, SA-V, LVOSv1, LVOSv2, DAVIS and YouTube-VOS, in which the LVOS is designed for long-term video object segmentation, MOSE, the complex real-life dataset, contains the crowded and occluded scenarios, and SA-V has small, occluded, and reappearing objects in the video sequences. Following the standard evaluation protocol widely accepted in the VOS field, we report overall J&F scores in line with representative recent works. To better substantiate our claim that UAMP performs favorably in crowded and occluded scenarios, we have done the qualitative and quantitative analysis on challenging sequences, highlighting the advantages of our method under complex conditions in Section 4.2.

5) Data availability statement

One reviewer indicated concerns regarding the availability of underlying data. Please ensure that all data required to reproduce the reported findings are publicly available or clearly described in accordance with PLOS ONE’s data availability policy.

Response: Thank you for your reminder regarding the data availability policy. We fully comply with the requirements of PLOS ONE and confirm that all datasets used in this study are publicly accessible standard benchmarks, including MOSE, SA-V, LVOS, DAVIS, and YouTube-VOS, which are widely adopted in the video object segmentation community and freely available to the research community, and download sources are provided in the data availability statement.

We mixed 1% SA-V dataset, 2% MOSE dataset and 1% LVOS dataset for the training dataset, specifically:

1) The SA-V dataset is composed of the videos from sav_000001 to sav_000099 of the sav_000.tar,

2�The MOSE dataset is composed of the videos 10552818 to

Recommended improvements:

1) Improve the structure and clarity of section introductions so that each section and subsection begins with a brief explanation of its purpose.

Response: Thanks for this valuable comment. In the revised version, we have added concise introductory paragraphs at the beginning of the main section to briefly outline the purpose, content, or the organization of the following subsections. These short introductions help guide readers and improve the logical flow of the manuscript in Section 3. We have applied this revision across all relevant sections and subsections to ensure a formal and professional writing style.

2) Ensure consistent terminology throughout the manuscript (e.g., “uncertainty”, “change probability”, “memory bank”, etc.).

Response: Thanks for this valuable comment. We have standardized the terminology throughout the manuscript to maintain consistency. We have unified the use of “uncertainty” instead of inconsistent mixing with “change probability” and we have maintained a clear and consistent distinction between memory bank (referring to the entire memory module and storage structure) and memory (referring to the stored features and information within the bank) to remain consistent with SAM2.

3) Provide a brief discussion of computational overhead and inference latency compared with the baseline SAM2 framework.

Response: Thanks for this valuable comment. We quantitatively compare the proposed UAMP framework with the baseline SAM2 model in terms of memory footprint and inference latency, and analyze the computational complexity and scalability of our method. The corresponding runtime analysis have been added in Section 4.2.2 to clearly demonstrate the efficiency and practicality of UAMP while maintaining performance improvement, which is detailed in Table 9.

4) Review the suggested references provided by the reviewers and cite them where appropriate.

Response: We appreciate the suggested references for potential citation. We have carefully reviewed the research focus of each recommended work:

Paper a) centers on multi-modal feature fusion within a Transformer architecture for integrating visual and audio information to advance dense video captioning. Paper b) explores the design and technological roadmap for developing zero‑emission electric vessels. Paper c) aims at investigating the effect of cigarette smoke exposure on the systemic circulation and local airway 5-HT levels as well as MAO-mediated oxidative pathway using a cigarette smoke-exposed rat model. Paper d) presents device-level simulation and analysis of In₀.₅₃Ga₀.₄₇As implant‑free quantum‑well structures in microelectronics.

Our work targets video object segmentation (VOS), a core task in video understanding that aims to achieve pixel‑level foreground object separation and consistent tracking across consecutive video frames. Given that the above references belong to different research domains, including video captioning, marine engineering, biomedicine, and microelectronic device design, they are not thematically or methodologically aligned with our focus on video object segmentation. Accordingly, we respectfully consider these references less relevant to the present study. We will continue to enrich our literature review by incorporating more state‑of‑the‑art and representative studies dedicated to video object segmentation to better contextualize our contributions for future work.

Reviewer #4

1). The pseudocode in Section 3 is a good addition, but some steps remain underspecified. For example:

a. The gating mechanism for balancing long-term and short-term memory is described qualitatively, but the exact mathematical formulation (activation function, normalization strategy) is missing.

b. The uncertainty estimation modules (appearance vs. motion) are introduced, but the training loss functions and weighting schemes are not fully detailed.

Response: Thanks for your constructive and valuable comments. We have further refined the relevant descriptions and mathematical formulations to clarify the underspecified details in the pseudocode.

Specifically, we have updated the uncertainty estimation modules in Sections 3.3.1 and 3.3.2. The formulations for appearance uncertainty and motion uncertainty estimation are clearly presented in Eq (5) and Eq (9), the corresponding training loss functions are detailed in Eq (6) and Eq (18), where motion uncertainty is modeled using a Kalman filter, which has an analytical solution, and the weighting schemes are defined in Eq (4) and Eq (10). In addition, the gating mechanism used to balance long‑term and short‑term memory has been mathematically formalized in Eq (14), Eq (15) and Eq (16), including the specific activation functions and normalization strategies about query and key, with thorough explanations provided in the paragraphs. The pseudocode in Section 3 has also been updated accordingly to ensure all key steps are fully specified and reproducible.

2). The author(s) introduces additional modules (uncertainty prediction, dual memory mechanisms), but there is no discussion of computational overhead. How does UAMP scale in terms of memory footprint and inference latency compared to SAM2?

Response: We appreciate the reviewer for this comment. In the revised manuscript, we have supplemented a detailed discussion on the computational overhead introduced by the proposed uncertainty prediction and memory management modules. We quantitatively compare the proposed UAMP framework with the baseline SAM2 model in terms of memory footprint and inference latency, and analyze the computational complexity and scalability of our method. The corresponding runtime analysis have been added in Section 4.2.2 to clearly demonstrate the efficiency and practicality of UAMP while maintaining performance improvement, which is detailed in Table 9.

3). Although recent SOTA methods (Cutie-bas, LiVOS, SAM2.1, SAM2.1Long) are included, the evaluation could be expanded: Report per-class or per-category breakdowns of J&F improvements to substantiate claims that UAMP excels in crowded or occluded scenarios.

Response: We sincerely appreciate the constructive suggestion on providing more detailed breakdowns to further verify the advantages of UAMP in crowded and occluded scenarios.

However, we would like to clarify that the semi-supervised VOS is inherently a class-agnostic task, where given an initial mask, bounding box, or interactive clicks in the first frame, the model only needs to separate the specified target from the background, without relying on semantic category labels. Secondly, none of the compared SOTA methods (Cutie-bas, LiVOS, SAM2.1, SAM2.1Long) reports per-class or per-category J&F metrics in their original papers or official implementations. The official evaluation protocols of the benchmark datasets (e.g., LVOS) also do not provide pre-defined class/category splits for computing such fine-grained per-class statistics. Manually defining arbitrary categories and computing breakdowns alone would lead to incomparable and non-reproducible results against existing SOTAs.

To substantiate our claim that UAMP performs favorably in crowded and occluded scenarios, we evaluated the proposed method on six different datasets, including MOSE, SA-V, LVOSv1, LVOSv2, DAVIS and YouTube-VOS, in which the LVOS is designed for long-term video object segmentation, MOSE, the complex real-life dataset, contains the crowded and occluded scenarios, and SA-V has small, occluded, and reappearing objects in the video sequences. Following the standard evaluation protocol widely accepted in the VOS field, we report overall J&F scores in line with representative recent works. To better substantiate our claim that UAMP performs favorably in crowded and occluded scenarios, we have done the qualitative and quantitative analysis on challenging sequences, highlighting the advantages of our method under complex conditions in Section 4.2.

4). The manuscript alternat

Attachment Submitted filename: Response_to_Reviewers_auresp_2.docx

# Editor report: Decision Letter 2

**DOI:** 10.1371/journal.pone.0353156.r005

**Authors:** Taikyeong Ted Jeong

3 Jun 2026

PONE-D-25-54851R2

UAMP: Consistent Video Object Segmentation with Uncertainty-Aware Memory Propagation

PLOS One

Dear Dr. Liu,

Thank you for submitting your manuscript to PLOS ONE. After careful consideration, we feel that it has merit but does not fully meet PLOS ONE’s publication criteria as it currently stands. Therefore, we invite you to submit a revised version of the manuscript that addresses the points raised during the review process.

The author must read the reviewer's comments carefully and write a rebuttal letter to address their questions. Please provide a response by resolving several major issues.

Please submit your revised manuscript by Jul 18 2026 11:59PM. If you will need more time than this to complete your revisions, please reply to this message or contact the journal office at plosone@plos.org. When you're ready to submit your revision, log on to https://www.editorialmanager.com/pone/ and select the 'Submissions Needing Revision' folder to locate your manuscript file.

Please include the following items when submitting your revised manuscript:

- A letter that responds to each point raised by the academic editor and reviewer(s). You should upload this letter as a separate file labeled 'Response to Reviewers'.
- A marked-up copy of your manuscript that highlights changes made to the original version. You should upload this as a separate file labeled 'Revised Manuscript with Track Changes'.
- An unmarked version of your revised paper without tracked changes. You should upload this as a separate file labeled 'Manuscript'.

If you would like to make changes to your financial disclosure, please include your updated statement in your cover letter. Guidelines for resubmitting your figure files are available below the reviewer comments at the end of this letter.

If applicable, we recommend that you deposit your laboratory protocols in protocols.io to enhance the reproducibility of your results. Protocols.io assigns your protocol its own identifier (DOI) so that it can be cited independently in the future. For instructions see: https://journals.plos.org/plosone/s/submission-guidelines#loc-laboratory-protocols. Additionally, PLOS ONE offers an option for publishing peer-reviewed Lab Protocol articles, which describe protocols hosted on protocols.io. Read more information on sharing protocols at https://plos.org/protocols?utm_medium=editorial-email&utm_source=authorletters&utm_campaign=protocols.

As the corresponding author, your ORCID iD is verified in the submission system and will appear in the published article. PLOS supports the use of ORCID, and we encourage all coauthors to register for an ORCID iD and use it as well. Please encourage your coauthors to verify their ORCID iD within the submission system before final acceptance, as unverified ORCID iDs will not appear in the published article. *Only*  the individual author can complete the verification step; PLOS staff *cannot*  verify ORCID iDs on behalf of authors.

We look forward to receiving your revised manuscript.

Kind regards,

Taikyeong Ted Jeong, Ph.D.

Academic Editor

PLOS One

Journal Requirements:

If the reviewer comments include a recommendation to cite specific previously published works, please review and evaluate these publications to determine whether they are relevant and should be cited. There is no requirement to cite these works unless the editor has indicated otherwise.

Please review your reference list to ensure that it is complete and correct. If you have cited papers that have been retracted, please include the rationale for doing so in the manuscript text, or remove these references and replace them with relevant current references. Any changes to the reference list should be mentioned in the rebuttal letter that accompanies your revised manuscript. If you need to cite a retracted article, indicate the article’s retracted status in the References list and also include a citation and full reference for the retraction notice.

Additional Editor Comments:

The author must read the reviewer's comments carefully and write a rebuttal letter to address their questions. Please provide a response by resolving several major issues.

[Note: HTML markup is below. Please do not edit.]

[NOTE: If reviewer comments were submitted as an attachment file, they will be attached to this email and accessible via the submission site. Please log into your account, locate the manuscript record, and check for the action link "View Attachments". If this link does not appear, there are no attachment files.]

To ensure your figures meet our technical requirements, please review our figure guidelines: https://journals.plos.org/plosone/s/figures

You may also use PLOS’s free figure tool, NAAS, to help you prepare publication quality figures: https://journals.plos.org/plosone/s/figures#loc-tools-for-figure-preparation.

NAAS will assess whether your figures meet our technical requirements by comparing each figure against our figure specifications.

# Author comment: Author response to Decision Letter 3

**DOI:** 10.1371/journal.pone.0353156.r006

18 Jun 2026

Dear Editor,

We appreciate you and the reviewers for your careful evaluation and constructive suggestions on our manuscript. We have comprehensively revised the manuscript point by point in accordance with all comments raised by the reviewers. All revisions are highlighted in the revised manuscript with track changes. A detailed point-by-point response to each comment is provided below.

Required revisions:

1) Clarification and formal definition of uncertainty modules

The manuscript should clearly define the appearance and motion uncertainty components and describe how these quantities are supervised or learned during training. The current description remains partly conceptual and does not fully specify the mathematical formulation.

Response: Thank you for your careful and constructive comment. We have further strengthened the mathematical definition and learning mechanism of appearance uncertainty and motion uncertainty to avoid conceptual ambiguity.

Specifically, we have clearly defined the formulation of appearance uncertainty in Eq (5) and its corresponding supervised loss in Eq (6), which explicitly guides the learning of appearance reliability during training. For motion uncertainty, we have provided a precise mathematical formulation in Eq (8-9). In the revised manuscript, we have also added detailed explanations for each component, clarifying how these uncertainty values are modeled, optimized, and integrated into the overall training pipeline. All components are now fully specified with explicit mathematical expressions, ensuring the description is concrete and reproducible.

2) Reproducibility of the proposed framework

Several key elements of the method require clearer specification to ensure reproducibility, including:

a) the gating mechanism used for balancing long-term and short-term memory

b) the exact formulation of the uncertainty estimation modules

c) the loss functions and weighting schemes used during training

d) the full specification of the Kalman filter used for motion scoring

Response: Thank you for your valuable comment on ensuring the reproducibility of our method. We have carefully addressed each of the key elements highlighted, with detailed specifications and explicit formulations supplemented throughout the revised manuscript:

For a) We have mathematically formalized this mechanism in Eq (14), Eq (15), and Eq. (16). Specifically, Eq. (14) clearly defines the specific normalization strategies for the query and key components, and we have provided thorough, step-by-step explanations in the accompanying paragraphs to clarify how the gating weights in Eq (16) are computed.

For b) The formulations for appearance uncertainty and motion uncertainty estimation are explicitly presented in Eq (5) and Eq (9), respectively. We have added detailed definitions of all variables and symbols in these equations, ensuring the mathematical formulation of each uncertainty module is fully specified—no conceptual ambiguity remains, and the implementation logic is clearly elaborated.

For c) The training loss functions corresponding to the uncertainty estimation modules are detailed in Eq (6) and Eq (18). Additionally, the weighting schemes that integrate these uncertainty values into the overall framework are explicitly defined in Eq (4) and Eq (10), with clear descriptions of how these weights are applied during training to optimize the model.

For d) We have supplemented comprehensive Kalman filter formulations for motion uncertainty estimation in Eq (8) and Eq (9), along with clear explanations of all symbols and variables in Section 3.3.2. Furthermore, we have provided the exact definitions of the state transition matrix, measurement matrix, process noise covariance, observation noise covariance, and initial error covariance matrix, along with their specific experimental values to fully support the reproducibility of the motion uncertainty estimation module.

In addition, the pseudocode in Section 3 has been updated accordingly to incorporate all the above specifications, ensuring every key step of the proposed method is fully detailed and can be implemented.

3) Algorithmic clarity and symbol definitions

All variables and symbols appearing in the equations should be explicitly defined. The pseudocode and equations should be sufficiently detailed to allow independent implementation.

Response: Thank you for your valuable comment. We have revised the entire manuscript to explicitly define every variable, symbol, and subscript in all equations, with clear descriptions immediately following each formula in Section 3.

In addition, we have further expanded the pseudocode and mathematical formulations with step-by-step implementation details, including parameter settings, matrix dimensions, iterative procedures, and computation flows in Algorithm 1 and Section 4 model and train details. All critical components are described with sufficient specificity to enable independent re-implementation of the proposed method in the manuscript.

4) Experimental clarity and evaluation reporting

The evaluation section would benefit from clearer reporting of experimental settings, including hyperparameters, memory bank configuration, and training schedules. Where possible, provide additional breakdown of performance (e.g., per-category or scenario-level results) to support the claims regarding improved robustness in challenging scenarios such as occlusion or crowded scenes.

Response: Thank you for your valuable comment. We have detailed a dedicated table that explicitly summarizes all key hyperparameters, including the number of memory banks, learning rate schedule, batch size, optimizer settings, and other related configurations in Table 1 and explained the train details part in Section 4.1.2.

About providing the additional performance, we sincerely appreciate the constructive suggestion on providing more detailed breakdowns to further verify the advantages of UAMP in crowded and occluded scenarios. However, we would like to clarify that the semi-supervised VOS is inherently a class-agnostic task, where given an initial mask, bounding box, or interactive clicks in the first frame, the model only needs to separate the specified target from the background, without relying on semantic category labels. Secondly, none of the compared SOTA methods (Cutie-bas, LiVOS, SAM2.1, SAM2.1Long) reports per-class or per-category J&F metrics in their original papers or official implementations. The official evaluation protocols of the benchmark datasets (e.g., LVOS) also do not provide pre-defined class/category splits for computing such fine-grained per-class statistics. Manually defining arbitrary categories and computing breakdowns alone would lead to incomparable and non-reproducible results against existing SOTAs.

To substantiate our claim that UAMP performs favorably in crowded and occluded scenarios, we evaluated the proposed method on six different datasets, including MOSE, SA-V, LVOSv1, LVOSv2, DAVIS and YouTube-VOS, in which the LVOS is designed for long-term video object segmentation, MOSE, the complex real-life dataset, contains the crowded and occluded scenarios, and SA-V has small, occluded, and reappearing objects in the video sequences. Following the standard evaluation protocol widely accepted in the VOS field, we report overall J&F scores in line with representative recent works. To better substantiate our claim that UAMP performs favorably in crowded and occluded scenarios, we have done the qualitative and quantitative analysis on challenging sequences, highlighting the advantages of our method under complex conditions in Section 4.2.

5) Data availability statement

One reviewer indicated concerns regarding the availability of underlying data. Please ensure that all data required to reproduce the reported findings are publicly available or clearly described in accordance with PLOS ONE’s data availability policy.

Response: Thank you for your reminder regarding the data availability policy. We fully comply with the requirements of PLOS ONE and confirm that all datasets used in this study are publicly accessible standard benchmarks, including MOSE, SA-V, LVOS, DAVIS, and YouTube-VOS, which are widely adopted in the video object segmentation community and freely available to the research community. And for training our model,

We mixed about 1% SA-V dataset, 20% MOSE dataset and 20% LVOS dataset for the training dataset, specifically:

1) The SA-V dataset is composed of the middle 20 videos from sav_000491 to sav_000510 of the sav_trian.tar file, which can be download form the link https://ai.meta.com/datasets/segment-anything-video-downloads/.

2�The first 300 videos are extracted from the MOSE dataset according to the meta_train.json file contained in the train.tar.gz dataset, which can be freely downloaded from the link https://huggingface.co/datasets/FudanCVL/MOSE/tree/main.

3) The last 50 videos are chosen from the LVOS v1 dataset and last 150 videos from the LVOS v2 dataset according to the meta.json contained in the train.zp file, which can be freely downloaded from the link https://lingyihongfd.github.io/lvos.github.io/dataset.html.

And we have added the training data details in the section 4.1.2, and the updated the data availability statement in the revision.

Recommended improvements:

1) Improve the structure and clarity of section introductions so that each section and subsection begins with a brief explanation of its purpose.

Response: Thanks for this valuable comment. In the revised version, we have added concise introductory paragraphs at the beginning of the main section to briefly outline the purpose, content, or the organization of the following subsections. These short introductions help guide readers and improve the logical flow of the manuscript in Section 3. We have applied this revision across all relevant sections and subsections to ensure a formal and professional writing style.

2) Ensure consistent terminology throughout the manuscript (e.g., “uncertainty”, “change probability”, “memory bank”, etc.).

Response: Thanks for this valuable comment. We have standardized the terminology throughout the manuscript to maintain consistency. We have unified the use of “uncertainty” instead of inconsistent mixing with “change probability” and we have maintained a clear and consistent distinction between memory bank (referring to the entire memory module and storage structure) and memory (referring to the stored features and information within the bank) to remain consistent with SAM2.

3) Provide a brief discussion of computational overhead and inference latency compared with the baseline SAM2 framework.

Response: Thanks for this valuable comment. We quantitatively compare the proposed UAMP framework with the baseline SAM2 model in terms of memory footprint and inference latency, and analyze the computational complexity and scalability of our method. The corresponding runtime analysis have been added in Section 4.2.2 to clearly demonstrate the efficiency and practicality of UAMP while maintaining performance improvement, which is detailed in Table 9.

4) Review the suggested references provided by the reviewers and cite them where appropriate.

Response: We appreciate the suggested references for potential citation. We have carefully reviewed the research focus of each recommended work:

Paper a) centers on multi-modal feature fusion within a Transformer architecture for integrating visual and audio information to advance dense video captioning. Paper b) explores the design and technological roadmap for developing zero‑emission electric vessels. Paper c) aims at investigating the effect of cigarette smoke exposure on the systemic circulation and local airway 5-HT levels as well as MAO-mediated oxidative pathway using a cigarette smoke-exposed rat model. Paper d) presents device-level simulation and analysis of In₀.₅₃Ga₀.₄₇As implant‑free quantum‑well structures in microelectronics.

Our work targets video object segmentation (VOS), a core task in video understanding that aims to achieve pixel‑level foreground object separation and consistent tracking across consecutive video frames. Given that the above references belong to different research domains, including video captioning, marine engineering, biomedicine, and microelectronic device design, they are not thematically or methodologically aligned with our focus on video object segmentation. Accordingly, we respectfully consider these references less relevant to the present study. We will continue to enrich our literature review by incorporating more state‑of‑the‑art and representative studies dedicated to video object segmentation to better contextualize our contributions for future work.

-------------------------------------------------------------------------------------------------------

Reviewer #4

1). The pseudocode in Section 3 is a good addition, but some steps remain underspecified. For example:

a. The gating mechanism for balancing long-term and short-term memory is described qualitatively, but the exact mathematical formulation (activation function, normalization strategy) is missing.

b. The uncertainty estimation modules (appearance vs. motion) are introduced, but the training loss functions and weighting schemes are not fully detailed.

Response: Thanks for your constructive and valuable comments. We have further refined the relevant descriptions and mathematical formulations to clarify the underspecified details in the pseudocode, specifically:

For a, the gating mechanism used to balance long‑term and short‑term memory has been mathematically formalized in Eq (14), Eq (15) and Eq (16), where Eq. (14) clearly defines the specific normalization strategies for the query and key components, and we have provided thorough, step-by-step explanations in the accompanying paragraphs to clarify how the gating weights in Eq (16) are computed.

For b, we have updated the uncertainty estimation modules in Sections 3.3.1 and 3.3.2. The formulations for appearance uncertainty and motion uncertainty estimation are clearly presented in Eq (5) and Eq (9), the corresponding training loss functions are detailed in Eq (6) and Eq (18), where motion uncertainty is modeled using a Kalman filter, which has an analytical solution, and the weighting schemes are defined in Eq (4) and Eq (10). The pseudocode in Section 3 has also been updated accordingly in the revision, to ensure all key steps are fully specified and reproducible.

2). The author(s) introduces additional modules (uncertainty prediction, dual memory mechanisms), but there is no discussion of computational overhead. How does UAMP scale in terms of memory footprint and inference latency compared to SAM2?

Response: We appreciate the reviewer for this comment. In the revised manuscript, we have supplemented a detailed discussion on the computational overhead introduced by the proposed uncertainty prediction and memory management modules. We quantitatively compare the proposed UAMP framework with the baseline SAM2 model in terms of memory footprint and inference latency, and analyze the computational complexity and scalability of our method. The corresponding runtime analysis have been added in Section 4.2.2 to clearly demonstrate the efficiency and practicality of UAMP while maintaining performance improvement, which is detailed in Table 9.

3). Although recent SOTA methods (Cutie-bas, LiVOS, SAM2.1, SAM2.1Long) are included, the evaluation could be expanded: Report per-class or per-category breakdowns of J&F improvements to substantiate claims that UAMP excels in crowded or occluded scenarios.

Response: We sincerely appreciate the constructive suggestion on providing more detailed breakdowns to further verify the advantages of UAMP in crowded and occluded scenarios.

However, we would like to clarify that the semi-supervised VOS is inherently a class-agnostic task, where given an initial mask, bounding box, or interactive clicks in the first frame, the model only needs to separate the specified target from the background, without relying on semantic category labels. Secondly, none of the compared SOTA methods (Cutie-bas, LiVOS, SAM2.1, SAM2.1Long) reports per-class or per-category J&F me

Attachment Submitted filename: Response_to_Reviewers_auresp_3.docx

# Editor report: Decision Letter 3

**DOI:** 10.1371/journal.pone.0353156.r007

**Authors:** Taikyeong Ted Jeong

21 Jun 2026

UAMP: Consistent Video Object Segmentation with Uncertainty-Aware Memory Propagation

PONE-D-25-54851R3

Dear Dr. Liu,

We’re pleased to inform you that your manuscript has been judged scientifically suitable for publication and will be formally accepted for publication once it meets all outstanding technical requirements.

Within one week, you’ll receive an e-mail detailing the required amendments. When these have been addressed, you’ll receive a formal acceptance letter and your manuscript will be scheduled for publication.

An invoice will be generated when your article is formally accepted. Please note, if your institution has a publishing partnership with PLOS and your article meets the relevant criteria, all or part of your publication costs will be covered. Please make sure your user information is up-to-date by logging into Editorial Manager at Editorial Manager® and clicking the ‘Update My Information' link at the top of the page. For questions related to billing, please contact billing support.

If your institution or institutions have a press office, please notify them about your upcoming paper to help maximize its impact. If they’ll be preparing press materials, please inform our press team as soon as possible -- no later than 48 hours after receiving the formal acceptance. Your manuscript will remain under strict press embargo until 2 pm Eastern Time on the date of publication. For more information, please contact onepress@plos.org.

Kind regards,

Taikyeong Ted Jeong, Ph.D.

Academic Editor

PLOS One

Additional Editor Comments (optional):

Confirmed, the current manuscript - to reflect all the reviewer's feedback. Many parts have been modified, and you can view detailed responses to each comment.

# Editor report: Acceptance letter

**DOI:** 10.1371/journal.pone.0353156.r008

**Authors:** Taikyeong Ted Jeong

PONE-D-25-54851R3

PLOS One

Dear Dr. Liu,

I'm pleased to inform you that your manuscript has been deemed suitable for publication in PLOS One. Congratulations! Your manuscript is now being handed over to our production team.

At this stage, our production department will prepare your paper for publication. This includes ensuring the following:

* All references, tables, and figures are properly cited

* All relevant supporting information is included in the manuscript submission,

* There are no issues that prevent the paper from being properly typeset

You will receive further instructions from the production team, including instructions on how to review your proof when it is ready. Please keep in mind that we are working through a large volume of accepted articles, so please give us a few days to review your paper and let you know the next and final steps.

Lastly, if your institution or institutions have a press office, please let them know about your upcoming paper now to help maximize its impact. If they'll be preparing press materials, please inform our press team within the next 48 hours. Your manuscript will remain under strict press embargo until 2 pm Eastern Time on the date of publication. For more information, please contact onepress@plos.org.

You will receive an invoice from PLOS for your publication fee after your manuscript has reached the completed accept phase. If you receive an email requesting payment before acceptance or for any other service, this may be a phishing scheme. Learn how to identify phishing emails and protect your accounts at https://explore.plos.org/phishing.

If we can help with anything else, please email us at customercare@plos.org.

Thank you for submitting your work to PLOS ONE and supporting open access.

Kind regards,

PLOS ONE Editorial Office Staff

on behalf of

Professor Taikyeong Ted Jeong

Academic Editor

PLOS One
